# Clinical spectrum, genetic complexity and therapeutic approaches for retinal disease caused by *ABCA4* mutations

**DOI:** 10.1016/j.preteyeres.2020.100861

**Published:** 2020-04-09

**Authors:** Frans P.M. Cremers, Winston Lee, Rob W.J. Collin, Rando Allikmets

**Affiliations:** a Department of Human Genetics, Radboud University Medical Center, PO Box 9101, 6500 HB, Nijmegen, the Netherlands; b Donders Institute for Brain, Cognition and Behaviour, Radboud University Medical Center, PO Box 9104, 6500 HE, Nijmegen, the Netherlands; c Department of Ophthalmology, Columbia University, New York, NY, 10032, USA; d Department of Genetics & Development, Columbia University, New York, NY, 10032, USA; e Department of Pathology & Cell Biology, Columbia University, New York, NY, 10032, USA

**Keywords:** Stargardt disease, *ABCA4*-associated retinopathy, Allelic heterogeneity, Autofluorescence, Phenocopies, Hypomorphic variant, Penetrance, Splice defects, Pseudoexon, Structural variant, Therapy

## Abstract

The ABCA4 protein (then called a “rim protein”) was first identified in 1978 in the rims and incisures of rod photoreceptors. The corresponding gene, *ABCA4*, was cloned in 1997, and variants were identified as the cause of autosomal recessive Stargardt disease (STGD1). Over the next two decades, variation in *ABCA4* has been attributed to phenotypes other than the classically defined STGD1 or fundus flavimaculatus, ranging from early onset and fast progressing cone-rod dystrophy and retinitis pigmentosa-like phenotypes to very late onset cases of mostly mild disease sometimes resembling, and confused with, age-related macular degeneration. Similarly, analysis of the *ABCA4* locus uncovered a trove of genetic information, including >1200 disease-causing mutations of varying severity, and of all types – missense, nonsense, small deletions/insertions, and splicing affecting variants, of which many are located deep-intronic. Altogether, this has greatly expanded our understanding of complexity not only of the diseases caused by *ABCA4* mutations, but of all Mendelian diseases in general. This review provides an in depth assessment of the cumulative knowledge of ABCA4-associated retinopathy – clinical manifestations, genetic complexity, pathophysiology as well as current and proposed therapeutic approaches.

## 1. Historical perspective

Hereditary dystrophies of the macula reminiscent of autosomal recessive Stargardt disease (STGD1) have been documented from as early as the end of the 19th century (Lang, 1885). However, Karl Bruno Stargardt, of the University of Strasbourg, is recognized as having first published the most comprehensive clinical description, including fundus drawings, of seven patients from two families in 1909 (Stargardt, 1909). In this seminal article, Stargardt concluded that the patients had a genetic, neuroepithelial disease that initially affected cones, followed by the retinal pigment epithelium (RPE) and subsequently, the underlying choroid.

Decades later, in 1962, the Swiss ophthalmologist Adolph Franceschetti coined the term “fundus flavimaculatus” in a cohort of patients he described as having a “peculiar fundus affection”, to colleagues at a meeting of the German Ophthalmological Society in Hamburg. In collaboration with Jules Francois from Ghent University, Franceschetti further described 36 cases in two articles published in 1965 (Franceschetti, 1965; Franceschetti and Francois, 1965). In the latter article, Franceschetti suspected that these patients had the same condition as earlier described by Stargardt opining, “if the foci (flecks) are localized at the posterior pole of the eye and accompanied by macular affection, the distinction from Stargardt disease may be difficult or even impossible”. Further evidence arrived two years later when Alex E. Krill and Bertha A. Klien documented the presence of delayed dark adaptation in a similar cohort of patients whom they described as having “flecked retina syndrome” (Klien and Krill, 1967). They presented the first histopathological analysis of an eye from a patient in the third decade of life concluding that the primary abnormality of this condition lies within the RPE. In 1971, August F. Deutman in great detail described 25 STGD1 and six fundus flavimaculatus families (Deutman, 1971). In 1975, Francois confirmed the connection between all of these disorders citing characteristic heritable, clinical and electrophysiological features (François et al., 1975). Although regarded as a distinct disease entity, Gerald Fishman, of the Illinois Eye and Ear Infirmary in Chicago, recognized differences in clinical expression and accordingly established a four-tier classification system (Fishman, 1976) that to this day remains influential to ophthalmologists around the world. From his investigation of fundoscopic and electrophysiological findings of 38 patients, Fishman classified the severity of STGD1 across the following stages:

Stage I: Confined central macular lesions ranging from irregular pigmentary mottling to well-defined lesions of RPE atrophy with a characteristic “beaten-bronze” or “snail-slime” appearance underlying central or paracentral scotomas.

Stage 2: Presence of yellow fundus flecks, some of which may be resorbed, beyond 1 disc diameter from the fovea extending beyond the vascular arcades and regions nasal to the optic disc.

Stage 3: Diffusely resorbed flecks and choriocapillaris atrophy within the macula.

Stage 4: Extensive choriocapillaris atrophy throughout the posterior pole resulting in moderate to severe restriction of peripheral fields.

Numerous clinical studies have since been published adding to the growing body of knowledge. However, the “modern era” of our understanding of STGD1 was precipitated by parallel breakthroughs in the basic sciences beginning with the initial characterization of the ABCA4 protein in 1978, which was initially referred to as “rim” protein for its localization in rod photoreceptor outer segments and incisures (Papermaster et al., 1976). The genetic locus was mapped to 1p13 in mid-1990s (Anderson et al., 1995; Gerber et al., 1995; Kaplan et al., 1993) and, finally, the gene was cloned in 1997 (Allikmets et al., 1997). Taken together, cumulative advances over the last three decades have provided a defining foundation for understanding what we now know to be the most common inherited Mendelian eye disorder in the world.

## 2. Clinical hallmarks of *ABCA4*-associated retinopathy

### 2.1. Ophthalmic examination

In the “classic” presentation of STGD1, central vision loss typically becomes apparent between adolescence and young adulthood. However, the age of onset varies extensively, where a proportion of individuals start experiencing delayed vision loss between the 4th and 7th decades of life (Gerber et al., 1995; Lambertus et al., 2016; Runhart et al., 2018; Runhart et al., 2019; Westeneng-van Haaften et al., 2012; Yatsenko et al., 2001; Zernant et al., 2017; Zernant et al., 2018). The exact age of disease onset is often difficult to determine, as many patients—particularly children—may be unaware of their visual impairment or have preserved central vision due to functional sparing of the fovea (Bax et al., 2019b; Fujinami et al., 2013b; Nakao et al., 2012; Runhart et al., 2019; van Huet et al., 2014). In general, *ABCA4*-associated retinopathy subtypes that manifest early in life tend to progress more rapidly, while a later age of onset is associated with a milder prognosis (Fujinami et al., 2015; Tanaka et al., 2018; Zernant et al., 2017). The initial symptoms of *ABCA4*-associated retinopathy typically begin with central or pericentral vision loss and may include difficulty with dark adaptation as the disease progresses in severity (Fishman et al., 1999; Kang Derwent et al., 2004; Klien and Krill, 1967; Salvatore et al., 2014; Scholl et al., 2002). The latter is seldomly reported by patients but generally recognized upon careful inquiry in the clinic. Other symptoms may include impaired color discrimination and photophobia (Klevering et al., 2002; Rotenstreich et al., 2003). Examination of the anterior segment and vitreous is generally unremarkable. As most patients present to the clinic in relatively early disease stages, fundoscopic findings may be subtle and rarely include typical features of advanced retinal degeneration such as a pale, disc pallor or extensive attenuation of the retinal vessels.

### 2.2. Family history and inheritance patterns

Although STGD1 is an autosomal recessive disease caused by bi-allelic variants in *ABCA4* (Allikmets et al., 1997), several factors need to be considered when taking a thorough family history and constructing a pedigree. Due to its clinical heterogeneity, many phenocopies—retinal disease caused by other genes resembling *ABCA4-*associated retinopathy—exist, including dominantly inherited conditions. Furthermore, some of the more prevalent dominant masqueraders exhibit incomplete penetrance across generations which may further simulate autosomal recessive inheritance in a pedigree (Chapi et al., 2019; Michaelides et al., 2005; Shankar et al., 2016; Sohocki et al., 1998). One should be aware of including relatives with age-related macular degeneration (AMD) in the assessment of family history due to its overlapping symptoms with late-onset STGD1 including central vision loss, especially as it has been reported that the prevalence of AMD is higher in families with STGD1 (Allikmets, 2000; Souied et al., 1999). Lastly, pseudodominant inheritance has been extensively reported in families segregating three (Beit-Ya’acov et al., 2007; Huckfeldt et al., 2016; Shroyer et al., 2000), or even four *ABCA4* alleles within a single family (Klevering et al., 2002; Lee et al., 2016; Runhart et al., 2018). A further heightened awareness is warranted when working with patients of consanguineous families, mostly from culturally and geographically isolated populations due to the founder alleles resulting in frequent enrichment of homozygosity (Ducroq et al., 2006; Falfoul et al., 2018; Lee et al., 2017).

### 2.3. The diagnostic triad

Despite the breadth of clinical heterogeneity associated with *ABCA4*-associated retinopathy, all patients share a common genetic etiology caused by mutations in a single gene, and a set of near ubiquitous clinical features. The following are three diagnostic clinical findings that, when occurring together in a patient, are highly indicative of ABCA4-associated retinopathy:

#### 2.3.1. Macular affection

**Fig. 1.**
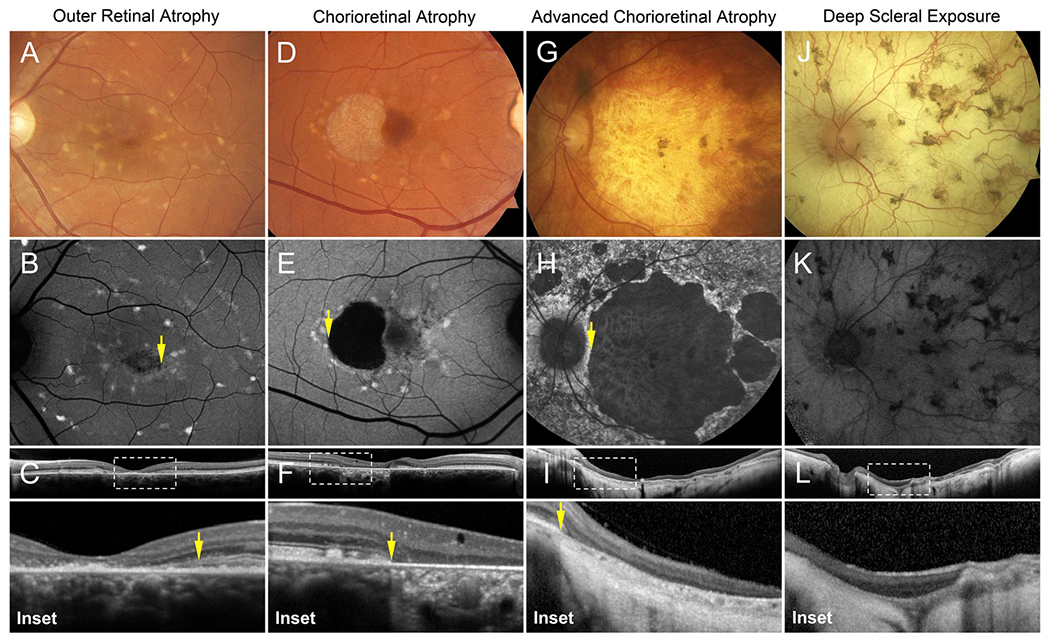
Stages of atrophy progression in *ABCA4*-associated retinopathy. Fundus photographs with corresponding short wavelength autofluorescence (SW-AF) images and foveal spectral domain-optical coherence tomography (SD-OCT) scans depicting the progressive stages of macular atrophy in *ABCA4*-associated retinopathy. (A) Early lesions exhibit a mottled appearance on funduscopy and (B) diffusely decreased autofluorescence on SW-AF imaging. (C) An apparent loss of the photoreceptor-attributable ellipsoid zone (EZ) band and appearance of hyper-reflective debris can be observed by SD-OCT within the lesion at this stage. (D) Lesions in the chorioretinal atrophy stage exhibit the canonical beaten-bronze appearance, are well-delineated and enable visibility of underlying choroidal vessels. (E) This stage is also uniquely characterized by a homogeneous and complete loss of autofluorescence; (F) A marked thinning of the retinal pigment epithelium (RPE) layer resulting in an increased transmission of the SD-OCT signal (F, inset) is typically present at this stage. (G, H) Continued progression of atrophy extends across the macula and posteriorly, sequentially involving the choriocapillaris, Sattler and Haller layers of the choroid (I, inset). (J, K) The end-stage of widespread degeneration results in a complete loss of outer retinal and choroidal layers (L) resulting in a visibility of the underlying sclera. The discernible edge of the atrophic lesion and its corresponding position on SD-OCT are denoted by yellow arrows (For interpretation of the references to color in this figure legend, the reader is referred to the Web version of this article).

Progressive deterioration of cellular layers originating in the central macula is a canonical feature of *ABCA4*-associated retinopathy and the principal cause of visual deterioration over time. The cellular origin of ABCA4 dysfunction and temporal sequence of cellular degeneration has been and remains a contentious issue (Duncker et al., 2014; Greenstein et al., 2017; Lenis et al., 2018; Song et al., 2015a; Sparrow et al., 2012). Clinically, atrophic changes typically begin with loss of the outer retinal layers (retinal pigment epithelium - RPE and photoreceptor-attributable ellipsoid zone) and, in cases that progress to more advanced stages, invariably involve the choriocapillaris. Further deterioration of the underlying choroidal layers (Sattler and Haller) may occur in response to the rapid demise of RPE (Bertelsen et al., 2014; Muller et al., 2017; Tanaka et al., 2018) or progressively to a stage at which the underlying sclera may be visible on fundoscopy depending on the duration of disease (Fig. 1) (Lee et al., 2018).

#### 2.3.2. Fundus flecks

**Fig. 2.**
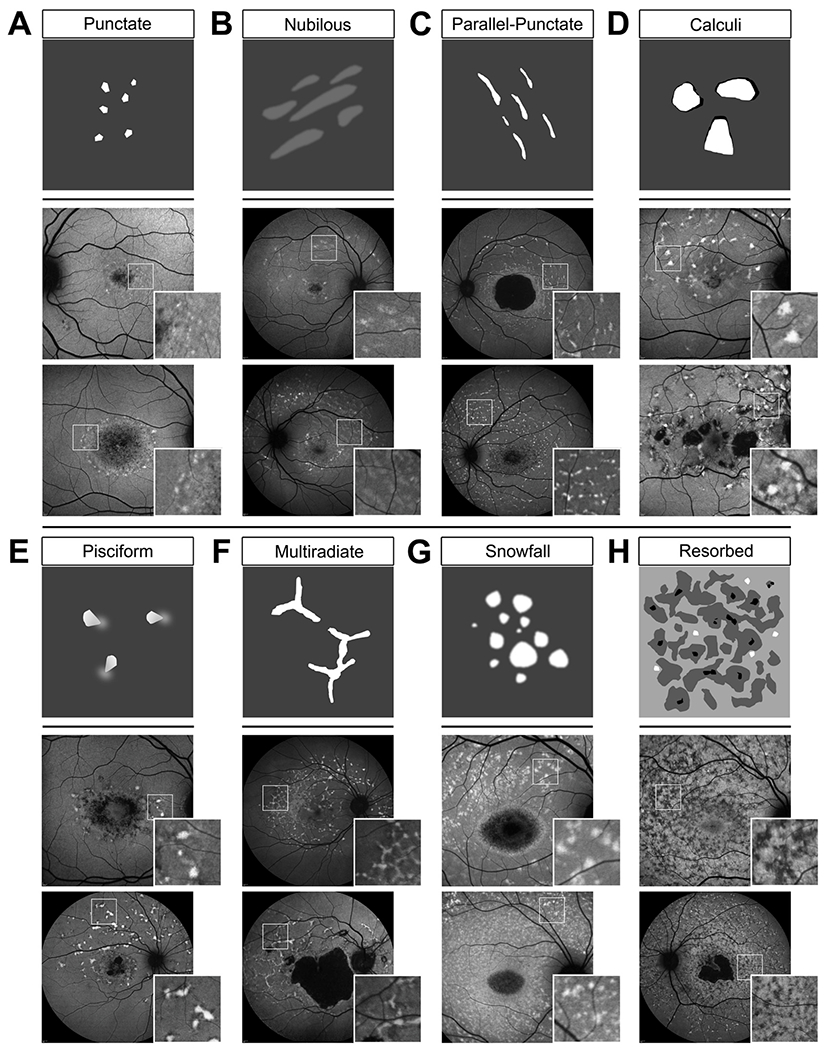
Morphological spectrum of fundus flecks in *ABCA4*-associated retinopathy. Lipofuscin-laden flecks deposited across the fundus exhibit a yellow appearance on fundoscopy and an intense autofluorescent signal on short wavelength autofluorescence (SW-AF) imaging. The collective spatio-temporal pattern of flecks and their individual morphology (A–G) vary across disease stage and genotypic trajectories. Areas of resorbing flecks become hypoautofluorescent and coalescence into a heterogeneous pattern across the posterior pole.

The augmented accumulation of RPE lipofuscin occurs throughout the retina; however, this process can also manifest locally giving rise to one of the most recognizable features of *ABCA4*-associated retinopathy—flecks. This feature is most conspicuous on short-wavelength autofluorescence (SW-AF) as intensely fluorescent foci distributed across the macula or extending far across the posterior pole at more advanced disease stages (Chen et al., 2019; Cideciyan et al., 2015). Histopathological observations have attributed flecks to engorged RPE cells, stacked aggregations of RPE cells or remnants of dying RPE (Eagle et al., 1980; Lopez et al., 1990). However more recently, Sparrow et al. proposed that flecks may be extracellular accumulations of unphagocytized outer segments from longitudinal observations of their structural (SD-OCT) and autofluorescence (SW-AF and near infrared-AF) characteristics over time (Sparrow et al., 2015). The ubiquitous presence of flecks in *ABCA4*-associated retinopathy is an invaluable diagnostic asset in the clinic; however, the inherent variation in their individual size and morphology, irregular patterns across the fundus and spatial evolution over time, may conceal crucial information about disease etiology and prognosis. The most frequent fleck patterns of *ABCA4*-associated retinopathy are illustrated in Fig. 2. Unfortunately, very few studies have comprehensively explored the significance of fleck characteristics to date. This is likely due to the community’s preoccupation with lesion-centric characteristics and atrophy progression. Functionally, patients exhibit decreased visual sensitivity over flecked areas but their contribution to the centrally progressing atrophy is uncertain (Querques et al., 2006; Verdina et al., 2012). Flecks are generally regarded as a biomarker of disease severity based on their emergence along the central and peripheral axis of the *ABCA4*-associated retinopathy retina (Cukras et al., 2012). Teussink et al. sought to study the effect of light on the progression of *ABCA4*-associated retinopathy by comparing fleck accumulation in an eye of patients compared to the fellow eye that was continuously patched over one year, but the results were variable (Teussink et al., 2015). The causal role of flecks in the pathophysiology of *ABCA4*-associated retinopathy is thus far uncertain and much remains to be elucidated. Particular insight may lie in their longitudinal patterns—centrifugal (Cukras et al., 2012) versus zonal (Paavo et al., 2019; Sparrow et al., 2015). Furthermore, flecks are highly dynamic and exhibit rapid changes, which may better define the “leading disease front” of *ABCA4*-associated retinopathy as compared to the central lesion of atrophy.

#### 2.3.3. Peripapillary sparing

**Fig. 3.**
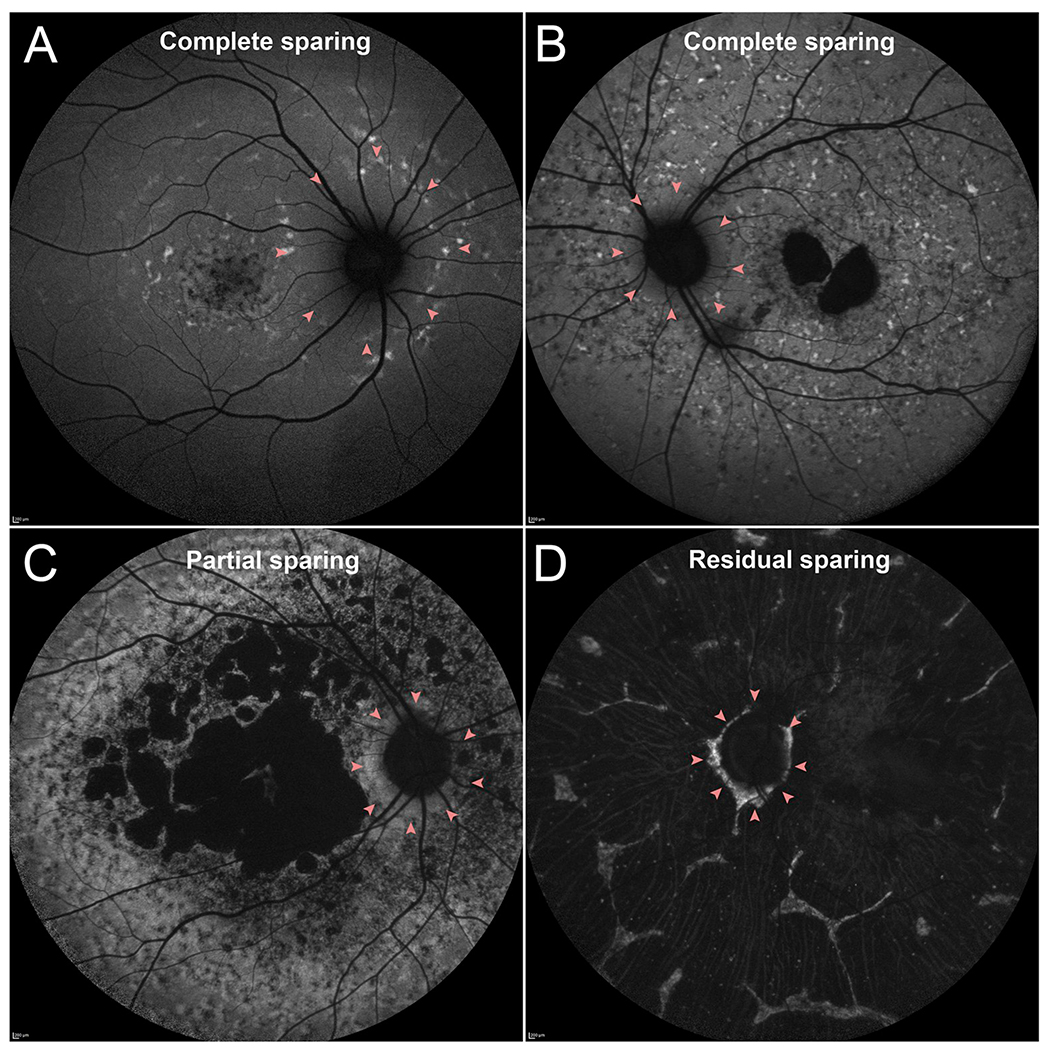
Disease-sparing of the peripapillary region in *ABCA4*-associated retinopathy. Sparing of the peripapillary region around the optic nerve (magenta arrowheads) from disease changes is a characteristic feature of *ABCA4*-associated retinopathy and becomes apparent as flecks extend centripetally across the posterior pole of the retina (A, B). Resistance of this region persists into the late atrophic stages and gradually becomes affected (C). The presence of residual circumpapillary tissue may be discernible despite the widespread atrophy (D).

Perhaps the most unusual feature amongst the triad is the observation that the proximal tissue surrounding the optic nerve is spared of disease changes in *ABCA4*-associated retinopathy (Fig. 3) (Cideciyan et al., 2005). Other diseases, most notably *PRPH2*- and *ROM1*-associated pattern dystrophy and *RDH12*-associated Leber congenital amaurosis (LCA), have been reported to exhibit a similar manifestation although not as consistent as in *ABCA4*-associated retinopathy (Duncker et al., 2015c; Garg et al., 2017; Ma et al., 2019). Sparing of this region often persists in *ABCA4*-associated retinopathy, both structurally and functionally but progressively lost at later disease stages; however, its etiological basis is unknown. Several theories have been proposed including the disc membrane–load hypothesis, light-load hypothesis, lipofuscin-clearance hypothesis and neurotrophic factors hypothesis, although all are largely inferential (Cideciyan et al., 2005).

Each feature of the *ABCA4*-associated retinopathy triad exhibits stage-dependent changes, which have been documented independently. Effective modeling of *ABCA4*-associated retinopathy should encompass the variability of all three features and their relationship to one another in order to acquire a deeper understanding of the condition.

### 2.4. Bull’s eye maculopathy

**Fig. 4.**
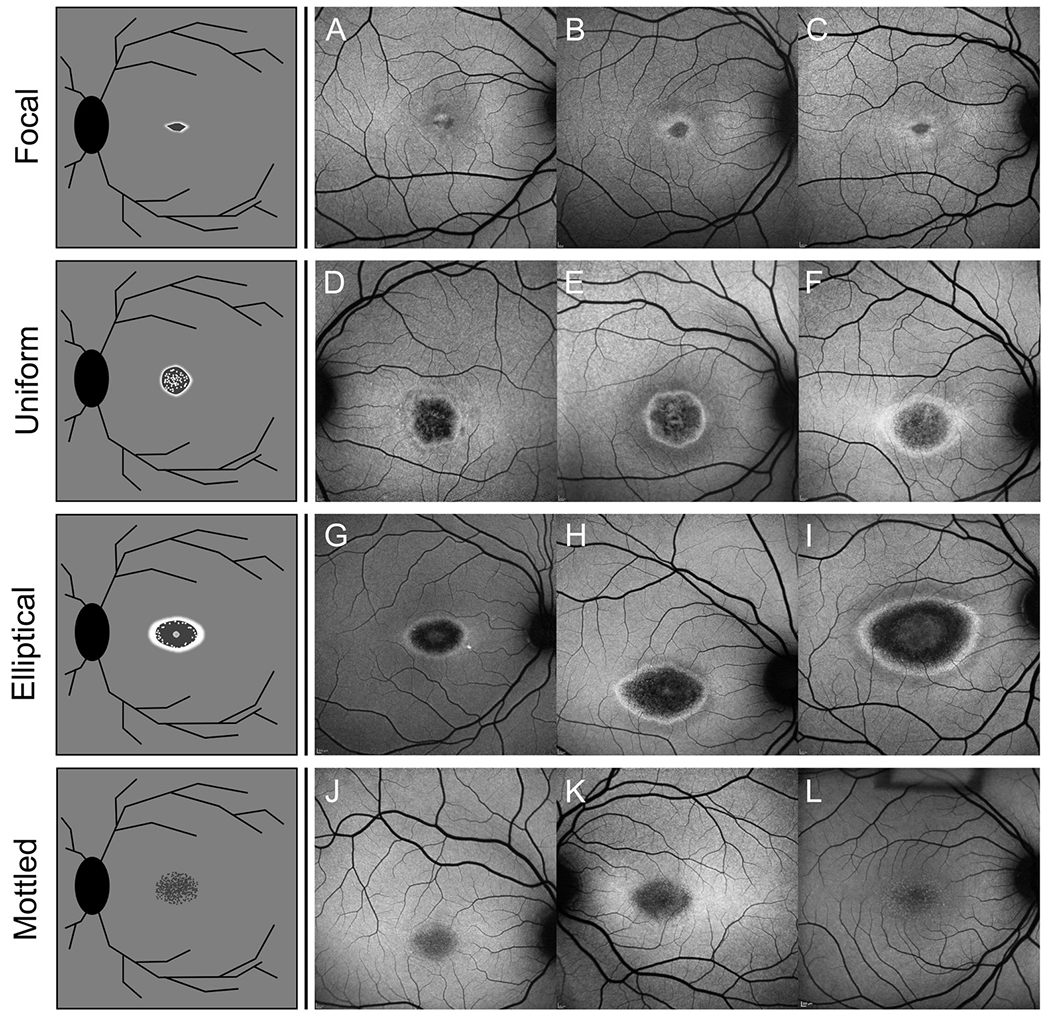
Autofluorescence subtypes of the bull’s eye maculopathy (BEM) stage of ABCA4-associated retinopathy. Confined BEM lesions are generally the earliest manifestation of macular affection in ABCA4-associated retinopathy and are highly associated with the c.5882G>A, p.(Gly1961Glu) mutation. (A–C) Small, focal lesions are typically associated with a loss of the ellipsoid zone (EZ) band and subsequent cavitation of this space in the fovea (“optical gap”). (D–F) Uniformly round BEM lesions exhibit continuous autofluorescence borders and punctate debris within the atrophy region. (G–I) Elliptical BEM lesions also exhibit smooth, continuous autofluorescent borders; however, the region inside the lesion contains less debris and are marked by a central patch of autofluorescence (“bull’s eye”) indicating prior sparing of the fovea. Much less common are centrally mottled BEM lesions (J–L) which are distinct in that they lack a hyperautofluorescent perimeter and are almost exclusive to adolescent patients.

A diagnostic exception to the triad is the Bull’s eye maculopathy (BEM) stage which accounts for up to ~20% of *ABCA4*-associated retinopathy cases presenting to the clinic and is over-represented by the c.5882G>A, p.(G1961E) variant (Celia et al., 2009). BEM is defined as a circularly confined region of atrophy beginning in the central macula. The BEM stage precedes the development of all triad features making this stage the most challenging to diagnose. Furthermore, overlapping variations of the BEM phenotype are found in many inherited macular as well as non-genetic conditions including hydroxychloroquine toxicity, infectious diseases and acute injuries, although BEM observed in *ABCA4*-associated retinopathy can be often distinguished by quantifying macular levels of autofluorescence (RPE lipofuscin) (Duncker et al., 2015b). A detailed discussion of differential diagnoses is presented in section 3, Phenocopies of ABCA4-associated retinopathy. Representative manifestations of BEM in *ABCA4*-associated retinopathy are provided in Fig. 4.

### 2.5. Early perturbations in young patients

Studies to date indicate that visual function loss may precede readily detectable fundus features in young patients on clinical exam, although asymptomatic cases due to foveal sparing can be incidentally encountered by routine examination (Khan et al., 2018; Lee et al., 2014). In a cohort of 50 young *ABCA4*-associated retinopathy patients (age ≤ 10 years), Lambertus and colleagues identified 10 individuals with visual function loss in the absence of discernible fundus abnormalities at the time of examination and reported the onset of visual acuity decline from as early as 3 years of age (Lambertus et al., 2015). Defects in color vision have also been reported in patients with early to no detectable fundus changes (Bax et al., 2019a; Vandenbroucke et al., 2015). The youngest documented case of *ABCA*4-associated retinopathy was an asymptomatic 5-year-old girl in a pseudodominant family harboring the c.5018 + 2T>C, p.(?) and c.5882G>A, p.(G1961E) alleles. At the time of examination, the girl had mildly decreased visual acuities and no fundus changes except a prominent thickening of the external limiting membrane (ELM) on OCT (Burke et al., 2013). A subsequent study (Lee et al., 2014) and several others thereafter (Bax et al., 2019a; Melillo et al., 2016; Pang et al., 2015; Park et al., 2015) corroborated the observation of ELM thickening to be a prominent feature of early stage *ABCA4*-associated retinopathy while more recently, this thickening has been attributed to the adjacent outer nuclear layer (ONL) (Khan et al., 2018). It is possible that structural changes occur prior to functional loss in patients. Considering the pathophysiology of STGD1, marginal increases in autofluorescence or microscopic perturbations in the cone or rod mosaic may be plausible outcomes to pursue in the future with advances in quantitative autofluorescence (qAF) imaging and adaptive optics-scanning laser ophthalmoscopy (AO-SLO), respectively. Doing so will shed light on the anatomic origin of STGD1 and ultimately shape therapeutic approaches.

### 2.6. Characteristics of advanced stages

Progression to the advanced stages of *ABCA4*-associated retinopathy varies in accordance with the age of disease onset. An earlier onset of disease is usually associated with a poorer prognosis in patients. In the most severe cases, generalized rod and cone function is unrecordable by ffERG, vision deteriorates to hand motion or light perception and chorioretinal atrophy extends as far as the equatorial regions of the eye and as deep as the underlying sclera. The emergence of pigmentary changes is also highly associated with advanced disease and often accompanies other indicators of retina-wide degeneration such as waxy optic disc pallor and severe attenuation of the retinal vasculature. The appearance of the pigment deposits ranges from nummular aggregations that co-localize with atrophic lesions to extensive bone spiculeshaped depositions. The appearance of the latter is identical to the pathognomonic bone-spicule pigment seen in the fundus of patients with retinitis pigmentosa (RP).

## 3. Phenocopies of *ABCA4*-associated retinopathy

The clinical expression of STGD1, an autosomal recessive disease, is extremely variable (see above). There have been three more loci, called STGD2-4, which describe genetically and phenotypically different diseases. Historically, the term “Stargardt-like macular dystrophy” was introduced in 1994 for a dominantly inherited maculopathy, which mapped to a locus on 6q (Stone et al., 1994). The *ELOVL4* gene was later cloned from this locus, which is also called STGD3. Phenotypes caused by *ELOVL4* mutations are clinically, genetically and pathophysiologically very different from the “real” Stargardt disease due to mutations in *ABCA4* (STGD1). While the macular dystrophy phenotype of the dominant forms of the disease may somewhat resemble STGD1, the recessive forms express spinocerebellar ataxia 34 (Giroux and Barbeau, 1972; Turcotte Gauthier, 2010), ichthyosis, spastic quadriplegia and, mental retardation (Aldahmesh et al., 2011), among others. All forms of the disease are due to defects in fatty acid metabolism (Agbaga et al., 2008). Another suggested independent locus for STGD2 disease was eventually discarded and included in STGD3. The locus for another “Stargardt-like” phenotype, STGD4 (Kniazeva et al., 1999), contains the *PROM1* gene. While the phenotype of patients caused by dominant *PROM1* mutations sometimes resembles *ABCA4*-associated retinopathy (Wolock et al., 2019), the recessive form resembles RP (Maw et al., 2000; Zhang et al., 2007). These terms have, unfortunately, remained in the literature and even evolved into “dominant Stargardt disease”, which is misleading and incorrect.

**Fig. 5.**
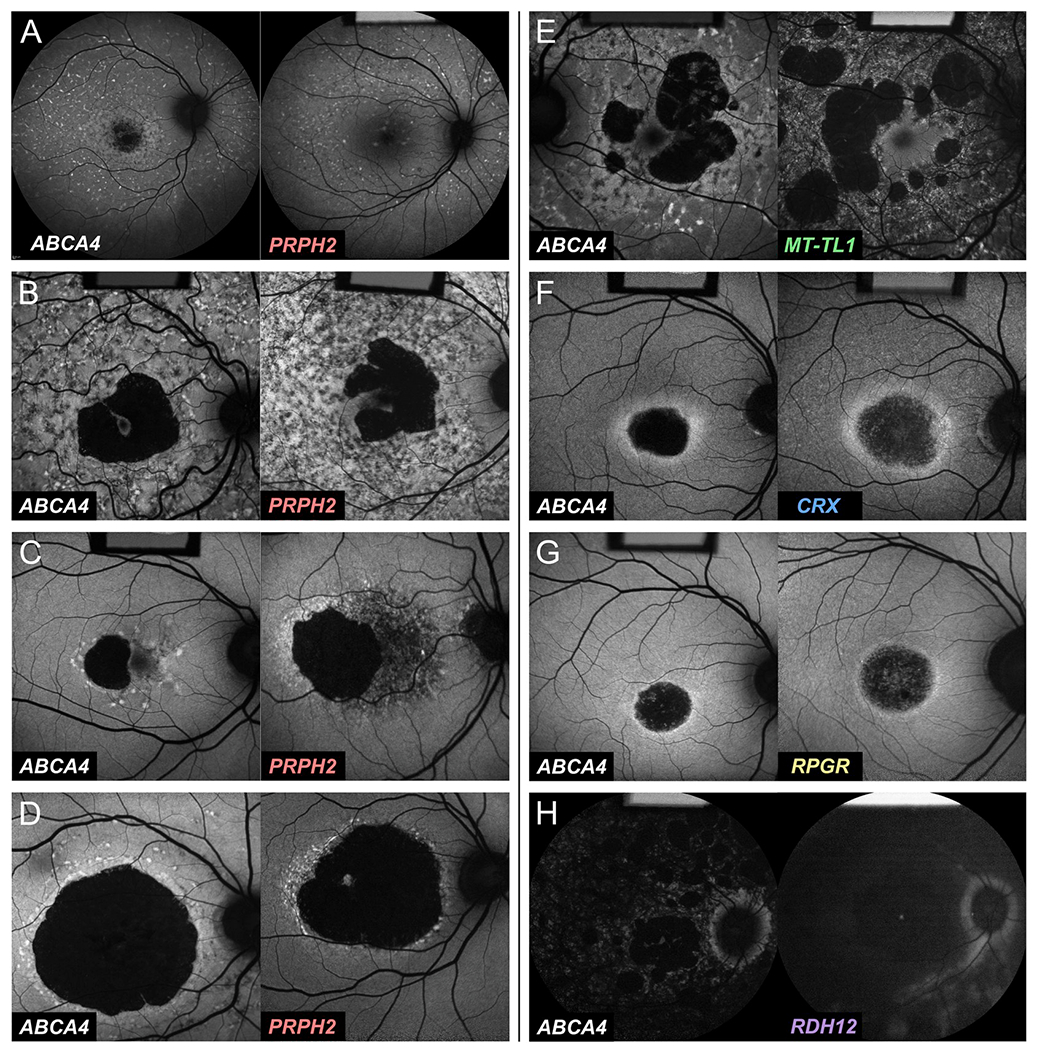
Common *ABCA4*-associated retinopathy phenocopying genes and masquerading phenotypes. (A) A 35-year-old woman harboring the c.638G>C, p.(Cys213Ser) variant in *PRPH2* with autofluorescent flecks across the posterior pole and peripapillary sparing phenocopying a 29-year-old *ABCA4*-associated retinopathy patient harboring with the C.302 + 1G> A, p.(?) variant of *ABCA4.* (B) A 62-year-old woman harboring a canonical splice site variant, c.582-1G>A, p.(?), in *PRPH2* with a confluent distribution of autofluorescent flecks across the posterior pole and “peninsular” sparing of the fovea phenocopying a 41-year-old woman harboring the c.4457C>T, p.(Pro1486Leu) and c.4793C>A, p.(Ala1486Asp) variants of *ABCA4.* (C) A 60-year-old man with pattern dystrophy harboring the c.584G>A, p.(Arg195Gln) missense variant in *PRPH2* with foveal sparing phenocopying a 40-year-old *ABCA4* disease patient harboring the hypomorphic variant, c.5603A>T, p.(Asn1868Leu), and c.4670A>G, p.(Tyr1557Cys) variants of *ABCA4.* (D) A 54-year-old man with a large, circular lesion of chorioretinal atrophy and autofluorescent flecks harboring the c.571G>T, p.(Glu191*) nonsense variant in *PRPH2* phenocopying a 44-year-old *ABCA4* disease patient harboring the hypomorphic variant, c.5603A>T, p.(Asn1868Ile), and c.4670A>G, p.(Tyr1557Cys) variants of *ABCA4.* (E) A 37-year-old man with maternally inherited diabetes and deafness (MIDD) with granular autofluorescent fleck-like depositions and “bridged” sparing of the fovea phenocopying a 42-year-old *ABCA4*-associated retinopathy patient harboring a missense, c.2971G>C, p.(Gly991Arg), and a deep-intronic, C.570 + 1798A>G, p.(Phe191Leufs*6), variant in *ABCA4.* (F) A 51-year-old woman with an elliptical BEM lesion caused by the c.449C>G, p.(Ser150*) variant in *CRX* phenocopying a 17-year-old boy with *ABCA4* disease harboring a mild missense variant, c.3113C>T, p.(Ala1038Val), and a known exon-skipping intronic variant, c.5461–10T>C, p.[Thr1821Aspfs*6,Thr1821Valfs*13] (Sangermano et al., 2016) variant in *ABCA4.* (G) A 57-year-old man with a uniform BEM lesion caused by the c.3423G>T, p.(Trp1141Cys) missense variant in *RPGR* phenocopying a 15-year-old boy with *ABCA4*-associated retinopathy harboring the c.5882G>A, p.(Gly1961Glu) and c.45G>A, p.(Trp15*) variant of *ABCA4.* (H) A 5-year-old girl with *RDHl2*-associated Leber congenital amaurosis (LCA) and peripapillary sparing homozygous for the missense variant, c.698T> A, p.(Val233Asp), phenocopying a 60-year-old man with end-stage *ABCA4*-associated retinopathy harboring the c.4139C>T, p.(Pro1380Leu) and c.4601del p.(Leu1534Trpfs*1) variants in *ABCA4*.

The common denominator for STGD1/*ABCA4*-associated retinopathy is maculopathy; i.e., the disease invariably begins in the central macula; however, as described below, the age of onset and progression are highly variable depending on the combination of specific disease-causing alleles and modifiers. Monogenic maculopathies collectively comprise a larger group; currently variants in ~38 genes are known to cause macular disease (Table 1). Some of these, e.g., *CRX, MT-TL1, PPRH2, RDH12* and *RPGR*-associated diseases, can be challenging to distinguish from various stages of *ABCA4*-associated retinopathy (Fig. 5). Moreover, some maculopathies are also caused by environmental factors, such as drug-toxicity (e.g. hydroxychloroquine) (Noupuu et al., 2016; Shroyer et al., 2001a), light damage, etc. Therefore, it is often practically impossible to determine the cause of a maculopathy without comprehensive genetic screening. Even at the most advanced retinal centers, about 10–15% of cases, who are clinically diagnosed with *ABCA4*-associated retinopathy, do not harbor disease-causing *ABCA4* variants. Most of these cases, called phenocopies, are solved by more thorough clinical assessment, including a careful examination of family history, knowledge of environmental exposure and, eventually, by genetic screening, usually by whole exome sequencing (WES) (Wolock et al., 2019).

The gene that is most often carrying (dominant) variants, which are associated with phenotypes that are indistinguishable from *ABCA4*-associated retinopathy, is *PRPH2*. Variants in *PRPH2* cause autosomal dominant pattern dystrophy and often exhibit the triad of *ABCA4*-associated retinopathy features. Since *ABCA4*-associated retinopathy is recessive, it should be quite straightforward to distinguish the two by family history. However, *PRPH2* variants often exhibit variable penetrance across generations in a family simulating recessive inheritance patterns. In addition, pattern dystrophy is a late-onset disease, which may also lead to an incorrect inference of AMD. Furthermore, pseudodominant families are frequent in *ABCA4*-associated retinopathy due to high allelic load of *ABCA4* variants in the general population (Beit-Ya’acov et al., 2007; Huckfeldt et al., 2016; Lee et al., 2016; Maugeri et al., 1999; Shroyer et al., 2000; Tracewska et al., 2019) and late onset disease expression due to reduced penetrance of variants in both *PRPH2* and *ABCA4* is well documented (Runhart et al., 2018; Yatsenko et al., 2001; Zernant et al., 2017, 2018). Therefore, also taking into account phenotype similarities, only comprehensive genetic screening can solve the causality in these cases.

In a recent study we investigated a cohort of cases where phenotypes were consistent with *ABCA4*-associated retinopathy but no disease-causing variants were found in *ABCA4* even after complete locus sequencing (Wolock et al., 2019). While variants in *PRPH2* were the most frequent cause of disease in this cohort (5–10% of all cases), and variants in another plausible gene, *PROM1*, came close second, this study also revealed variants in several other genes, usually not considered obvious causal candidates for phenocopies, including *CDHR1, CERKL, CRX* and *RPE65* (Wolock et al., 2019).

These findings are not surprising, since genetic studies have recently significantly expanded phenotype heterogeneity in many retinal diseases in addition to those caused by *ABCA4* variants. Variants in many genes, such as *CRB1* and *CRX,* cause drastically different phenotypes depending on specific mutations and inheritance pattern, where some variants cause recessive disease while others cause dominant. The tiered list of genes (based on the diagnostic triad given above), mutations in which could lead to *ABCA4* phenocopies, are given in Table 1. The important caveat, however, is the depth of clinical analysis, since some of these cases could have been distinguished from *ABCA4*-associated retinopathy already by comprehensive clinical assessment.

## 4. ABCA4 structure and function

The ABCA4 protein is an ATP-binding cassette (ABC) transporter in photoreceptor outer segments that functions in the visual cycle. More specifically, it is an N-retinylidene-phosphatidylethanolamine and phosphatidylethanolamine importer (Quazi et al., 2012; Quazi and Molday, 2013, 2014), the only known importer among mammalian ABC transporters. ABCA4 dysfunction results in accumulation of all-trans and 11-cis retinoids in photoreceptors (PRs), formation of A2E (and other bisretinoids) cumulatively called “lipofuscin”, and their accumulation mostly in the RPE (Sparrow and Boulton, 2005; Sparrow et al., 2010; Sparrow and Yamamoto, 2012). This accumulation of cytotoxic products is a hallmark, and often also the cause, of most phenotypes resulting from dysfunctional ABCA4 (Burke et al., 2014). More recently, expression of *ABCA4* has also been reported in the RPE, suggesting an additional role of the protein in this cell type that, when disturbed, could somehow contribute to *ABCA4*-associated retinopathy (Lenis et al., 2018).

The structure of ABCA4 at a high resolution is not yet described. Mammalian ABC transporters are notoriously difficult substrates for determining crystal structure (Dahl et al., 2004). The best published resolution of the native ABCA4 protein and some mutants, is 18 Å (Tsybovsky et al., 2010, 2013; Tsybovsky and Palczewski, 2014). The lack of high-resolution ABCA4 structure makes functional studies challenging, limiting experimental systems to animal models (Makelainen et al., 2019; Molday et al., 2018; Molday and Molday RS, 2016; Zhang et al., 2015) and *in vitro* assays, including ATP binding, ATPase activity and vesicular transport studies (Ahn and Molday, 2000; Beharry et al., 2004; Sun et al., 1999). The current status of the structure and (biochemical) function of ABCA4 protein is outside of the scope of this review. These aspects have been described in depth in manuscripts from the laboratories of Robert Molday and Krzysztof Palczewski, and we direct the reader to these papers for excellent overviews of the status of structure and function correlations in ABCA4 (Molday, 2015; Tsybovsky et al., 2013; Tsybovsky and Palczewski, 2014). We will address the functional studies of *ABCA4* variants affecting splicing in depth below.

## 5. Disease-causing variants in the *ABCA4* locus

Disease-causing variation in the *ABCA4* locus is extensive; there are currently >1200 disease-causing variants known and the number is rapidly growing as new cohorts, especially those of non-European descent, are screened (www.lovd.nl/ABCA4). When classified by a variant effect, the locus contains all classes of variants – missense, nonsense, indels, canonical and noncanonical splice site (NCSS) defects, deep-intronic variants and structural variants (SVs). These can be further grouped according to the proposed severity of the variant, including deleterious (i.e., complete null), severe, moderate, mild, and hypomorphic categories. The specific distinction between groups is not always unequivocal since, due to the lack of the high-resolution structure of the ABCA4 protein, the functional consequences derived from indirect assays (protein yield, ATPase and transport activity, etc.) do not always correlate exactly with resulting disease phenotypes and progression. For example, many missense alleles are deleterious (Molday et al., 2018; Zernant et al., 2017; Zhang et al., 2015), while other seemingly deleterious alleles (stop-gained and indel variants) sometimes do not result in a complete lack of function. Therefore, variant severity assignments are often not straightforward and these are constantly updated as new information becomes available from both genotype/phenotype analysis of extensive cohorts and functional studies, including those involving animal models. In section 6, we do classify variants based on pre-mRNA splicing defects.

### 5.1. Distribution of types of variants

**Fig. 6.**
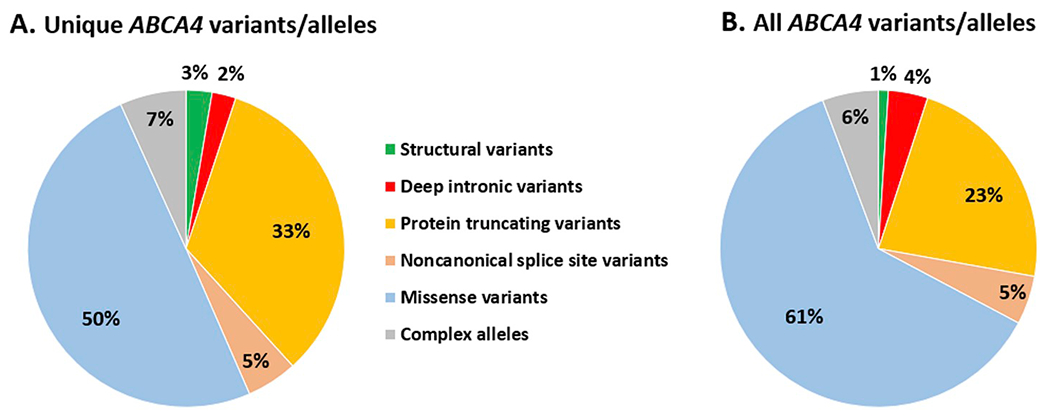
Distribution of different types of *ABCA4*-alleles. Unique (A) and all (B) *ABCA4* variants or alleles based on data collected by Cornelis et al. (2017), supplemented with deep-intronic variant and structural variant data published since then (listed in Tables 3 and 4). The contribution of each type of variant or allele is represented. Protein truncating variants comprise nonsense, frameshift and canonical splice site variants. The complex alleles represented in these pie-charts only consist of combinations of missense variants, the most frequent of which were c.[1622T>C;3113C>T] and c.[4469G>A;5603A>T]. They do not include the complex alleles that contain noncanonical splice site variants, deep-intronic variants or protein truncating variants, when present in *cis* with other variants. If these had been included, ~10% of the alleles would consist of complex alleles. Most of the structural variants, deep-intronic variants and noncanonical splice site variants also result in protein truncation.

As depicted in Fig. 6, most of the *ABCA4* variants/alleles, both in number of unique variants/alleles (50%) (Fig. 6A) and total variant/allele numbers (61%) (Fig. 6B) are missense mutations. The relatively low contribution of protein truncating mutations (23% of total and 33% of unique alleles) probably can be explained through the genotype-phenotype correlation model in which all *ABCA4*-associated retinopathy cases, except those with early-onset disease, carry at least one non-truncating mutation. The latter may also explain the relatively high frequency of NCSS variants (~5%) as their effects range from mild to deleterious. As described in more detail below, 46 different structural variants (SVs) (in ~1% of all alleles) and 35 different causal deep-intronic (DI) variants (in 4% of all alleles), have been identified in *ABCA4*-associated retinopathy cases. As the majority of genotyped *ABCA4*-associated retinopathy cases has not yet been screened for the presence of SVs and DI variants, we estimate that ~2% of all *ABCA4* alleles are SVs and ~10% are DI variants.

A significant fraction of *ABCA4* alleles (~10%) consists of more than one variant (Shroyer et al., 2001b; Zhang et al., 2015). Two of these ‘complex alleles’ are conspicuously prevalent. The c.[2588G>C; 5603A>T], p.[Gly863A1a,Gly863del;Asnl868Ile] allele is present in ~50% of all complex alleles and its relevance only came to light after the significance of p.(Asn1868Ile) was appreciated as a single causal allele, when found in *trans* with a severe or moderately severe *ABCA4* allele, see below (Runhart et al., 2018; Zernant et al., 2017). As shown previously, the c.2588G>C variant was always found in *cis* with c.5603A>T in *ABCA4*-associated retinopathy cases (Maugeri et al., 1999, 2002). Both variants are required to render the complex allele fully penetrant (Zernant et al., 2017). The c.[1622T>C;3113C>T], p.[Leu541Pro;Ala1038Val] complex allele constitutes 34% of all complex alleles. Interestingly, the single variants p.(Leu541Pro) and p.(Ala1038Val) were also found in 43 and 78 alleles, respectively, in *ABCA4*-associated retinopathy cases (Cornells et al., 2017). Based on the combinations of variants identified in *ABCA4*-associated retinopathy cases and their allele frequencies in healthy individuals, they are considered to be (moderately) severe and mild, respectively (Cornells et al., 2017). The complex allele p.[Leu541Pro;Ala1038Val] is deleterious, a complete loss-of-function allele (Zernant et al., 2017; Zhang et al., 2015).

### 5.2. Founder mutations in different populations

The *ABCA4* locus is informative with regard to multiple founder alleles; i.e. variants, which initially occurred in one geographical locale, a well-known phenomenon for geographically or culturally isolated populations (Table 2). What makes the *ABCA4* locus especially interesting is that, in addition to expected significant differences of disease-causing *ABCA4* alleles in various racial and ethnic groups, almost every nation in Europe has its “own” *ABCA4* mutation, which is much higher in frequency than in neighboring countries. Examples include the C.768G>T allele, which is very frequent in the Netherlands, but almost absent in neighboring Germany (Cremers et al., 2004), the complex allele p.[Leu541Pro;Alal038Val] in Germany (Rivera et al., 2000), which has, however, spread especially throughout Eastern Europe due to geopolitical events (Sciezynska et al., 2016; Tracewska et al., 2019; Zolnikova et al., 2017), and the p.(Arg1129Leu) variant in Spain (Valverde et al., 2006). Most of these variants have occurred once; an interesting deviation from this is the severe p.(Asn965Ser) variant, which was first described as a founder mutation in Denmark (Rosenberg et al., 2007) but subsequently also was found at a very high frequency in China (Jiang et al., 2016). Haplotype analysis confirmed that the variant occurred independently in the two regions (Jiang et al., 2016). The p.[Gly863Ala,Gly863del] variant is frequent in Western/Northern Europe (Maugeri et al., 1999, 2002) and the most frequent disease-causing *ABCA4* allele, p.(Gly1961Glu), originates from Eastern Africa, where it is found in ~10% of Somalis (Burke et al., 2012; Guymer et al., 2001). Subsequent population migration has spread p.(Gly1961Glu) throughout the world, but the allele frequency has dropped dramatically during evolution (Burke et al., 2012). The population frequency in Europe is ~0.4%, suggesting that the variant is causal in all, or at least most, populations. Other population-specific alleles are the deep-intronic c.4539 + 2001G>A variant, which is frequent in the Belgian population, comprising ~25% of all deep-intronic variants, but interestingly, is more rare in the Netherlands (Bauwens et al., 2015, 2019; Bax et al., 2015). The p.(Ala1773Val) allele is a founder variant in Mexico (Chacon-Camacho et al., 2013; Lopez-Rubio et al., 2018). The Ashkenazi Jewish population has several founder alleles, including c.4254-37_4254-15del (Beit-Ya’acov et al., 2007) and p.(Pro1380Leu) (Sharon et al., 2020). In summary, the genetic screening for pathogenic *ABCA4* alleles can often identify the ethnicity, and even the nationality, of a patient.

Although the founder alleles are already frequent within the population of European descent, they are much more prominent in various racial groups. While there is some overlap with the Caucasian population, possibly due to admixture, the disease-causing *ABCA4* mutation spectrum is significantly different, for example, in African American (Zernant et al., 2014a) and in East Asian (Hu et al., 2019; Jiang et al., 2016) populations. In both populations, the most prominent and frequent founder alleles, the c.101_106delCTTTAT, p.(Ser34_Leu35del), c.2424C>G, p.(Tyr808*), and c.6563T>C p.(Phe2188Ser), in China (Hu et al., 2019; Jiang et al., 2016) and the p.(Val989Ala), p.(Gly991Arg) and p.(Arg2107His) variants in African Americans, are almost absent from European populations (Zernant et al., 2014a). The only exception is the p.(Asn965Ser) variant, which is an independent founder allele in both Denmark and China. The population frequency of the most frequent disease-causing variant in African Americans, p.(Arg2107His), is ~2% in the general population of African American descent, suggesting that this allele can be considered hypomorphic, which is also supported by late-onset and mild disease in patients harboring this mutation (Zernant et al., 2014a). Another interesting observation is that the p.(Gly1961Glu) variant, which originates from East Africa and has a very high population frequency in Somalia, Kenya and Ethiopia (Burke et al., 2012), is almost absent in African Americans; i.e. in people of West African descent (Zernant et al., 2014a). Some populations, e.g., South Asian (Indian), have stronger admixture of European alleles (Lee et al., 2017). Most other racial and ethnic groups have not been screened in sufficient numbers of cases for valid statistical conclusions at this time.

### 5.3. Missing heritability

Recent advances in the genetic analysis of the *ABCA4* locus, including complete locus sequencing, functional assays, and introduction of the concept of hypomorphic alleles, have significantly reduced the fraction of “missing alleles”. Another important aspect in this regard is the much better clinical characterization of patients due to major advances in imaging technologies and increased experience of retinal specialists. In our centers in Nijmegen and New York, the fraction of “unsolved” cases (i.e., those with certain *ABCA4*-associated retinopathy diagnosis and one definite pathogenic allele) is <5%. The fraction of phenocopies, i.e., cases with *ABCA4*-associated retinopathy-like phenotypes but with causal mutations in genes other than *ABCA4,* is still 10–15% of all cases, even in the most advanced centers, but these are most often solved with WES, as described above (Wolock et al., 2019). So where are the remaining pathogenic *ABCA4* alleles? There are several possible scenarios:

1. Some variants can be in regulatory regions of *ABCA4,* promoter andenhancer sequences, which can affect *ABCA4* expression (Bauwens et al., 2019; Cherry et al., 2020). Some of the possibly regulatory variants have been identified, e.g., c.768 + 3223C>T and c.2919–383C>T (Bauwens et al., 2019), but more comprehensive searches and functional characterization are necessary for this category of possible mutations.
2. Yet unidentified, and/or unconfirmed, deep-intronic variants. Sequencing of the entire *ABCA4* genomic locus identifies in each patient, on average, 40 variants with an allele frequency <0.5% in population-matched control individuals, several of which could be considered causal even after thorough filtering for allele frequencies and comprehensive *in silico* analyses. Studies of monoallelic cases have identified and functionally proven 35 different deep-intronic variants to be causal, most of which are detected in more than one patient. However, since the *ABCA4* locus is extremely heterogeneous and single cases of pathogenic variants are often identified in coding sequences, the comprehensive analysis of all possibly pathogenic deep-intronic variation remains a challenging task. As described below, some deep-intronic variants cause retina-specific splicing defects (Albert et al., 2018), and almost all putative splicing variants thus far were tested in human embryonic kidney cells (HEK293T) which do not recapitulate the retina-specific splicing factors.
3. Structural variants (SVs) are (very) rare in the *ABCA4* locus (see below for deletions and duplications); however, it is likely that a small fraction of SVs is yet to be identified as short-read sequencing strategies will not identify inversions and insertions. Another class of very rare genetic events, such as uniparental isodisomy, has also been identified in three probands with *ABCA4*-associated retinopathy (Fingert et al., 2006; Khan et al., 2020; Riveiro-Alvarez et al., 2007).
4. It has also been suggested that some of the *ABCA4*-associated retinopathy could be dominant, or di- or polygenic. While theoretically possible, there is currently no evidence for either scenario. While clinically dominant phenotypes, such as those caused by the p.(Gly1961Glu) mutation, are documented in *ABCA4*-associated retinopathy, there is no reason to expect any genetically dominant cases since, based on our current knowledge of the ABCA4 function, a dominant-negative effect is not expected for any *ABCA4* allele. The entire *ABCA4*-associated retinopathy continuum is based on a loss-of-function mechanism, whether complete, or partial; i.e., haploinsufficiency, as in carriers of *ABCA4* variants. Whether the latter mechanism results in a late-onset macular disease is still open for debate (Duncker et al., 2015a; Kjellstrom, 2015; Lee et al., 2019; Maia-Lopes et al., 2008). However, we postulate that, based on pre-mRNA splice assay data (see below), all recessive *ABCA4*-associated retinopathy cases (together from both alleles) have no more total residual ABCA4 activity than 40% (Sangermano et al., 2018, 2019).

## 6. *ABCA4* pre-mRNA splicing defects

### 6.1. In vitro splice assays in HEK293T cells

The analysis of putative splice defects ideally is performed using patient cells in which the gene of interest is endogenously expressed to perform reverse transcription-PCR analysis of the mRNA. In the absence of patient cells or when the gene of interest is not expressed in accessible human tissues, *in vitro* splice assays have traditionally been performed in human cell lines such as human embryonic kidney (HEK293T) cells. To this aim, small genomic fragments (<1 kb) were cloned in splicing vectors. As the *ABCA4* gene is expressed at a very low level in non-ocular human tissues, we also employed minigenes to analyze NCSS variants previously identified in *ABCA4*-associated retinopathy cases. Due to the strong splice sites of vector exons that flank the cloned segments, splicing artefacts were observed (Sangermano et al., 2016). To systematically test the effect of NCSS *ABCA4* variants, we cloned large wild-type genomic fragments (4.0–11.7 kb) of the *ABCA4* gene into a Gateway splicing vector containing *RHO* exons 3 and 5 flanking the region of interest. The resulting splicing constructs were coined ‘midigenes’. Apart from fragments containing parts of two very large introns (introns 6 and 11), all *ABCA4* cloned segments contained at least 3 exons, enabling us to perform RT-PCR using primers annealing to *ABCA4* exons, minimizing the occurrence of artefacts (Sangermano et al., 2018). As the first and last exon do not contain a splice acceptor or donor site, respectively, exon 1, intron 1, intron 49 and exon 50 are not or only partially represented in midigenes.

### 6.2. Causal noncanonical splice site variants in ABCA4

Upon testing all published NCSS variants, 64 showed a wide spectrum of splicing defects, including single exon skipping, multiple exon skipping, exon elongation, intron retention, and partial exon skipping (Table 3) (Bauwens et al., 2019; Fadaie et al., 2019; Khan et al. (2020); Khan et al., 2019; Sangermano et al., 2019; Sangermano et al., 2018; Schulz et al., 2017). The majority of these variants (35/64) resulted in 100% aberrantly spliced RNA and are considered deleterious. Another 12 variants showed >0 and ≤ 30% of normal splice products and can be classified as severe splice variants. Twelve variants showed >30% and ≤70% wild-type RNA and were classified to have a moderate effect, three variants were classified as mild (>70% and ≤80%) and one (C.3608G>A) was classified as benign as 95% of the RNA was correctly spliced. Finally, c.2588G>C resulted in a 3-nt deletion, p.(Gly863del), and a normally splice product that can be translated in ABCA4 protein carrying a missense variant, i.e., p.(Gly863Ala) (Maugeri et al., 1999; Sangermano et al., 2018). In a subsequent study, this variant was found only to be causal (as a mild-moderate allele) when in *cis* with c.5603A>T, p.(Asn1868Ile) (Zernant et al., 2017).

### 6.3. Causal near-exon variants in ABCA4

Seven variants located near exons were found to result in splicing defects that affect neighboring exons (Table 4). Variants c.1937 + 13T>G, c.1937 + 37C>G, c.3191-11T>A and c.4352+61G>A create or strengthen intronic splice sites. They thereby result in 12-nt, 36-nt, 9-nt and 57-nt exon elongations, respectively, leading to nonsense mutations in the corresponding RNA products (c.1937 + 13T>G, c.1937 + 37C>G, c.4352 + 61G>A) or a deletion of one amino acid and the insertion of four amino acids (c.3191-11T>A) (Fadaie et al., 2019; Sangermano et al., 2018). Variants c.161–23T>G and c.4253 + 43G>A result in partial exon 3 and exon 28 skipping, respectively, rendering them mild variants based on genotype/phenotype analyses (Zernant et al., 2018) and *in vitro* splice assays (Bauwens et al., 2019; Sangermano et al., 2019). The c.4253 + 43G>A variant is the most frequent intronic *ABCA4* variant that is not residing in NCSS sequences. Variant c.6148-84A>T resulted in a wildtype and three mutant cDNAs, one of which carried a pseudoexon (PE), one carried a partially overlapping PE and was missing exon 44, and one did not contain exon 45 (Khan et al. (2020)).

### 6.4. Causal deep-intronic variants in ABCA4

The first five causal deep-intronic variants in *ABCA4* were discovered based on the hypothesis that they may strengthen cryptic splice sites flanking PEs that are present in a small fraction of *ABCA4* transcripts within a normal retina (Braun et al., 2013). In this way, small regions of the *ABCA4* locus were sequenced in genetically unsolved *ABCA4*-associated retinopathy cases. Sequencing of the entire *ABCA4* locus (Zernant et al., 2014b) in 114 monoallelic patients revealed another 16 possibly disease-associated variants, followed by variant-specific (Bauwens et al., 2015; Bax et al., 2015; Khan et al., 2019; Schulz et al., 2017; Zernant et al., 2017), or complete locus analysis in several other *ABCA4*-associated retinopathy cohorts (Bauwens et al., 2019; Khan et al. (2020); Sangermano et al., 2019). Finally, functional studies with midigene-based splice assays (Bauwens et al., 2019; Fadaie et al., 2019; Khan et al. (in press); Khan et al., 2019; Sangermano et al., 2019) were used to determine the effect of deep-intronic variants on splicing of 35 deep-intronic variants (Table 4).

**Fig. 7.**
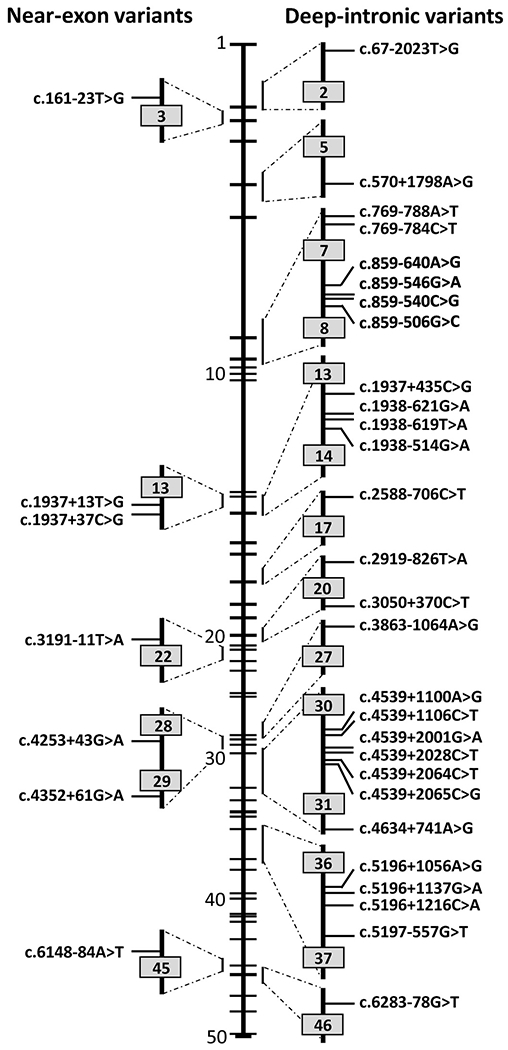
Location of deep-intronic variants in *ABCA4*. Left part shows variants located near exons that result in exon skipping or exon elongation. Right part shows deep-intronic variants that invariably result in the generation of pseudoexons (see Table 4).

*In vitro* splice assays were able to determine a partial or complete picture of the splicing defects for all deep-intronic variants except two. The effects of two neighboring variants in intron 30, i.e. c.4539+2001G>A and c.4539+2028C>T, were only shown using patient-derived photoreceptor progenitor cells (PPCs) (Albert et al., 2018). Both variants do not affect the strength of splice sites flanking a 345-nt PE, but strengthen and/or create exonic splice enhancer motifs inside the pseudo-exon; i.e., have a different disease-causing mechanism than most other deep-intronic pathogenic variants. Based on the *in vitro* splice assays and PPC analysis, 7/35 deep-intronic and near-exon variants had a deleterious effect (i.e. no correct RNA), 13 showed a severe effect, 12 showed a moderate effect, one showed a mild effect, and two (c.1937+435C>G, c.1938-621G>A) were classified as benign as 95% of the RNA was correctly spliced. However, we do consider the latter variants to be causal and attribute the low percentage of mutant transcripts to the cell type used in the splice assay (HEK293T cells). Fig. 7 displays the location of the deep-intronic and near-exon variants. There is a clustering of different deep-intronic variants in introns 7 (n = 4), 13 (n = 4), 30 (n = 6) and 36 (n = 4). The total number of alleles carrying deep-intronic and near-exon variants is 355 (Table 4) (Bauwens et al., 2015; Bauwens et al., 2019; Bax et al., 2015; Braun et al., 2013; Khan et al. (2020); Khan et al., 2019; Nassisi et al., 2019; Sangermano et al., 2019; Schulz et al., 2017; Zernant et al., 2018; Zernant et al., 2014b). Only seven variants were found in more than 10 alleles, i.e. c.4253+43G>A (n = 100), c.4539+2001G>A (n = 64), c.5196+1137G>A (n = 47), c.[769–784C>T;5603A>T] (n = 22), c.4539+2064C>T (n = 27), c.4539+2028C>T (n = 20) and c.5196+1056A>G (n = 22). It is difficult to estimate the frequency of these variants compared to all *ABCA4* variants identified thus far, as not all *ABCA4*-associated retinopathy probands have been analyzed for variants in the entire genomic locus. We estimate that ~5% of all alleles (~10% of probands) carry causal deep-intronic or near-exon variants.

## 7. Structural variants in the *ABCA4* locus

Structural variants (SVs) in the *ABCA4* gene/locus are relatively rare based on Southern blot analysis (Maugeri et al., 1999; Yatsenko et al., 2003), array-comparative genome hybridization (aCGH) assays (Zernant et al., 2014b) and multiplex ligation-dependent probe amplification (MLPA) analysis (Bauwens et al., 2019; Bax et al., 2015; Sangermano et al., 2019; Zernant et al., 2014b). Table 5 lists all 46 reported SVs larger than 20 bp, including 35 deletions, 6 duplications, 2 deletions-insertions, 2 deletions with internal inversions, and 1 insertion. Of the 23 SVs for which the size is known, seven are smaller than 100 bp. However, he predicted effect is severe for all SVs, except for a 7-kb intron 1 duplication, for which the predicted effect is unknown. Only seven SVs have been found in more than one case. Based on nested RT-PCR studies of lymphoblast RNA of a homozygous proband, the intron 28 deletion c.4254–37_4254-15del resulted in the skipping of exons 29 or 28 and 29. The variant was found in homozygosity in 14 cases and in heterozygosity in one case in six families of an Arab-Muslim village in Israel (Beit-Ya’acov et al., 2007). The second most frequent SV is an exon 20–22 deletion that was reported in eight probands originating from Belgium, Germany and the Netherlands (Table 5). Based on these published data, we estimate that 1–2% of *ABCA4*-associated retinopathy probands carry a causal SV.

## 8. Genotype-phenotype correlations

The extensive clinical heterogeneity of *ABCA4*-associated retinopathy is primarily caused by the similarly significant genetic variability in the *ABCA4* locus. Shortly after the discovery of the role of *ABCA4* in various maculopathies, we proposed a somewhat simplistic genotype/phenotype association model suggesting a correlation between the continuum of disease phenotypes and residual ABCA4 activity/function (Cremers et al., 1998; Lewis et al., 1999; van Driel et al., 1998). According to that model, different combinations of “mild”, “moderate”, and “severe” *ABCA4* mutant alleles were suggested to result in distinct phenotypes. While still valid in some, especially extreme, cases, this model underestimates the phenotypic heterogeneity of *ABCA4-*associated retinopathy. The current model is derived from the analysis of large, comprehensively characterized cohorts of patients which have revealed an immensely complex landscape of independent disease trajectories that appear to be unrepresented in the *ABCA4* genotype alone. The combinations of currently known >1200 disease-associated *ABCA4* variants (Allikmets, 2007; Cornells et al., 2017; Maugeri et al., 1999) (www.lovd.nl/ABCA4) explain some, but definitely not all, disease phenotypes (Burke et al., 2012). The population frequency of potentially pathogenic *ABCA4* alleles is 1:20 (Jaakson et al., 2003; Maugeri et al., 1999; Yatsenko et al., 2001), underscoring the substantial impact for the amount of retinal pathology attributable to *ABCA4* variation. Some more recent developments in *ABCA4* genetics, which have allowed us to make more precise genotype/phenotype correlations, are listed below.

### 8.1. Extremely hypomorphic and modifier variants

The emergence of ‘extremely’ hypomorphic and modifier alleles (Bauwens et al., 2019; Runhart et al., 2018; Zernant et al., 2017, 2018), most of which are still unknown, adds another layer of genetic and phenotypic heterogeneity. The most prominent of these variants is p.(Asn1868Ile), with population allele frequencies close to 7% in Europe. It had been shown in many studies to be more frequent in cases vs controls (Aguirre-Lamban et al., 2011; Maugeri et al., 2002; Webster et al., 2001) and speculated (Webster et al., 2001) or suggested (Schulz et al., 2017), that it may be associated with the disease. While most of the association is due to linkage disequilibrium (LD) with highly penetrant pathogenic variants (Schulz et al., 2017; Zernant et al., 2017), we proved the pathogenicity of p.(Asn1868Ile) under a specific condition – it is penetrant when in *trans* from a deleterious *ABCA4* mutation (Zernant et al., 2017). Patients harboring the p.(Asn1868Ile) variant exhibit distinct clinical characteristics, including a very late disease onset with mean age at onset of 36.3 (Zernant et al., 2017) and 41.8 years (Runhart et al., 2018). Affected individuals often show foveal sparing, defined as the structural and function preservation of outer retinal layers in the fovea despite the progressing atrophy of the macula, in ~85% of cases. When the p.(Asn1868Ile) allele resides in cis with other mutations (e.g., c.5461-10T>C or p.(Cys1490Tyr)), the phenotypes are consistent with the overall genotype effect and presented with mostly early onset, severe phenotypes. Interestingly, when in cis with the p.[Gly863Ala,Gly863del] variant as a complex allele, p.(Asn1868Ile) acted as a fully penetrant allele with a mild to moderate effect and resulted in variable phenotypes, reflective of the variant on the opposite allele. Confirming the full penetrance of the complex p.[Gly863Ala,Gly863del;Asn1868Ile] allele, two homozygous patients presented with later onset and milder disease and foveal sparing in one of the two cases (Zernant et al., 2017). The discovery of the p.(Asn1868Ile) pathogenicity when in *trans* with a deleterious *ABCA4* allele solved ~50% of cases who were considered monoallelic at the time and, therefore, explained the missing heritability in ~10% of the entire *ABCA4*-associated retinopathy population (Zernant et al., 2017). These studies also defined the frequent p.[Gly863Ala,Gly863del] variant not as a pathogenic allele on its own, but rather as the first major modifier allele in the *ABCA4* locus.

### 8.2. The c.5882G>A, p.(Gly1961Glu) allele is associated with mild disease and other specific subphenotypes

**Fig. 8.**
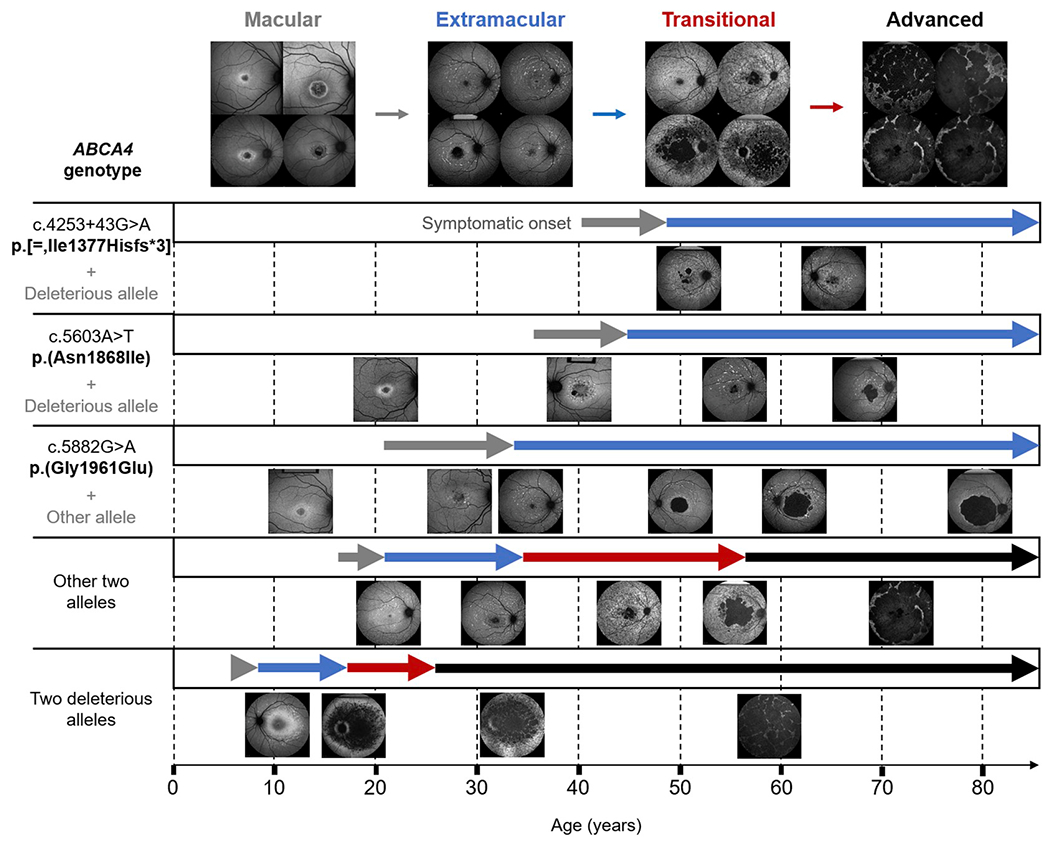
Genotype-phenotype correlations for *ABCA4*-associated retinopathy. Summary of disease trajectories associated variants and genotypes of *ABCA4.* Overall disease severity is defined according to the spatial extent of the disease: Macular stage, disease changes are confined to the central macula; Extramacular stage, disease changes extend beyond the vascular arcades and regions nasal to the optic disc; Transitional stage, disease changes become confluent across the posterior pole initiating peripheral involvement and outer retinal atrophy; Advanced stage, multiple lesions occur and coalesce across the posterior pole. Disease trajectories are defined by the average age at which patients progress through each severity milestone. Three allele-specific trajectories are represented including patients with hypomorphic alleles, c.4253+ 43G> A, p.[ =,Ile1377Hisfs*3] and c.5603A>T, p.(Asn1868Ile) which occur only in *trans* with a deleterious allele, homozygous and compound heterozygous c.5882G> A, p.(Gly1961Glu) alleles, two deleterious alleles and Other two alleles which consist of all other combinations of *ABCA4* alleles. The length of color-coded arrows represents the beginning and duration of defined disease severity stage. Representative autofluorescence images of patients within each trajectory group are arranged according to the age of the depicted phenotype along the time line (For interpretation of the references to color in this figure legend, the reader is referred to the Web version of this article).

Patients harboring the p.(Gly1961Glu) allele in homozygosity or in compound heterozygosity have a substantially different phenotype. Age at onset (mean 22.7 years) is somewhat later than in *ABCA4*-associated retinopathy cases not carrying the p.(Gly1916Glu) or p.(Asn1868Ile) alleles (mean 19.7 years) (Zernant et al., 2017). Patients with the p.(Gly1961Glu) allele exhibit milder disease expression (Burke et al., 2012; Celia et al., 2009), although not in the overall rate of disease progression but in a distinct phenotypic pattern that, interestingly, overlaps with patients harboring the p.(Asn1868Ile) allele. *ABCA4*-associated retinopathy invariably begins as a maculopathy with an enlarging lesion of outer retinal atrophy and accumulation of yellow foci, or flecks, at the level of the RPE. Patients with most other *ABCA4* variants exhibit progressively severe fleck patterns from very few in the macula to a stage of “absolute confluence” across the posterior pole between the ages 30–40 years (Fig. 8).

Patients harboring p.(Gly1961Glu) or p.(Asn1868Ile) consistently exhibit milder spatio-temporal fleck patterns even at advanced age, which never progresses to the absolute confluence stage as illustrated by fundus autofluorescence imaging which detects lipofuscin accumulation (Fig. 8). The resistance to fleck accumulation and lower rate of lipofuscin accumulation seen in bi-allelic cases carrying p.(Gly1961Glu) or p.(Asn1868Ile) (Burke et al., 2014), is further exemplified by the occurrence of a particular phenotype that is seen only in this patient group where a well-defined, unifocal lesion of dark atrophy with proximally-bordering, lesion-centric flecks appears at an early stage of the disease. Similar lesions of chorioretinal atrophy are typically numerous (multifocal) and observed in the background of more advanced fleck stages with most other *ABCA4*-associated retinopathy cases (Fig. 8). Additionally, the individual morphology of flecks in patients with p.(Gly1961Glu) or p.(Asn1868Ile) appears to be distinct in that they are predominantly larger in size and are well defined in shape and generally more sparsely distributed. Of specific interest is the fact that patients with the p.(Gly1961Glu) allele typically present with the ‘bull’s eye maculopathy’ phenotype regardless of the allele in *trans,* thereby acting as ‘clinically dominant’ alleles (Celia et al., 2009). This is especially apparent in multi-generation families, where the other variables (genetic background, age, etc.) are largely the same (Lee et al., 2016). The pathophysiological reasons of this phenomenon remain to be elucidated; however, it is very likely that p.(Gly1961Glu) mutation selectively affects ABCA4 function of foveal cones, often resulting in the ‘optical gap’ phenotype at early disease stages (Noupuu et al., 2014).

### 8.3. Two deleterious ABCA4 alleles result in severe cone-rod dystrophy

Two deleterious alleles in *ABCA4* consistently result in early, childhood-onset of disease symptoms and a rapid progression to advanced disease stages characterized by profound visual impairment and deep, retinal wide degeneration extending towards the equatorial limits of the retina. Such patients have been historically classified as cone-rod dystrophy (MIM# 604116) (Cremers et al., 1998; Maugeri et al., 2000), due to considerable attenuations of cone amplitude on ffERG, or retinitis pigmentosa (RP19, MIM# 601718) (Cremers et al., 1998; Martinez-Mir et al., 1998; Verbakel et al., 2018). Cases of the latter diagnosis indeed exhibit features associated with RP including deposition of bone-spicule pigment, severe retinal vessel attenuation and extinguished ffERG responses, but also atypical features such as early macular involvement. Clinically distinguishing true RP (i.e. rod-cone dystrophy) from severe cone-rod dystrophy at such an advanced stage of disease can be challenging. Distinctions in the extent of residual rod or cone function would not be reliably detectable on ffERG due to widespread degeneration of both systems. Identifying indicators in the ocular history of night blindness and progressive constriction of the visual field may in such cases be more informative. An etiological connection between RP and *ABCA4* thus remains to be established. Alternatively, the commonalities between pathophysiology of deleterious *ABCA4* mutations and RP may result in the manifestation of overlapping features. Other characterizations of biallelic null *ABCA4* phenotypes include the generalized choriocapillaris dystrophy (Bertelsen et al., 2014) and rapid-onset chorioretinopathy (ROC) (Tanaka et al., 2018). ROC is specifically characterized by disease onset within the first decade, a short interval of profoundly increased autofluorescence in the macula followed by the rapid development of degenerative lesions, which enlarge and coalesce across the posterior pole within the third decade of life (Fig. 8).

## 9. Variable expression and penetrance of *ABCA4* alleles

As described above, *ABCA4*-associated retinopathy shows a broad spectrum of clinical expression, with onset ranging from as early as 5 years of age to as late as 70 years. Although genotype-phenotype correlations are apparent, differences in clinical expression between individuals with the same combination of *ABCA4* variants have often been observed, suggesting the involvement of both *cis*- and *trans*-modifier alleles (Lee et al., 2019; Runhart et al., 2018). Recently, the discussion, also between the authors of this review, has focused on late onset cases carrying a deleterious variant on one *ABCA4* allele and the p.(Asn1868Ile) on the other allele.

Researchers at the Nijmegen center calculated the penetrance of the p.(Asn1868Ile) allele, when in *trans* with a deleterious or severe allele, to be ~5% in the general population (Cremers et al., 2018; Runhart et al., 2018) and documented unaffected male patients who carried the same combination of variants as their affected siblings, in three families (Runhart et al., 2018). The time elapsed between the age at onset in the affected siblings and the current age of the unaffected siblings ranged between 14 and 37 years. However, it cannot be excluded that the unaffected bi-allelic individuals could still develop ABCA4-associated retinopathy later in life. In the same study, 22/34 (65%) affected persons were female and 12/34 (35%) were male, which was suggestive (*p* = 0.0615) for a sex imbalance; i.e. it seemed as if late-onset *ABCA4*-associated retinopathy affects more females than males (Cremers et al., 2018; Runhart et al., 2018). In a larger study of 125 international *ABCA4*-associated retinopathy cases carrying p.(Asn1868Ile) in *trans* with a severe or deleterious allele and fully sequenced for variants in the *ABCA4* locus, 79 (63%) were female and 46 (37%) were male. In comparison with a perfect gender balance (50%/50%) observed in 284 bi-allelic *ABCA4*-associated retinopathy probands not carrying presumed hypomorphic alleles, this was statistically significant (*p* = 0.018) (E. Runhart, M. Khan, F.P.M. Cremers, C-M. Dhaenens, unpublished data).

Researchers at Columbia argued against these observations (Allikmets et al., 2018) by suggesting that the disease prevalence (see below in the “Future research” section), and the “strength” of mutations (deleterious vs. severe, etc.) is largely unknown, so one has to be careful with using these variables in statistical calculations. Furthermore, the female-to-male ratio among all 1060 *ABCA4*-associated retinopathy cases at the Columbia center is 54:46 in the entire disease cohort and 57:43 in the sub-cohort with the p.(Asn1868Ile) allele (100 cases) (J. Zernant, W. Lee, R. Allikmets, unpublished data). Excluding the hypomorphic alleles from the entire *ABCA4*-associated retinopathy cohort did not affect the overall 54:46 ratio, i.e. the difference was not statistically significant. Interestingly, the only two “non-penetrant” cases (i.e. older members in families with affected younger cases) with the p.(Asn1868Ile) allele at Columbia are female and carry the p.(Pro1380Lys) allele in *trans*.

The observed differences in the sex data in two large *ABCA4*-associated retinopathy cohorts (with substantial statistical power) suggest further in-depth analysis of this phenomenon across populations. Of additional interest is the fact that differences between the two centers exist in both the overall sex balance in *ABCA4*-associated retinopathy and separately in the hypomorph subgroups.

In summary, these data strongly suggest our incomplete understanding of this, very important, issue, solving of which requires much more data and analysis. Specifically, this part of the *ABCA4* genetic studies requires better understanding of both *cis*- and *trans*-modifier alleles. We cannot exclude that there are thus far missed variants, especially in deep-intronic sequences, on the allele carrying the p.(Asn1868Ile) in *ABCA4*-associated retinopathy cases, which would render these alleles fully penetrant. A primary example for this scenario are the complex alleles harboring both p.(Asn1868Ile) and p.[Gly863Ala,Gly863del] variants, which are fully penetrant (Zernant et al., 2017). However, they would not explain the large differences of expression of *ABCA4*-associated retinopathy within families, in which *trans*-modifiers may play a larger role. Finding these, non-*ABCA4,* modifiers, genetic or non-genetic in nature, which influence the expression of *ABCA4*-associated retinopathy in a subset of the cases, remains an important task.

## 10. Animal models

The first animal model for *ABCA4*-associated retinopathy, the *Abca4* knockout (KO) mouse (*Abca4*^−/−^), was introduced in 1999 (Weng et al., 1999). Since then this mouse, which has also been generated independently by other groups (Kong et al., 2008), has been the main and only animal model for studying *ABCA4*-associated retinopathy. It is obvious that the mouse is not the best animal model for macular disease as it lacks the macula and has very few cones, which are the earliest target of *ABCA4*-associated retinopathy. Therefore, while the *Abca4* KO mouse model does not fully replicate the human condition, its most prominent pathological feature, the extensive accumulation of lipofuscin/A2E, faithfully mimics the disease in humans and allows for precise quantification of the disease status, progression, and the therapeutic effect in pre-clinical studies (Charbel Issa et al., 2013; Sparrow et al., 2013). The rest of the disease features, photoreceptor degeneration (Bok, 2005), delayed dark adaptation (Mata et al., 2001; Weng et al., 1999) and ffERG defects are less robust and not always replicated from study to study.

More recently, two *Abca4* knock-in (KI) animals have been generated and used to study specific protein defects and progression of the disease (Molday et al., 2018; Zhang et al., 2015). In addition to documenting the influence of several *ABCA4* variants on the protein expression, misfolding, trafficking, ATPase activity, etc., these studies also defined the specific *ABCA4* variants, p.(Asn965Ser) and p.[Leu541Pro;Ala1038Val], as mostly deleterious. The resulting phenotype did not differ from the KO mouse, i.e. the protein function was absent regardless of the specific mechanism and the phenotype outcome was the same.

In addition to the KO and KI animals, several other strains have been generated which represent dual or triple KOs, where other genes, in addition to *Abca4,* are knocked out. These include *Abca4*^−/−^/*Rdh8*^−/−^, *Abca4*^−/−^/*Rdh8*^−/−^/*Nrl*^−/−^ and some others. Some of these, especially the *Abca4*^−/−^/*Rdh8*^−/−^ strain, have been extensively used for modeling *ABCA4*-associated retinopathy and even AMD, although they do not, strictly speaking, represent models for these diseases. The *Abca4*^−/−^/*Rdh8*^−/−^ strain presents with much more advanced phenotype, which is not surprising since the two consecutive proteins in the visual cycle are eliminated, thereby causing an earlier onset and fast progressing retinal degeneration (Maeda et al., 2008).

Anatomical differences between the mouse and human retina, namely the absence of a macula due to the spatial distribution of rods and cones in the former has been a long standing challenge in recapitulating disease features found in human patients. As such, extensive efforts over the last 20 years have been devoted to identify a more suitable model system, such as certain breeds of dogs, which have a macula-like “visual streak” that have been efficiently used to study severe retinal degenerations resulting from mutations in the *RPE65* gene (Acland et al., 2001; Jacobson et al., 2005) and among others. While the existence of a dog with *ABCA4*-associated retinopathy was very likely due to the extensive genetic variability not only in the human *ABCA4* locus but also in other mammals, the search was successful only very recently, when when several Labrador retrievers were identified and characterized as a KO for *ABCA4* (Makelainen et al., 2019). The affected animals, homozygous for the deleterious, c.4176insC, p.(Phe1393Leufs*2), allele presented with a phenotype more closely resembling the *ABCA4*-associated retinopathy in humans (Makelainen et al., 2019), including visual impairment at 10 years of age, abnormal fundus images, a complete loss of ABCA4 protein, profound reduction of cone outer segments, and an approximately 50% reduction of photoreceptor nuclei in the affected retinae. The RPE autofluorescence in the affected animal, indicating lipofuscin accumulation, was ~7X higher compared to the unaffected dogs and a clear functional defect was detected by flash-electroretinography (Makelainen et al., 2019). In summary, the dogs with no functional ABCA4 closely resembled the human phenotype, although the disease severity was still less profound than in patients lacking ABCA4 (Tanaka et al., 2018).

## 11. Therapeutic intervention

### 11.1. Clinical trials

Given its relatively high prevalence as well as its disease course, *ABCA4*-associated retinopathy is an attractive target for therapeutic intervention, yet no approved therapy exists. For several reasons, the eye (or retina) is an extremely suitable organ for the development and implementation of novel therapies, i.e. its easy accessibility, compartmentalized and immune-privileged nature, and the possibilities to measure potential therapeutic outcome non-invasively. Like for every other subtype of IRD, the chosen therapeutic strategy for *ABCA4*-associated retinopathy can range from mutation-specific approaches to more generally applicable cell replacement, mainly depending on the primary genetic defect, and the disease stage at the time of treatment (Vazquez-Dominguez et al., 2019). However, in particular for *ABCA4*-associated retinopathy, one of the most challenging aspects of implementing new treatments is the ability to measure therapeutic benefit. Below, we outline the various therapeutic strategies that are currently in clinical trials or in preclinical development, and discuss how disease progression can be accurately and quantitatively measured, with the ultimate goal to define useful clinical outcome parameters to prove therapeutic benefit.

**Fig. 9.**
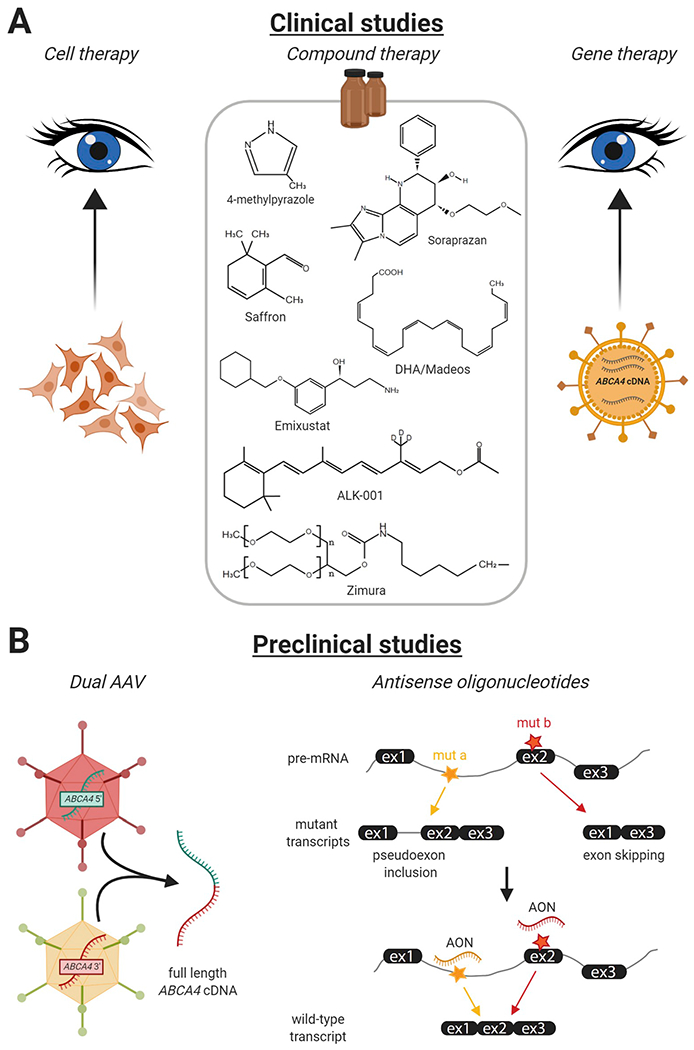
Therapeutic interventions for *ABCA4*-associated retinopathy. (A)Overview of all therapeutic strategies currently in clinical trials; left panel: cell replacement therapy: cells, either in a stem cell state or pre-differentiated *ex vivo* towards a retinal fate are directly injected into the retina; middle panel: structure formulas of compounds currently in clinical trials; right panel: gene augmentation therapy, in which wild-type *ABCA4* cDNA is packaged into a lentiviral vector which is injected into the retina of subjects with ABCA4-associated retinopathy. (B) Examples of therapeutic strategies currently in preclinical development. Left panel: dual AAV-based gene augmentation, in which *ABCA4* cDNA is split into two halves that are aimed to recombine once inside the target cell; right panel: AON-based modulation of pre-mRNA splicing to correct aberrant splicing processes. The figure was created with the aid of BioRender and Illustrator software. Image of the eye was adapted from pixabay.com.

As shown in Table 6, currently there are 16 clinical trials registered at http://clinicaltrials.gov describing therapeutic intervention for *ABCA4*-associated retinopathy (combining the results of search queries ‘ABCA4’ and ‘Stargardt’ and selecting those containing a therapeutic intervention). One additional trial was only registered at https://www.clinicaltrialsregister.eu/. These trials can roughly be divided into three categories, i.e. cell replacement, compound administration and gene augmentation (Fig. 9a).

#### 11.1.1. Cell replacement therapy

In *ABCA4*-associated retinopathy, lipofuscin accumulation exerts a toxic effect causing cell death of neuroretinal and/or RPE cells, mainly in and around the macula. An obvious therapeutic strategy to combat the degeneration of these cells is cell replacement therapy. So far, cell replacement strategies have focused either on the delivery of stem cells, destined to differentiate towards the desired cell once inside the human body, or the delivery of cells that were first differentiated to RPE cells *in vitro* prior to administration. The exact delivery of cells differs per study, including (a combination of) retrobulbar, subtenon, intravitreal, subretinal or intravenous injection of these cells. In addition, the source of the stem cells varies, and includes bone marrow-derived cells as well as human embryonic stem cells (hESCs). The first study was started in 2011, with initial results reported in 2012, demonstrating interim safety and moderate efficacy in a single subject with *ABCA4*-associated retinopathy (Schwartz et al., 2012). Later, results of nine subjects in a dose-escalation study were published, again with no major safety issues, although some complications related to vitreoretinal surgery or immunosuppression were reported. In the majority of subjects, visual function appeared to improve in the treated compared to the contralateral eye (Schwartz et al., 2015). Delivery of hESC-derived RPE cells to the retina of *ABCA4*-associated retinopathy cases also was safe, yet no improvement in visual function could be measured (Mehat et al., 2018). For the other clinical trials aiming to assess the safety and efficacy of cell transplantation, no results have been published yet. In addition to the official clinical trial studies reported at http://www.clinicaltrials.gov, a number of other studies using cell replacement therapy for *ABCA4*-associated retinopathy have been published. In one study, four subjects received a graft of adipose tissue-derived mesenchymal stem cells that was bilaterally delivered between the choroid and the sclera (Oner et al., 2018). Improvements were found in visual performance as well as responses in multifocal ERG analyses, without any safety complications. Another study described the delivery of hESC-derived RPE cells, with safety and moderate efficacy reported up to one year after treatment (Song et al., 2015b). Overall, despite some moderate efficacy reported in a few studies, one should carefully take the pathophysiological mechanism of *ABCA4*-associated retinopathy into consideration when applying cell replacement. With *ABCA4* being (mainly) expressed in photoreceptors, providing RPE cells (or cells destined to become RPE) will likely not have a long-term beneficial effect. Future research should thus be more directed towards the transplantation of cell sheets that contain both RPE and photoreceptor cells. Thus, although in essence, cell replacement therapy may hold great promise for the treatment of *ABCA4*-associated retinopathy, there are many variables that still need to be optimized, including selection of the optimal origin of stem cells, whether or not to differentiate cells *ex vivo* prior to transplantation and if so, until what stage, whether to deliver individual cells or cell sheets, and how to surgically deliver these cells. Finally, also the disease stage of the subject to be treated needs to be taken into account, and can have a major influence on the therapeutic outcome.

#### 11.1.2. Compound administration therapy

An attractive alternative to cell replacement therapy is the administration of compounds, each of which aims to modify either the physiological or the pathological pathways that are affected in *ABCA4*-associated retinopathy. In total, nine of these compounds have been or are currently being tested in human subjects (Table 6). Only for two of these, initial results have been published (MacDonald and Sieving, 2018; Piccardi et al., 2019). In one study, saffron was used, a compound harbouring carotenoid constituents that are able to counteract oxidative stress. Oral administration of either saffron or placebo (in a crossover trial design) to a total of 31 cases with *ABCA4*-associated retinopathy revealed that the supplement was well tolerated but on the short term did not seem to give any measurable improvement in visual function (Piccardi et al., 2019). Long-term studies are needed to investigate the potential therapeutic efficacy of this drug. The other published study also made use of oral delivery of a compound, namely docosahexaenoic acid (DHA), in a cross-over trial design with 11 subjects with *ABCA4*-associated retinopathy. DHA is a major polyunsaturated fatty acid present in high concentrations in the retina (Fliesler and Anderson, 1983) and important for retinal structure and function. It is believed that in *ABCA4*-associated retinopathy cases, DHA metabolism is altered. Overall, the administration of DHA did not lead to visual improvements in this relatively small group. Small adverse events were noted but it was concluded that they were unrelated to the drug (MacDonald and Sieving, 2018). MADEOS is the abbreviation for Macular Degeneration Omega-3 Study in which the efficacy of omega-3 fatty acids is tested in subjects with *ABCA4*-associated retinopathy or AMD. This trial however is still in its recruiting phase.

Another potential drug used for the treatment of *ABCA4*-associated retinopathy is 4-methylpyrazole (4-MP), an alcohol dehydrogenase inhibitor that can delay dark adaptation, at least in laboratory animals. In healthy individuals, intravenous administration did not seem to have an effect on dark adaptation and thus the potential therapeutic efficacy was questioned (Jurgensmeier et al., 2007). However, 4-MP could potentially halt or delay the processing of vitamin A derivatives and thereby prevent the formation of toxic lipofuscin, and was therefore also tested in ten subjects with *ABCA4*-associated retinopathy. No trial results however were published despite the fact that the study was completed more than a decade ago. ALK-001 is also a molecule that aims to prevent the formation of lipofuscin, yet by a slightly different mechanism. In fact, ALK-001 is a deuterated form of vitamin A that is less capable of forming vitamin A dimers, and thereby toxic lipofuscin. The therapeutic potential of ALK-001 was demonstrated in a murine model of *ABCA4*-associated retinopathy, by showing a reduced A2E dimer formation and lipofuscin accumulation compared to age-matched wild-type mice (Charbel Issa et al., 2015). A phase 1 study, in which ALK-001 was administered to 40 healthy adult volunteers, has been completed (NCT02230228), after which the phase II study, where 50 *ABCA4*-associated retinopathy cases were administered with either ALK-001 or placebo at a daily basis, was initiated. No results have been published to date. The last two compounds that are currently being tested in clinical trials both act at the level of the RPE. Emixustat hydrochloride (also known as ACU-4429) is a nonretinoid compound that can exert an inhibitory effect on one of the enzymes involved in the visual cycle, the RPE-specific 65 kDa protein isomerase, encoded by the *RPE65* gene. A phase 1 placebo-controlled study exploring the safety of Emixustat that was administered to healthy individuals at a daily basis (NCT00942240) did not reveal any systemic adverse events, although ocular side effects were observed in the majority of participants (Kubota et al., 2014). However, these effects were considered mild and transient, disappearing after completion of the trial, and warranted further testing in subjects with impaired visual function. Initially, Emixustat was tested in subjects with geographic atrophy associated with dry AMD, with results that are supportive of the anticipated mode of action, yet not having led to spectacular results in terms of visual improvement (Dugel et al., 2015). As illustrated in Table 6, Emixustat is now also being evaluated as a drug for *ABCA4*-associated retinopathy, in two independent trials. The phase 2 trial that aimed to look at the potential short-term benefit has just been completed; however, no results have been reported. The multicentre phase 3 trial in which 162 subjects are planned to be enrolled is currently ongoing, with the aim to monitor the long-term effect of this new drug. One compound that is planned to be studied in human subjects is Zimura, an aptamer that can inhibit the activity of complement factor C5 (Drolet et al., 2016). Zimura was first tested in subjects with age-related macular degeneration but now also for cases with *ABCA4*-associated retinopathy. Finally, a compound called Soraprazan, a fast-acting inhibitor of H^+^,K^+^-ATPase (Simon et al., 2007), is currently being investigated in a multi-national, multicenter, double-masked, placebo-controlled proof of concept trial. Previously, Soraprazan was used for the treatment of patients with gastroesophageal reflux disease. After the discovery that this compound could remove lipofuscin from the RPE in monkeys (Julien and Schraermeyer, 2012), its potential for the treatment of *ABCA4*-associated retinopathy is now also being studied, with no data reported so far.

Together, the various compounds that have been tested so far in subjects with *ABCA4*-associated retinopathy overall can be considered safe, yet none of them succeeded to demonstrate a high therapeutic effect. The advantage of many of these compounds, i.e. the fact that they can be administered orally, also can be considered a disadvantage, since this delivery route does not always allow the active compound to reach a sufficient concentration within the retina. Further research to identify the optimal route of administration, the concentrations needed to exert their effect without causing adverse events, and the ideal treatment regime, is needed to reveal the true therapeutic potential of the aforementioned compounds, as well as those that are currently in preclinical development.

#### 11.1.3. Gene augmentation therapy

The approval of gene augmentation therapy for another subtype of inherited retinal disease that is caused by bi-allelic mutations in *RPE65*, has provided hope for many visually impaired individuals, and is paralleled by the development of similar therapeutic strategies for other genes underlying these disorders (Vazquez-Dominguez et al., 2019). The vast majority of trials assessing gene augmentation for retinal diseases employs adeno-associated viruses (AAVs) to deliver the wild type cDNA of the gene that is mutated. The one and only exception is gene augmentation therapy for *ABCA4* disease, foremost because the size of wild-type *ABCA4* cDNA (6.8 kb) surpasses AAV’s cargo capacity. A phase 1/2 clinical study employing lentiviral delivery of *ABCA4* cDNA (therapeutic molecule SAR422459) has commenced in 2012, but recently has been terminated, without any data of efficacy published. However, in 2016, Parker et al. reported on test-retest variability for a number of clinical outcomes in subjects participating in this trial (Parker et al., 2016). The exact reasons for terminating this trial are unknown; a second trial employing the same therapeutic molecule however is still active and recruiting (Table 6).

Summarizing the clinical trials so far, one can conclude that there are several different treatments for *ABCA4*-associated retinopathy under development, yet none of them so far revealed itself to be the ideal therapeutic intervention. Not only the therapeutic drug itself needs to be further optimized, also the high degree of variability observed in different clinical tests (between and within a subject) warrants an improved trial design. This is further illustrated by the many pre-clinical studies that are currently ongoing, to identify novel therapeutic strategies as well as improved clinical diagnostics, as further outlined below.

### 11.2. Preclinical studies

Besides the clinical studies mentioned above, numerous therapeutic strategies are currently being assessed in preclinical models (examples are illustrated in Fig. 9b). Many of these studies focus on the replacement of stem cell-derived RPE cells, as summarized by Sachdeva et al. (Sachdeva and Eliott, 2016). In addition, novel compounds that e.g. interfere with lipofuscin accumulation, or affect intracellular trafficking of mutant ABCA4 protein are constantly being investigated, a few examples of which are provided below. Administration of BPN-14136, a non-retinoid antagonist of the retinol-binding protein 3 involved in the visual cycle, to *Abca4*^−/−^ mice inhibited bisretinoid synthesis while not altering the rate of the visual cycle, demonstrating its potential for the treatment of *ABCA4*-associated retinopathy (Racz et al., 2018). A systems pharmacological approach identified a number of G-protein coupled receptors that showed improved photoreceptor cell survival and function in *Abca4*^−/−^ mice (Sabirzhanova et al., 2015). VX-809, a molecule previously found to be efficacious for rescuing trafficking of the CFTR protein in cystic fibrosis, also increased the membrane localization of some mutant ABCA4 proteins in cultured HEK293T cells (Liu et al., 2019), although care is warranted when deciding which allele can be amenable for which type of therapy, as the pathogenic mechanism for many variants is still not entirely understood.

As no data on potential efficacy have been reported for lentiviral gene augmentation, alternative strategies to deliver *ABCA4* cDNA to the retinal cells were developed. One delivery strategy uses nanoparticles, whereas others make use of a dual AAV approach. Han and colleagues developed non-viral nanoparticles, and demonstrated that upon sub-retinal delivery in *Abca4*^−/−^ mice, *Abca4* transgene expression persisted up to eight months after injection, and resulted in reduced lipofuscin accumulation in the treated animals (Han et al., 2012). With dual AAV technology, cDNA fragments that exceed the cargo capacity of a single AAV can be split into two halves and each packaged into a separate AAV, with additional sequences that allow reconstitution of the complete cDNA once inside the target cell (Trapani, 2019). Dual AAVs have been used to deliver *ABCA4* cDNA to the (cone-enriched) porcine retina (Trapani et al., 2014, 2015), as well as the retina of *Abca4*^−/−^ mice, with reduced lipofuscin accumulation and/or correction of the autofluorescent phenotype measured in a number of separate studies (Dyka et al., 2019; McClements et al., 2019; Trapani et al., 2015). Although no clinical trials exploring the safety and efficacy of these gene augmentation strategies have been reported, it is expected that these will soon commence. Yet, it is important to realize that the transduction efficiency of both dual AAV vectors and non-viral vectors generally is lower when compared to classical AAV vectors.

Finally, there are also several mutation-specific therapies under development for *ABCA4*-associated retinopathy. These strategies mainly employ antisense oligonucleotides (AONs) and are focussed on those variants that affect pre-mRNA splicing of *ABCA4.* AONs are relatively small and versatile RNA molecules that can be synthesized in such a way that their sequence is complementary to their target pre-mRNA. So far, AONs have mainly been used to block PE inclusions caused by deep-intronic mutations. The first AON described for a retinal disease targets a recurrent deep-intronic mutation in *CEP290,* and was initially tested in lymphoblastoid and fibroblast cells derived from patients homozygously harbouring this mutation (Collin et al., 2012; Gerard et al., 2012). Following further demonstration of efficacy in a humanized mouse model (Garanto et al., 2016) and in iPSC-derived retinal organoids (Dulla et al., 2018; Parfitt et al., 2016), a clinical trial was initiated, with recently reported positive interim results achieved by intravitreal delivery of AONs in subjects with *CEP290*-associated LCA (Cideciyan et al., 2019). As stated in section 6.4, an increasing number of deep-intronic *ABCA4* variants that result in PE inclusion have been identified over the last few years. Using midigene splice assays and patient-derived cells, the ability of AONs to prevents the aberrant PE inclusions caused by several different *ABCA4* variants has been demonstrated (Albert et al., 2018; Bauwens et al., 2019; Garanto et al., 2019; Sangermano et al., 2019), although the therapeutic efficacy so far was only assessed at the RNA level. Besides deep-intronic variants, there are also several *ABCA4* variants reported that are located in or near exons and result in altered pre-mRNA splicing, i.e. exon skipping or exon elongation. Also for these variants, AONs could be employed, e.g. by blocking splice silencer motifs that are created by such variants, or alternative cryptic splice sites that are used. Overall, the versatile nature of AONs render these attractive therapeutic molecules, yet the fact that some of the variants targeted by AONs are rare, or sometimes even ultra-rare, prevent a broad applicability of this therapeutic strategy for a large group of *ABCA4*-associated retinopathy cases. In addition to AON-based splicing correction, genome editing (or RNA editing) approaches are booming, and have the potential to correct mutations regardless of the size of the gene, or the position within the genome. By employing CRISPR/Cas9 technology with the homology-directed repair (HDR) pathway, one can replace e.g. single basepair substitutions with the wild-type nucleotide and in that way repair mutations (Hsu et al., 2014; Yanik et al., 2017). Other recently described strategies include e.g. base pair-editing systems (Billon et al., 2017; Rees and Liu, 2018). It is expected that these strategies will soon also be applied for the mutation-specific correction of *ABCA4* variants.

## 12. Clinical outcome measures

The selection of appropriate clinical outcome measures for *ABCA4*-associated retinopathy should be predicated on a deep understanding of the factors underlying progression. The natural history of *ABCA4*-associated retinopathy consists of multiple trajectories that is largely determined by an individual’s genotype. Effective outcome measures should be generalizable across patients but at the same time, account for their respective differences.

### 12.1. Understanding the parameters of atrophy growth

Monitoring the size of the atrophic lesion has been the primary outcome measure of most *ABCA4*-associated retinopathy clinical trials to date. At face value, atrophy progression appears to be a logical endpoint as it is the most discernible anatomic manifestation of cellular degeneration and historically, the primary endpoint in clinical trials for AMD (Klein et al., 2010; Knudtson et al., 2004; Lindblad et al., 2009). Although convenient, following the AMD model may not be an effective strategy for several reasons: (1) the two conditions share very little pathophysiological and demographic overlap, and (2) studies have already reported extensive differences in their respective rates and spatial correlation with the functional scotoma (Bernstein et al., 2016; Lindner et al., 2017; Sunness and Steiner, 2008). Furthermore, the atrophic process in *ABCA4*-associated retinopathy is difficult to uniformly define, as it is an evolving entity that progressively spans multiple layers of the retina over time across the natural history of the disease (Fig. 1). The sensitivity of SW-AF imaging provides a reasonable assessment of cellular level involvement (Sunness et al., 2006) and as such, the investigators of ProgStar have proposed distinguishing lesions decreased autofluorescence (DDAF) or questionably decreased autofluorescence (QDAF) (ProgStar Report No. 9) (Strauss et al., 2017a, 2017b). Nevertheless, an analysis of reported rates of “DDAF” across the several prominent articles still uncovered a range of variability— 0.94 ± 0.87) mm^2^/year (range 0.2–2.13 mm^2^/year (Chen et al., 2010) 1.58 ± 1.25 (standard deviations) mm^2^/year (range 0.13–5.27 mm^2^/year) (McBain et al., 2012) and 2.5 ± 2.9 mm^2^ (range, 0.02–16.03 mm^2^) (Strauss et al., 2016)— indicating the presence of other unaccounted factors. Fujinami et al. stratified patients according to “AF type”, or the background heterogeneity of flecks, and found that the rate of atrophy enlargement (RAE) (or “DDAF”) in a more severe background exhibit significantly increased rates of growth (Fujinami et al., 2013a). More recent studies have looked to different modes of optical coherence tomography (OCT) to monitor the rate of lesion progression or even the delineation of new lesion types such as “dark atrophy” on OCT angiography (Pellegrini et al., 2016) allowing for a more restrictive definition of atrophy by observable changes in the anatomical layers visible on OCT scans (Arepalli et al., 2018; Cai et al., 2018; Kong et al., 2019; Park et al., 2015; Tanna et al., 2019). Increasing interest is being garnered in this area particularly with advancements in scan resolution, wide-field capture and analytical capabilities such as *en face* analysis (Alabduljalil et al., 2019; Greenstein et al., 2017; Melillo et al., 2016; Sodi et al., 2016).

### 12.2. Targeting the lipofuscin biomarker

Most *ABCA4*-associated retinopathy patients exhibit a spatially homogenous and localized increase in RPE lipofuscin because of ABCA4 dysfunction (Cideciyan et al., 2004). Augmented RPE lipofuscin confers a Vermillion hue to the fundus under white light imaging and can obstruct fluorescence emanating from the underlying choroid in during fluorescein angiograms (FA) giving rise to the distinct “dark” or “silent” choroid in up to 62% of patients (Anmarkrud, 1979; Ernest and Krill, 1966; Fish et al., 1981). Despite the historical utility of FA, its role in the evaluation of STGD1 has been increasingly limited as it is no longer performed on a routine basis in favor of newer, less invasive imaging modalities. Further development of the confocal scanning laser ophthalmoscope (cSLO) introduced various modes of imaging that allow for the capture of an inherent autofluorescence emitted by photoreceptor and RPE fluorophores belonging to the family of bisretinoids that includes N-retinyl-N-retinylidene ethanolamine (A2E), its cis isomers and other related compounds (Fishkin et al., 2005; Kim and Sparrow, 2018; Parish et al., 1998; Wu et al., 2009; Yamamoto et al., 2011). This autofluorescence signal is emitted at wavelengths between 520 and 800 nm and can be captured by the standard SW-AF (448-nm excitation) and the more recently developed ultra-wide field AF (532-nm excitation). The components of photoreceptor/RPE lipofuscin contribute most predominantly to the 488-nm excitation wavelength (SW-AF) where its distribution and pattern in the normal retina has been well described (Delori et al., 1995; Delori et al., 2001; von Ruckmann et al., 1995, 1997). Deviations in SW-AF intensity and texture can be observed in nearly all inherited retinal degenerative diseases where a decrease or absence of SW-AF is generally indicative of a disruption in the tissue architecture in regions not obstructed by retinal vessels or luteal macular pigment in the fovea.

Early attempts at measuring SW-AF (Cideciyan et al., 2004; Lois et al., 2004) lead to the development of quantitative autofluorescence (qAF) by Delori and colleagues (Delori et al., 2011) wherein the SW-AF intensities in non-normalized images (acquired without histogram stretching) were calibrated to the fluorescence intensities of an internal reference mounted within the cSLO and captured simultaneously to compensate for variations in laser power and detector gain. Using this method, Burke et al. verified the increase in autofluorescence and additionally, reported differences amongst genotypes (Burke et al., 2014; Sparrow et al., 2020). In a similar study, the use of qAF was shown to be effective in differentiating *ABCA4*-associated retinopathy from non-*ABCA4*-associated retinopathy (masquerading) bull’s eye maculopathy phenotypes (Duncker et al., 2015b). Near-infrared autofluorescence imaging (NIR-AF), which employs an excitation of signal at wavelength 787 nm, generates a signal corresponding to RPE and choroidal melanin (Keilhauer and Delori, 2006). This has also recently emerged as an effective modality for inherited retinal diseases and *ABCA4*-associated retinopathy (Cideciyan et al., 2007; Cukras et al., 2012; Duncker et al., 2013, 2014; Kellner et al., 2009; Sparrow et al., 2015). Interestingly, Paavo et al. reported quantitative increases in the NIR-AF (787-nm) signal in *ABCA4*-associated retinopathy as well as *Abca4*^−/−^ mice, corroborating results from an earlier study (Charbel Issa et al., 2013) prompting further revisions to either the interpretation of the anatomical origins of the NIR-AF signal or *ABCA4*-associated pathophysiology (e.g. the role of melano-lipofuscin) (Paavo et al., 2018). Additionally, NIR-AF has the practical advantage of being low luminance for ease in acquisition and possible safety consideration.

Effort should be allocated towards tracking other dynamic lipofuscin-related features such as the evolution of flecks patterns and the “leading disease front” (Cideciyan et al., 2015). However, as is the case with qAF, data acquisition and analysis may require a high-level expertise and computational proficiency that can impede its adoption as a routine method in the clinic or treatment trials. Nevertheless, the quantitation of autofluorescence holds promise as an effective outcome measure in *ABCA4*-associated retinopathy, particularly for monitoring the effects of lipofuscin-targeted therapies. Further studies evaluating the longitudinal sensitivity of qAF and a more precise understanding of the cellular origins of autofluorescence may further support its adoption as a primary outcome measure for clinical trials.

### 12.3. Mapping the range of functional loss

Impairment of visual function is the predominant symptom of *ABCA4*-associated retinopathy and all inherited retinal diseases and as such, developing effective ways to track it over time is essential to assessing the disease natural history and response to therapies. The most direct approach is measurement of best-corrected visual acuity (BCVA). Numerous systematic protocols have been developed and this is the standard used by all clinical trials (Table 6). Many studies have documented broad trends in BCVA progression and its relationship to other clinical features in *ABCA4*-associated retinopathy (Birch et al., 2001; Collison and Fishman, 2018; Ergun et al., 2005; Kong et al., 2016; Parodi et al., 2015; Querques et al., 2008; Testa et al., 2012, 2014), however as a method, it is perhaps most susceptible to technical limitations such as measurement bias, repeatability as well as confounders that are specific to *ABCA4*-associated retinopathy such as the variable status of the fovea (Bax et al., 2019b; Collison et al., 2019; Nakao et al., 2012; van Huet et al., 2014) and shifting of the preferred retinal locus (PRL) into consideration (Bethlehem et al., 2014; Greenstein et al., 2008; Krishnan and Bedell, 2018; Schonbach et al., 2017a, 2018).

The full-field electroretinogram (ffERG) is a powerful tool in the diagnostic repertoire of a retinal disease clinic. By measuring the summation of electrical activity generated by cones and rods across the entire retina (separately and combined), its application has been highly effective in broadly classifying severity and predicting the prognosis of individuals with *ABCA4*-associated retinopathy (Lois et al., 2001). The drawback to this generalized approach however, is an insensitivity to subtle changes in the macula, which disproportionately contributes to the aggregate electrophysiological response of the entire retina. The multifocal electroretinogram (mfERG) is more precise as local ERG responses can be recorded simultaneously from many defined regions of the retina. Few studies to date have examined its suitability for monitoring *ABCA4*-associated retinopathy progression (Kuniyoshi et al., 2014; Sisk and Leng, 2014; Tosha et al., 2010), which may be due to the requirement of fixation stability in a disease where most patients have profound central vision loss. The same issue is encountered with conventional methods of static and kinetic perimetry in the mapping of visual fields as they are performed under free-viewing conditions (fixation is not tracked) required but not monitored increasing the likelihood of obtaining false positives (Acton and Greenstein, 2013).

Methods circumventing the fixation requirement include full-field stimulus testing (FST) which provides a psychophysical measure of luminance. Although FST elicits a full-field response, Collison et al. found that cone-mediated thresholds in *ABCA4*-associated retinopathy correlate well with locally defined changes such as visual acuity and macular thickness (Collison et al., 2014). Furthermore, the improvements on FST were instrumental in demonstrating the efficacy of voretigene neparvovec (AAV2-hRPE65v2, Luxturna) in RPE65-associated LCA (NCT00999609) supporting its suitability as an endpoint for aggressive therapies in patients with advanced phenotypes (Jacobson et al., 2009; Maguire et al., 2019). For a more localized assessment, microperimetry (MP) allows for the quantification of mesopic and scotopic visual sensitivity at user-defined points on the retina and fundus tracking which addresses unstable fixation. Studies assessing the scotoma of *ABCA4*-associated retinopathy conclude that it is reliable in tracking longitudinal changes and is highly correlated with changes in BCVA (Cideciyan et al., 2012; Schonbach et al., 2017b).

## 13. Future research

Despite significant advances in deciphering all aspects of *ABCA4*-associated retinopathy, there are still several areas requiring attention in the future. Despite its worldwide occurrence, the prevalence of *ABCA4*-associated retinopathy is not yet known. The 1:8000–10,000 estimate, which everybody cites, comes from the textbook chapter by Blacharski in 1988 (Blacharski, 1988), which states: *“We have seen this condition much more commonly than retinoblastoma, which has been estimated at 1 in 15,000 live births. Fundus flavimaculatus is not as common as retinitis pigmentosa, which has a prevalence of no more than 1 in 5000. We have roughly estimated the incidence to be between 1 in 8000 and 1 in 10,000.”* It is obviously not a scientific way to determine a disease prevalence. The actual prevalence of *ABCA4* -associated retinopathy is very difficult to estimate due to enormous clinical and genetic heterogeneity, variable age of onset, and (still) incomplete genetic data. Most of the genetic information for *ABCA4*-associated retinopathy thus far has been collected from individuals of European descent. To fully appreciate the allelic heterogeneity for *ABCA4*-associated retinopathy and to better understand the differences in genetic background that may influence the expression of this disease, more emphasis should be put on the sequence analysis of *ABCA4*-associated retinopathy cases from non-Caucasian populations.

The wide functional spectrum of *ABCA4* variants, from extremely hypomorphic to deleterious, leaves us with many *ABCA4* variants for which the penetrance is still not known. This has a profound effect on genetic testing and genetic counseling of patients. For example, the disease causality for the p.(Asn1868Ile) allele is still not widely accepted, and this is complicated by the discussion about its penetrance. At least 10% of the entire *ABCA4*-associated retinopathy remains not genetically confirmed if the genetic testing laboratories do not detect and/or report the p.(Asn1868Ile) variant. Considering the p.(Asn1868Ile) allele “benign” has also a detrimental effect on family planning. Screening of large cohorts of familial cases of *ABCA4*-associated retinopathy will continue helping to decipher the penetrance of selected *ABCA4* alleles on a specific genetic background. We estimate that ~10% of *ABCA4*-associated retinopathy probands carries a causal deep-intronic variant and that ~2% of the cases carries a SV. Routine diagnostics therefore cannot only rely on the sequence analysis of the coding regions but also needs to sequence the entire *ABCA4* genomic locus. To establish pathogenicity of novel deep-intronic variants, NCSS variants and rare synonymous coding variants should be tested using *in vitro* splice assays. The accuracy of these assays in the future can be improved by using cell lines that mimic the splicing processes in the normal retina. Another emerging important area is the concept of genetic modifiers in the *ABCA4* locus and in the genome; i.e., both *cis*- and *trans*-modifiers for *ABCA4*-associated retinopathy. We have started to determine some of the *cis*-modifiers (see the example of the p.[Gly863Ala,Gly863del] allele above), but most of these remain obscure. *Trans*-modifiers will be even harder to identify, but with the complete sequencing of the *ABCA4* locus (Bauwens et al., 2019; Sangermano et al., 2019; Zernant et al., 2014b) and the entire exome and genome, these are likely to be found through the analysis of very large familial cohorts, which are, fortunately, possible to obtain even for a relatively rare Mendelian disease. More than 50% of *ABCA4* variants are missense mutations. For frequent missense mutations, we can predict their severity quite accurately based on genotype-phenotype correlations. Most of the rare missense variants however are classified as ‘variants of unknown significance’. If a crystal structure would be determined at high resolution for the ABCA4 protein, proper functional studies could be conducted. Currently, functional studies relying on animal models and *in vitro* data, can reach correct conclusions in some cases; however, in many cases these do not correlate with genetic and clinical studies. One of the best examples is the c.2588G>C, p.[Gly863Ala,Gly863del] variant, which has been shown to result in a dual effect and also in significant defects in *in vitro* assays (Maugeri et al., 1999; Sangermano et al., 2018), but is not penetrant based on clinical/genetic studies unless it is in *cis* with the p.(Asn1868Ile) allele. Determining the high-resolution protein structure would allow investigating the predicted effect of this and many other missense *ABCA4* variants with great precision.

The relatively high prevalence of *ABCA4*-associated retinopathy as well as its progressive nature have led to an enormous attention from academia as well as industry with regard to the development of molecular and cellular therapies. Although *ABCA4* is expressed in photoreceptors, its pathogenic effects are manifest primarily in the RPE (Cideciyan et al., 2004), where accumulation of toxic bisretinoids (Sparrow et al., 2012) occur from the perpetual shedding and subsequent phagocytosis of outer segments (Steinberg et al., 1977; Young, 1967; Young and Bok, 1969). This prevailing disease model, while in many ways consistent with general mechanism of ABCA4 dysfunction, may not be that straightforward. Rod discs are enclosed, self-containing structures and thus bisretinoids generated within would indeed eventually be phagocytosed, along with the rest of the shed outer segment, by RPE. Cone lamellae, however, are contiguous with its plasma membrane and therefore, accumulating bisretinoids may be retained in other cellular compartments, where their toxic effects can be expressed. Consistent with this model is the histopathological observation of lipofuscin-like autofluorescence in the cone inner segments of the retina of a patient with fundus flavimaculatus *(ABCA4*-associated retinopathy) (Birnbach et al., 1994). Clinical imaging studies in patients have largely demonstrated the trend that RPE cell death precedes photoreceptor cell death (Cideciyan et al., 2007; Duncker et al., 2014; Greenstein et al., 2017; Kellner et al., 2009); however most, if not all of these studies, report notable exceptions. For instance, in a study of 24 patients (45 eyes), Duncker et al. reported that zones of RPE atrophy (reduced AF) are statistically larger in NIR-AF compared to SW-AF in the same eye, although a subgroup of younger patients with relatively severe, early-onset disease exhibited larger areas of photoreceptor-attributable EZ loss compared to reduced NIR-AF (Duncker et al., 2014). Similarly, AO-SLO imaging of two STGD1 patients revealed abnormal spacing of rods and cones in otherwise unaffected areas (Song et al., 2015a). Whether photoreceptor cell degeneration precedes or follows RPE loss in *ABCA4*-associated retinopathy remains unresolved. Given the complex role of ABCA4 dysfunction in both cell types, it is likely that the sequence of incapacitation varies in accordance with disease stage, location and overall severity of progression. There are still many challenges ahead, both in terms of identifying and optimizing the right therapeutic drug and finding the right clinical parameters to accurately measure disease progression as well as therapeutic benefit. In the end, there will likely not be a ‘one-strategy-fits-all’ treatment; rather every individual case may need its own tailor-made therapeutic intervention, based on their genetic profile as well as the stage of their disease.

In conclusion, clinical, molecular genetics and therapeutic studies have resulted in a large body of knowledge regarding the fascinating complex *ABCA4*-associated retinopathy. At the same time, new research avenues have opened to unravel the unexplained differences in disease expression between *ABCA4*-associated retinopathy cases, both within and between families. Long-read sequencing technologies will allow phasing of different genomic variants to establish the role of cis-modifiers and enable us to appreciate in great detail normal and abnormal RNA splicing. A full comprehension of the molecular genetic causes and molecular mechanisms of *ABCA4*-associated retinopathy will allow us to develop ‘personalized’ therapies to slow down or stop disease progression.

## Figures and Tables

**Table 1:** Clinical summary of *ABCA4*-associated retinopathy phenocopying genes and their respective phenotypes.

Phenocopy	Gene(s)	Disease	Inheritance	*ABCA4*-associated retinopathy triad			Auxiliary *ABCA4*-associated retinopathy feature	Pathognomonic feature (Non-*ABCA4*-associated retinopathy)
				Macular affection	Flecks	Peripapillary sparing		
Tier 1	*PRPH2*	Pattern macular dystrophy	AD	x	x	x*		
	*ROM1*	Pattern macular dystrophy	AR	x	x	x		
Tier 2	*ABCC6*	Pseudoxanthoma elastieum (PXE)	AR	x	x			Angioid streaks; systemic features
	*ALDH3A2*	Sjögren-Larsson syndrome	AR	x	x			Systemic features
	*BEST1*	Vitelliform macular dystrophy	AR, AD	x	x*		Vitelliform	Multifocal vitelliform lesions in AR disease
	*CDHR1*	Cone-rod dystrophy	AR	x	x*		BEM	
	*CHM*	Choroideremia	XL	x	x*		Chorioretinal atrophy; foveal sparing*	
	*COL4A3, COL4A4, COL4A5*	Alport syndrome	XL*	x	x		BEM	Systemic features
	*CTNNA1*	Butterfly-shaped pigment dystrophy	AD	x	x			
	*ELOVL4*	Stargardt disease 3 (STGD3)	AD	x	x*		BEM	
	*MT-TL1, MT-TK, or MT-TE*	Maternally-inherited diabetes and deafness (MIDD)	Maternal	X	X		Foveal sparing	
	*PROM1*	Stargardt disease 4 (STGD4)	AD	x	x*		BEM	
	*RDH12*	Leber congenital amaurosis (LCA)	AR	x		x	Phenocopies advanced stage STGD1	Variegated watercolour-like pattern of atrophy
	*TIMP3*	Sorsby fundus dystrophy	AD	x	x*		Chorioretinal atrophy	Nummular atrophy
	*ZFYVE26*	Kjellin syndrome	AR	x	x			Systemic features
Tier 3	*C1QTNF5*	Late-onset retinal degeneration (L-ORD)	AD	x			Chorioretinal atrophy	
	*CERKL*	Retinitis pigmentosa with macular involvement	AR	x			Increased AF	
	*CNGA3*	Achromatopsia	AR	x			BEM, optical gap	Severely impaired color distinction; nystagmus
	*CNGB3*	Achromatopsia	AR	x			BEM, optical gap	Severely impaired color distinction; nystagmus
	*CRB1*	Macular dystrophy	AR	x			foveal sparing*	
	*CRX*	Cone-rod dystrophy	AD	x			BEM; foveal sparing	
	*DRAM2*	Cone-rod dystrophy	AR	x			BEM	
	*EFEMP1*	Doyne honeycomb retinal dystrophy; malattia leventinese	AD	x	x*			Drusen (autofluorescent)
	*GUCA1A*	Cone/Cone-rod dystrophy	AD	x			BEM	
	*GUCY2D*	Cone-rod dystrophy	AD, AR	x			BEM	
	*IMPG1*	Vitelliform macular dystrophy	AD	x			Vitelliform	
	*KCNV2*	Cone dystrophy with supernormal rod response (CDSRR)	AR	x			BEM	Supernormal rod response
	*MFSD8*	Macular dystrophy	AR	x			BEM; foveal sparing*	
	*OPN1LW*	Blue cone monochromacy	XL	x			Optical gap	Severely impaired color distinction
	*OPN1MW*	Blue cone monochromacy	XL	x			Optical gap	Severely impaired color distinction
	*PDE6C*	Cone dystrophy	AR	x			Optical gap; BEM	
	*PLA2G5*	Benign fleck	AR		x			Macular sparing
	*POC1B*	Cone-rod dystrophy	AR	x			Optical gap	
	*RDH5*	Fundus albipunetatis	AR		x			Macular sparing
	*RIMS1*	Cone-rod dystrophy	?	x			BEM	
	*RLBP1*	Retinitis punctata albescens	AR		x			Macular sparing
	*RP1L1*	Occult macular dystrophy	AD	x			Optical gap	
	*RPE65*	Retinitis pigmentosa/Leber congenital amaurosis	AR	x			Chorioretinal atrophy	
	*RPGR*	Cone-rod dystrophy	XL	x			BEM; foveal sparing*	Lyonization in female relatives
	*TTLL5*	Cone-rod dystrophy	AR	X			BEM; foveal sparing*	

Phenocopying genes are grouped into three tiers according to their shared phenotypic features with *ABCA4*-associated retinopathy. Auxiliary *ABCA4*-associated retinopathy features include stage-dependent characteristics or phenotypes belonging to clinical or genetic subgroups of *ABCA4*-associated retinopathy. Genes that exhibit the full *ABCA4*-associated retinopathy diagnostic triad represent Tier 1. Genes in Tier 2 exhibit two of the three triad features and Tier 3 consists of genes exhibiting one triad and one auxiliary *ABCA4*-associated retinopathy. Genes within each tier are listed alphabetically.

Asterisks (*) indicate variability in the degree to which the indicated feature overlaps with corresponding feature in *ABCA4*-associated retinopathy. Abbreviations: AD, autosomal dominant; AR, autosomal recessive; XL, X-linked recessive; BEM, bull’s eye maculopathy; AF, autofluorescence.

**Table 2:** *ABCA4* founder variants in various populations.

DNA variant	Protein variant	Population	Allele frequency in *ABCA4*-associated retinopathy (in the founder population)	Allele frequency in the founder population, if known	Reference
c.5882G> A	p.(Gly1961Glu)	Somali	N/A	0.1	Guymer et al. (2001); Burke et al. (2012)
c.[2588G>C;5603A>T]	p.[(Gly863Ala,Gly863del;Asn1868Ile)]	Western Europe	0.15	0.015	Maugeri et al. (1999)
c.768G>T	p.(Leu257Valfs*17)	Dutch	0.08	0.00019	Maugeri et al. (1999); Cremers et al. (2004)
c.[1622T>C;3113C>T]	p.[(Leu541Pro;Ala1038Val)]	German	0.13	0.0003	Rivera et al. (2000)
c.3386G>T	p.(Arg1129Leu)	Spanish	0.24	0.002	Valverde et al. (2006)
C.4539+2001G>A	p.[=,Arg1514Leufs*36]	Belgian	0.025	<0.0001	Bauwens et al. (2015)
c.2894A>G	p.(Asn965Ser)	Danish	0.16	0.0002	Rosenberg et al. (2007)
c.2894A>G	p.(Asn965Ser)	Chinese	0.03	0.0004	Jiang et al. (2016)
c.101_106delCTTTAT	p.(Ser34_Leu35del)	Chinese	0.03	0.0003	Jiang et al. (2016)
c.2424C>G	p.(Tyr808*)	Chinese	0.05	<0.0001	Hu et al. (2019)
c.6320G>A	p.(Arg2107His)	African American	0.19	0.02	Zernant et al. (2014a)
C.2966T>C	p.(Val989Ala)	African American	0.07	0.0025	Zernant et al. (2014a)
c.2971G> C	p.(Gly991Arg)	African American	0.035	0.0064	Zernant et al. (2014a)
c.4139C>T	p.(Pro1380Leu)	Ashkenazi Jewish	0.035	0.002	Sharon et al. (2020)
c.4254–37_4254-15del	p.(Ser1418_Pro1451delinsArg)	Arab-Muslim	0.018	<0.0001	Beit-Ya’acov et al. (2007); Sharon et al. (2020)
c.5318C>T	p.(Ala1773Val)	Mexican	0.17	0.00045	Chacon-Camacho et al. (2013)

Frequency of *ABCA4* variants in *ABCA4*-associated retinopathy cohorts was determined in cited studies. Population frequency in respective populations was determined in the same studies or from gnomAD database. N/A - data not available.

**Table 3:** Noncanonical splice site variants in *ABCA4* and their RNA splice defect assessments in HEK293T cells.

DNA variant	RNA variant	Protein variant	% correct RNA	RNA defect severity	Reference(s)
c.160+5G>C	r.[67_160del,=,161_302delinsl61+1_161+14]	p.[Ile23Alafs*24,=,His55Asnfs*63]	34	Moderate	Sangermano et al. (2018)
c.161G>A	r.[161_302del,=]	p.[Cys54Serfs*14,Cys54Tyr]	44^@^	Moderate	Fadaie et al. (2019)
c.161G>T	r.161_302del	p.(Cys54Serfs*14)	0	Deleterious	Sangermano et al. (2018)
C.302+4A>C	r.161_302del	p.(Cys54Serfs*14)	0	Deleterious	Sangermano et al. (2018)
c.303-3C>G	r.[161_302delins303-2_303–1,302_303ins302-2_302–1]	p.[ Cys54*,Leu102Alafs*14]	0	Deleterious	Sangermano et al. (2018)
c.768G>T	r.768_769ins769+1_769+30	p.(Leu257Valfs*17)	0	Deleterious	Sangermano et al. (2018)
C.859-9T>C	r.[=,859_1356del]	p.[=,Phe287_Arg452del]	76	Mild	Sangermano et al. (2018)
c.1100-6T>A	r.1099_1100ins1099-4_1099-1	p.(Thr367Serfs*6)	0	Deleterious	Sangermano et al. (2018)
c.1554+3A>T	r.[=,1357_1554del]	p.[=,Asp453_Glu518del]	51	Moderate	Khan et al. (2020)
c.1937+5G>A	r.1806_1937del	p.(Tyr603_Ser646del)	0	Deleterious	Fadaie et al. (2019)
c.2161-8G>A	r.2161_2382del	p.(His721_Val794del)	0	Deleterious	Fadaie et al. (2019)
C.2382+5G>C	r.[2161_2382del,=]	p.[His721_Val794del,=]	48	Moderate	Sangermano et al. (2018)
c.2588G>C	r.[2588G>C,2588_2590del]	p.[Gly863Ala,Gly863del]	*60* ^#^	Mild-Moderate	Maugeri et al. (1999); Sangermano et al. (2018)
c.2654-8T>G^#^	r.[2653_2654ins2654-40_2654-1,=]	p.[Gly863Valfs*47,=]	13	Severe	Khan et al. (2020)
c.2919-10T> C	r.[=,2919_3050del]	p.[=,Leu97 3_His1017 delinsPhe]	61	Moderate	Sangermano et al. (2018)
c.2919-6C> A	r.[=,2919_3050del]	p.[=,Leu97 3_His1017 delinsPhe]	80	Mild	Sangermano et al. (2018)
c.3050+5G>A	r.2919_3050del	p.(Leu973_His1017delinsPhe)	0	Deleterious	Sangermano et al. (2018)
c.3191-11T>A	r.3190_3191ins3191-l_3191-9	p.(Gly1064delinsValProProGly)	n.a.	Deleterious^$^	Bauwens et al. (2019)
C.3522+5del	r.[=,3329_3522del]	p.[=,Arg1111 Aspfs*7]	53	Moderate	Sangermano et al. (2018)
c.3607G>A	r.3523_3607del	p.(Thr1176Metfs*2)	11	Severe	Sangermano et al. (2018)
c.3607+3A>T	r.3523_3607del	p.(Thr1176Metfs*2)	0	Deleterious	Sangermano et al. (2018)
c.3608G> A	r.[=,3608_3813del]	p.[Gly1203Glu,Gly1203Aspfs*10]	95	Benign	Khan et al. (2019)
c.3812A> G	r.3608_3813del	p.(Glyl203Aspfs*10)	0	Deleterious	Sangermano et al. (2018)
c.3813G> C	r.3608_3813del	p.(Glyl203Aspfs*10)	0	Deleterious	Sangermano et al. (2018)
c.3862G> A	r.[=3863g>a,3814_3862del]	p.[=,Gly1288Ser,Ile1272Valfs*l01]	69	Moderate	Khan et al. (2020)
C.3862+ 3A> G	r.[=,3814_3862del]	p.[=,Ilel272Valfs*101]	53	Moderate	Sangermano et al. (2018)
c.4128G>A	r.4128_4129ins4128 +1_4128 +12	p.(Gln1376_Ile1377insValLeuLeuSer)	0	Deleterious^$^	Sangermano et al. (2018)
c.4128G>C	r.4128_4129ins4128 +1_4128 +12	p.(Gln1376_Ile1377insValLeuLeuSer)	0	Deleterious^$^	Khan et al. (2019)
c.4129-3C>T	r.[=,3864_4128del,4129_4253del,3864_4253del]	p.[=,Ile1377Hisfs*3,Gly1288Aspfs*45]	76	Mild	Khan et al. (2020)
c.4253+4C>T	r.4129_4253del	p.(Ilel377Hisfs*3)	8	Severe	Sangermano et al. (2018)
c.4253+5G>A	r.4129_4253del	p.(Ilel377Hisfs*3)	0	Deleterious	Sangermano et al. (2018)
c.4253+5G>T	r.4129_4253del	p.(Ilel377Hisfs*3)	5	Severe	Sangermano et al. (2018)
c.4538A>C	r.[4539_4540ins4540+1_4530+30>4467_4539del,4538a>c]	p.[Pro1513_Arg1 514ins10,Cys1490 Glufs*12,Gln1513Pro]	4	Severe	Sangermano et al. (2018)
c.4538A>G	r.[4539_4540ins4540+1_4530+30,4467_4539del]	p.(Arg1513_Arg1514ins10,Cys1490Glufs*12)	0	Deleterious	Sangermano et al. (2018)
c.4539G>A	r.4467_4539del	p.(Cys1490Glufs*12)	5	Severe	Khan et al. (2019)
c.4540-8T>A	r.4539_4540ins4540-6_4540-l	p.(Gln1513insProGln)	0	Deleterious	Khan et al. (2020)
c.4667G> A	r.4635_4667del	p.(Ser1 545_Gln1555del)	0	Deleterious	Fadaie et al. (2019)
c.4667G>C	r.4635_4667del	p.(Ser1545_Gln1555del)	0	Deleterious	Sangermano et al. (2018)
c.4773G>C	r.[4668_5018del,4668_4773del]	p.(Tyr1557_Val1673del,Tyr1557Alafs*18)	0	Deleterious	Sangermano et al. (2018)
c.4773+3A>G	r.[4668_4773del,=]	p.[Tyr1557Alafs*18,=]	25	Severe	Schulz et al. (2017); Sangermano et al. (2018)
c.4773+5G>A	r.[4668_4773del,4668_5018del]	p.[Tyr1557Alafs*18,Tyr1557_Val1673del]	29	Severe	Sangermano et al. (2018)
C.4848+3A>G	r.[4774_4848del,=]	p.[Glyl592_Lysl616del,=]	10	Severe	Khan et al. (2020)
c.4849G>A	r.[4849_5018del,4849_5109del,=]	p.[Vai1617 Alafs*113,Val1617Met,=]	60	Moderate	Khan et al. (2019)
C.5018+5G>A	r.4849_5018del	p.(Vai1617Alafs*113)	0	Deleterious	Fadaie et al. (2019)
C.5196+3_5196+6del	r.4849_5196del	p.(Vall617_Ilel732del)	0	Deleterious	Sangermano et al. (2018)
C.5312+3A>T	r.5197_5312del	p.(Asnl734Glyfs*14)	0	Deleterious	Sangermano et al. (2018)
c.5313-3C> G	r.5312_5313ins5312-2_5312-1	p.(Trpl772Aspfs*7)	0	Deleterious	Sangermano et al. (2018)
c.5460+5G>A	r.5313_5460del	p.(Trp177 2Argfs * 9)	0	Deleterious	Sangermano et al. (2018)
c.5461-10T>C	r.[5461_5714del,5461_5584del]	p.[Thr1821Aspfs*6,Thr1821Valfs*13]	0	Deleterious	Sangermano et al. (2016); Sangermano et al. (2018)
C.5461-6T>G	r.5461_5714del	p.(Thr1821Aspfs*6)	0	Deleterious	Khan et al. (2020)
c.5584G>C	r.5461_5714del	p.(Thr1821Aspfs*6)	0	Deleterious	Sangermano et al. (2018)
c.5584+5G>A	r.[5461_5714del,5461_5584del]	p.[Thr1821Aspfs*6,Thr1821Valfs*13]	0	Deleterious	Sangermano et al. (2018)
c.5584+6T>C	r.[5461_5714del,5461_5584del,5585.5714]	p.[Thr1821Aspfs*6,Thr1821Valfs*13,Glul863Leufs*33]	0	Deleterious	Sangermano et al. (2018)
c.5714 + 5G>A	r.[=,5585_5714del]	p.[=,Glul863Leufs*33]	40	Moderate	Sangermano et al. (2018)
c.5715-5T>G	r.5461_5714delins5715-4_5715-1	p.(Thr1821Serfs*34)	2^^^	Severe	Fadaie et al. (2019)
c.5836-3C>A	r.5835_5836ins5836+1_5836+30	p.(Lysl945Jlel946Pheinsl0)	*0*	Deleterious	Sangermano et al. (2018)
c.5898+5G>A	r.[5898_5899ins_5899 +1_5890-1,5898_5899ins5899+1_5899+170,=]	p.[Cysl967Valfs^4^24,=]	48	Moderate	Khan et al. (2020)
c.5898+5del	r.[5898_5899ins_5899 +1_5890-1,5898_5899ins5899+1_5899+170]	p.(Cysl967Valfs^4^24)	5	Severe	Sangermano et al. (2018)
c.6147G>A	r.6006_6147del	p.(Ser2002Argfs*ll)	0	Deleterious	Fadaie et al. (2019)
c.6385A>G	r.6340_6386del	p.(Val2114Hisfs*4)	0	Deleterious	Fadaie et al. (2019)
C.6386+3A>G	r.[6386_6387ins6386+1_6387-1,6340_6386del,=]	p.[Ser2129Serfs^4^29,Val2114Hisfs*4,=]	26	Severe	Khan et al. (2020)
c.6478A>G	r.[6478a>g,6387_6479del]	p.[Lys2160Glu,Ser2129_Lys2160delinsArg]	55	Moderate	Sangermano et al. (2018)
C.6479+4A>G	r.6387_6479del	p.(Ser2129_Lys2160delinsArg)	0	Deleterious	Sangermano et al. (2018)
C.6729+5_6729+19del	r.6480_6729del	p.(Phe2161 Cysfs*3)	0	Deleterious	Sangermano et al. (2018)

The severity assessment is based on RNA splice defects in HEK293T cells, as follows: 0% correct RNA, deleterious (complete null); >0% and ≤30% correct RNA, severe; >30% and ≤70% correct RNA, moderate; >70% and ≤80% correct RNA, mild; >80% correct RNA, benign. n.a., no quantification shown;

@ The wild-type midigene shows 14% natural 3 exon skipping;

# For variant c.2588G>C, a rough quantification was based on Sanger sequence traces as the two splice products (3-nt difference) could not be separated;

$ Variants with in-frame small amino acid insertions that may not act deleterious in protein function.

^ The wild-type and a mutant (4-nt insertion) fragment co-migrate and together constitute 4% of the total RNA. For variants with multiple effects at the mRNA, the most prevalent product is listed first.

**Table 4:** Causal deep-intronic *ABCA4* variants and their splice defects based on splice assays in HEK293T cells or analysis of patient-derived photoreceptor progenitor cells.

DNA variant	RNA effect	Protein variant	% correct RNA	Severity based on RNA defect	Number of alleles	Reference(s)
C.67-2023T>G	r.[66_67ins67-2266_67-2024,=]	p.[IIe23IIefs*30,=]	33	Moderate	4	Zernant et al. (2014a,b); Khan et al. (2020)
c.161-23T>G	r.[=,161_302del]	p.[=,Cys54Serfs*14]	50	Moderate	2	Bauwens et al. (2019); Khan et al. (2020)
c.570+1798A>G	r.570_571ins570+1733_570+1797	p.(Phel91Leufs*6)	0	Deleterious	3	Zernant et al. (2014a,b); Khan et al. (2020)
c.769–788A>T	r.768_769ins769–778_769-617	p.[Leu257Aspfs*3,=]	4	Severe	1	Khan et al. (2020)
c.769–784C>T	r.[=,768_769ins769-617_769-778]	p.[=,Leu257Aspfs*3]	70	Moderate^@^	22	Bauwens et al. (2019); Khan et al. (2019); Sangermano et al. (2019); Runhart et al. (2019); Khan et al. (2020)
c.859-640A>G	r.858_859ins859-685_859-640	p.(Phe287Tyrfs*69)	0	Deleterious	2	Khan et al. (2020)
c.859-546G>A	r.[858_859ins859-545_859-685,=]	p.[Phe287Tyrfs*33,=]	36	Moderate	1	Khan et al. (2020)
c.859-540C>G	r.858_859ins859-545_859-685	p.(Phe287Tyrfs*33)	0	Deleterious	1	Bauwens et al. (2019)
c.859-506G>C	r.[858_859ins859-503_859-447,=]	p.[Phe287Thrfs*32,=]	24	Severe	6	Sangermano et al. (2019); Khan et al. (2020)
c.1937+13T>G	r.[1937_1938ins_1938+1_1938+12,=]	p.[Phe647*,=]	14	Severe	1	Sangermano et al. (2018)
c.1937+37C>G	r.l937_1938insl938+1_1938+36	p.(Phe647*)	0	Deleterious	2	Khan et al. (2020)
c.1937+435C>G	r.[=,1937_1938ins1937+396_1937+529]	p.[=,Ser646Serfs*25]	91	Benign^#^	4	Sangermano et al. (2019); Khan et al. (2020)
c.1938-621G>A	r.[=,1937_1938insl938-797_1938-624,1937+396_1937+529,1938-797_1938-624]	p.[=,Phe647Alafs*22,Phe647Serfs*22]	93	Benign^^^	1	Khan et al., 2020
c.1938-619A>G	r.1937_1938ins[1938-797_1938-624,1937+396_1937+529,1938-797_1938-624]	p.[Phe647Alafs*22,Phe647Serfs*22]	12	Severe	2	Zernant et al. (2014a,b); Fadaie et al. (2019); Khan et al. (2020)
c.1938-514A>G	r.[1937_1938insl938-623_1938-515,1937+396_1937+529,1938-623_1938-515,=]	p.[Phe647Serfs*155,Phe647Serfs*22,=]	13	Severe	1	Khan et al. (2020)
c.2588-706C>T	r.[2587_2588ins2588-839_2588-708,=]	p.[Gly863Alafs*3,=]	5	Severe	1	Khan et al. (2020)
c.2919-826T>A	r.[2918_2919ins2919-957_2919-825,=]	p.[Leu973Phefs*1,=]	17	Severe	2	Zernant et al. (2014a,b); Fadaie et al. (2019); Khan et al. (2020)
c.3050+370C>T	r.3050_3051ins3050+164_3050+368	p.(Leu1018Glufs*4)	0	Deleterious	2	Zernant et al. (2014a,b); Fadaie et al. (2019); Khan et al. (2020)
c.3863-1064A>G	r.?^%^	p.(?)^%^	70	Moderate	1	Khan et al. (2020)
c.3191-11T> A	r.3190_3191ins3191-1_3191-9	p.(Gly1064delinsValProProGly)	0	Deleterious	1	Bauwens et al. (2019)
c.4253+43G>A	r.[=,4129_4253del]	p.[=,Ile1377Hisfs*3]	64	Moderate	100	Zernant et al. (2018); Sangermano et al. (2019); Bauwens et al. (2019); Khan et al. (2019); Nassisi et al. (2019); Khan et al. (2020)
C.4352+61G>A	r.[4352_4353ins4352+1_4352+57,=]	p.[Glul452*,=]	16	Severe	2	Zernant et al. (2014a,b); Fadaie et al. (2019); Khan et al. (2020)
C.4539+1100A>G	r.[4539_4540ins4539+1033_4539+1100,4539_4540ins4539+989_4539+1100,=]	p.[Arg1514Valfs*31,Arg1514Glyfs*3,=]	19	Severe	2	Sangermano et al. (2019)
C.4539+1106C>T	r.[4539_4540ins4539+1033_4539+1100,4539_4540ins4539+989_4539+1100]	p.[Arg1514Glyfs*3,Arg1514Valfs*31]	3	Severe	4	Bauwens et al. (2019); Khan et al. (2019); Sangermano et al. (2019)
C.4539+2001G>A	r.[=,4539_4540ins4539+1891_4540-2162]	p.[=,Argl514Leufs*36]	50	Moderate^$^	64	Braun et al. (2013); Zernant et al. (2014a,b), Bauwens et al. (2015); Bax et al. (2015); Albert et al. (2018); Sangermano et al. (2019); Bauwens et al. (2019); Khan et al. (2019); Khan et al. (2020)
C.4539+2028C>T	r.[=,4539_4540ins4539+1891_4540-2162]	p.[=,Argl514Leufs*36]	70	Moderate^$^	20	Braun et al. (2013); Zernant et al. (2014a,b); Schulz et al. (2017); Albert et al. (2018); Khan et al. (2019); Khan et al. (2020)
C.4539+2064OT	r.[4539_4540ins4539 +1891_4540-2162,=]	p.[Arg1514Leufs*36,=]	25	Severe	27	Zernant et al. (2014a,b); Bauwens et al. (2019); Khan et al. (2019); Nassisi et al. (2019); Khan et al. (2020)
C.4539+2065C>G	r.[4539_4540ins4539+1891_4539+2060,=]	p.[Argl514Lysfs*35,=]	50	Moderate	1	Khan et al. (2019)
c.4634+741A>G	r.[4634_46354ins4634+614_4634+740,=]	p.[Seri 545Serfs*51,=]	11	Severe	1	Khan et al. (2020)
C.5196+1056A>G	r.5196_5197ins5196+880_5196+1056	p.(Metl733Valfs*2)	2	Severe^&^	22	Braun et al. (2013); Zernant et al. (2014a,b); Schulz et al. (2017); Zernant et al. (2018); Khan et al. (2019); Khan et al. (2020); Khan et al. unpublished
C.5196+1137G>A	r.[=,5196_5197ins5196+1140_5196+1212]	p.[=,Metl733Glufs*78]	55	Moderate^&^	47	Braun et al. (2013); Zernant et al. (2014a,b); Bax et al. (2015); Sangermano et al. (2019); Khan et al. (2019); Nassisi et al. (2019); Khan et al. unpublished
C.5196+1216C>A	r.[=,5196_5197ins5196+1140_5196+1212]	p.[=,Met1733Glufs*78]	33	Moderate^&^	1	Bauwens et al. (2019); Khan et al. unpublished
c.5197-557G>T	r.5196_5197ins5197-563_5197-750	p.(Metl733*)	0	Deleterious	1	Bauwens et al. (2019); Khan et al. unpublished
c.6148-84A>T	r.[6147_6148ins6148-262_6148-90,6006_6147delins6148-310_6148-90,6148_6149del,=]	p.[Val2050Valfs*68,Ile2003Hisfs*30, Val2050_Leu2094del,=]	43	Moderate	1	Khan et al. (2020)
c.6283-78G>T	r.[=,6283_6283ins6283-282_6283-80]	p.[=,Asp2095Aspfs*12]	75	Mild	2	Khan et al. (2020)
				Total:	355	Khan et al. (2020)

Definition of deep-intronic variants: all variants outside the splice site consensus sequences. The severity assessment is based on splice defects observed in transfected HEK293T cells or patient-derived photoreceptor progenitor cells: 0% correct RNA, deleterious (complete null); >0% and ≤30% correct RNA, severe; > 30% and ≤ 70% correct RNA, moderate; > 70% and ≤80% correct RNA, mild; > 80% correct RNA, benign.

@ Based on RT-PCR analysis of patient-derived photoreceptor progenitor cells.

# Variant does not affect splice sites and is presumed to have a more severe effect in the retina.

^ Variant has small effect in HEK293T cells but may have a stronger effect in the retina.

% Due to technical reasons exact boundaries of PE are not determined yet.

$ The RNA splicing defect of these intron 30 variants were analyzed in patient-derived photoreceptor progenitor cells. Based on genotype-phenotype correlations, they are presumed to have a severe (c.4539+2001G>A) and moderate effect (c.4539+2028C>T) on the function of ABCA4.

& Based on midigene *in vitro* splice assays or on RT-PCR analysis of patient-derived photoreceptor progenitor cells (M. Khan et al. unpublished data). For variants with multiple effects at the mRNA, the most prevalent product is listed first.

**Table 5:** Structural variants in *ABCA4*-associated retinopathy patients.

Genomic position (hg 19)	DNA variant	Protein variant	Type of SV	Location	Exact size (if known)	Number of STGD1 cases carrying SV	Reference(s)
94586601_94458796	c.(?,−1),(*1,?)del	p.(?)	del	complete gene	n.a.	1	Valverde et al. (2006)
94586536_94586601	c.(?_−1)_(66+1,67–1)del	p.(?)	del	exon 1	n.a.	1	Khan et al. (2020)
94579011_94586016	c.66+520_67-389dup	p.(?)	dup	intron 1	7006 bp	1	Bauwens et al. (2019)
94553579_94579597	c.67–975_769-4582dup{insA}	p.(Ile23_Val256dup)	dup	intron 1-6	26,019 bp	1	Bauwens et al. (2019)
94568030_94573334	c.442+799_570+541del	p.(Gly148Valfs*89)	del	intron 4-intron 5	5305 bp	3	Lambertus et al. (2015); Bax et al. (2015); Bauwens et al. (2019)
94569917-94562911	c.443–1219,768+ 1439del	p.(Gly148Alafs*23)	del	exons 5-6	7007 bp	1	Khan et al. (2020)
94565348-94561288	c.571–801_768+3062del	p.(Phe191_Val256del)	del	exon 6	4061 bp	1	Khan et al. (2020)
94564419-94564009	c.699_768+341del	p.(Gln234Phefs*5)	del	partial exon 6	411 bp	6	Khan et al. (2020)
94564321_94564376	c.742,768+29del	p.(Val248_Val256del)	del	exon 6	56 bp	1	Riveiro-Alvarez et al. (2013)
94564350_94564547	c.(570+1,571–1)_(768+1,769–1)del	p.(Phe191_Val256del)	del	exon 6	n.a.	2	Khan et al. (2020)
94548997_94548908	c.(768+1,769–1)_(858+1,859–1)del	p.(Leu257_Glu286del)	del	exon 7	n.a.	1	Fujinami et al. (2019)
94546319_94546181	c.859–45_952delinsTCTGACC	p.(?)	del/ins	intron 7-exon	n.a.	1	Fukui et al. (2002)
94534447_94544587	C.1239+291_1555-5574del	p.(Ala414_Glu518del)	del	intron 9-intron 11	10,141 bp	1	Bauwens et al. (2019)
94543443_94520667	c.(1356+1,1357–1)_(2587+1_2588–1)del	p.(Asp453Glufs*38)	del	exon 11-16	n.a.	1	Rozet et al. (1999)
94528873_94528133	c.l555-983_1937+720del	p.(Cys519Phefs*119)	del	exon 12-13	2444 bp	1	Birtel et al. (2018)
94529906-94527518	c.1555–1033,1937+615delinsAGC	p.(Cys519Phefs*119)	del	exon 12-13	2389 bp	1	Khan et al. (2020)
94532364-94526398	c.1555–3491_1938-83delins1734,1761-107inv	p.(Cys519Phefs*22)	del/inv	exon 12-13	5967 bp	1	Khan et al. (2020)
94531301-94522479	c.1555–2428,2161-101delins2160+7,2160+230invATGAATGins	p.(?)	del/inv	exon 12-14	8588 bp	1	Khan et al. (2020)
94528133_94528309	c.(1760+1,1761–1)_(1937+1,1938–1)del	p.(Arg587_Asp645del)	del	exon 13	n.a.	3	Muller et al. (2015); Birtel et al. (2018)
94520667_94520871	c.(2382+1,2383–1)_(2587+1_2588–1)del	p.(Ser795Glufs*38)	del	exon 16	n.a.	1	Khan et al. (2020)
94514513-?	c.(2653+1,2654–1)_(*1_?)del	p.(Gly885Valfs*71)	del	exon 18-50	n.a.	1	Khan et al. (2020)
94514389_94515418	c.2654–905_2743+35del	p.(Gly885_His914del)	del	exon 18	1030 bp	2	Yatsenko et al. (2003)
94510300_94508317	c. 2918 + 775,3328+ 640del	p.(Ser974Glnfs*64)	del	exon 20-22		8	Maugeri et al. (1999); Bax et al. (2015); Lambertus et al. (2015); Muller et al. (2015); Birtel et al. (2018); Bauwens et al. (2019)
94506923_94510186	c.3033_3364del	p.(His1011 Glnfs*53)	del	exon 20-23	n.a.	1	Khan et al. (2020)
94505683_94458796	c.(3522+1,3523–1)_(*l_?)del	p.(Gly1175*)	del	exon 24-50	n.a.	1	Carss et al. (2017)
94497418_94497441	c.4021ins24	p.(?)	ins	exon 27	24 bp	1	Passerini et al. (2010)
94496676_94495001	c.(4128+1,4129–1)_(4539+1_4540–1)del	p.(Ile1377_Gln1513del)	del	exon 28-30	n.a.	1	Khan et al. (2020)
94496096_94496118	c. 4 254-37,4 254-15del	p.(Ser1418_Pro1451delinsArg)	del	intron 28	23 bp, skipping e29 and e28-29	14 hom, 1 het	Beit Ya’acov et al. (2007)
94496279-94487503	c.4254–197,4672delinsGCTTTTT	p.(?)	del	exon 29-33	8770 bp	1	Khan et al. (2020)
94495005_94495030	c.4510_4535del	p.(Glul504Profs*42)	del	exon 30	n.a.	1	Nassisi et al. (2019)
94495187_94486796	c.(4352+1,4353–1)_(5018+1_5019–l)dup	p.(?)	dup	exon 30-35	n.a.	1	Khan et al. (2020)
94480099,94495187	c.4353_5460del	p.(Glul452Argfs*9)	del	exon 30-38	n.a.	1	Khan et al. (2020)
94486911_94486934	c.4880_4903dup	p.(Leu1627_Ala1634dup)	dup	exon 35	24 bp	1	Kellner et al. (2009)
94476941,94461665	c.(5460+ 1,5461 +1)_(6816+1_6817–1)del	p.(Thr1821_Gln2272del)	del	exon 39-49	n.a.	1	Khan et al. (2020)
94457537,94476649	c.5585–166_*1254del	p.(Gly1862Valfs*71)	del	intron 39-3TJTR	19,113 bp	1	Bauwens et al. (2019)
94476485,94458796	c.(5584+1,5585–1)_(*1_?)del	p.(Gly1862_Asp2273delins69)	del	exon 40-50	n.a.	1	Khan et al. (2020)
94471138_94467414	c.(6005+1,6006–1)_(6282+1,6283–1)dup	p.(Asp2095Tyrfs*7)	dup	exon 44-45	n.a.	1	Khan et al. (2020)
94472532-94470240	c.6005+658,6147+757delinsTTTAACAGTGTT	p.(Ser2002Argfs*12)	del	exon 44	2284 bp	1	Khan et al. (2020)
94468246_94463476	c.6148–698_6670del/insTGTGCACCTCCCrAG	p.(?)	del/ins	intron 44-exon 48	n.a.	1	Lee et al. (2016)
94467548_94466392	c.(6147+1,6148–1)_(6479+1,6480–1)del	p.(Val2050Ilefs*21)	del	exon 45-47	n.a.	1	Schorderet et al. (2013)
94467351–94463600	c.6282+63_6546del	p.(Asp2095_Leu2182del)	del	exon 46 to part of exon 48	3752 bp	1	Khan et al. (2020)
94463566_94463601	c.65456580del	p.(Leu2182_Phe2193del)	del	exon 48	36 bp	1	Lewis et al. (1999); Birtel et al. (2018)
94461751-?	c.(6729+1_6730–1)_(*1_?)del	p.(Val2244*)	del	exon 49 to 50	n.a.	1	Khan et al. (in press)
94461722_94461765	c.6730–14_6759del	p.(?)	del	intron 48-exon 49	44 bp	1	Stenirri et al. (2006)
94461716_94461760	c.6730–9_6765dup	p.(His2256_Asp2273delinsTyrLeu)	dup	exon 49	45 bp	1	Jiang et al. (2016)
94458798_94458796	c.(6816+16817–1)_(*1_?)del	p.(Asp2273*)	del	exon 50	n.a.	1	Jespersgaard et al. (2019)

Structural variants reported in PubMed, HGMD (Human Gene Mutation Database) and LOVD (Leiden Open Variation Database). HGVS (Human Genome Variation Society) nomenclature was used. An estimated allele number in STGD1 cases is indicated according to the number of cases described in the publications. SV, structural variant; PMID, PubMed unique identifier number; del, deletion; dup, duplication; ins, insertion; bp, base pair; n.a., not applicable; hom, homozygous; het, heterozygous.

**Table 6:** Registered clinical trials for *ABCA4*-associated retinopathy.

Cell transplantation				
Reg. number	Intervention	Phase	Status	References
NCT01920867	Bone marrow-derived stem cells	n.a.	Enrolling by invitation	
NCT03011541	Bone marrow-derived stem cells	n.a.	Recruiting	
NCT02903576	hESC-derived RPE cells	Phase 1/2	Unknown	
NCT01345006	hESC-derived RPE cells (MA09-hRPE)	Phase 1/2	Completed	Schwartz et al. (2012)
NCT01469832	hESC-derived RPE cells (MA09-hRPE)	Phase 1/2	Completed	Mehat et al. (2018)
NCT03772938	Stem/progenitor cells	Phase 1/2	Enrolling by invitation	
Compound administration				
Reg. number	Intervention	Phase	Status	References
NCT00346853	4-Methylpyrazole (alcohol dehydrogenase inhibitor)	Phase 1	Completed	
NCT02402660	ALK-001 (chemically modified vitamin A)	Phase 2	Recruiting	
NCT00060749	DHA (omega-3 fatty acid)	Phase 1	Completed	MacDonald and Sieving (2018)
NCT03033108	Emixustat (inhibitor of *RPE65*)	Phase 2	Completed	
NCT03772665	Emixustat (inhibitor of *RPE65*)	Phase 3	Recruiting	
NCT03297515	Madeos (omega-3 fatty acid)	n.a.	Recruiting	
NCT01278277	Saffron (neuroprotectant)	Phase 1/2	Unknown	Piccardi et al. (2019)
2018-001496-20	Soraprazan (H^+^,K^+^-ATPase inhibitor)	Phase 2	Active	
NCT03364153	Zimura (inhibitor of complement factor C5)	Phase 2	Active, not recruiting	
Gene augmentation				
Reg. number	Intervention	Phase	Status	References
NCT01367444	SAR422459 (lentiviral delivery *ABCA4* cDNA)	Phase 1/2	Terminated	Parker et al. (2016)
NCT01736592	SAR422459 (lentiviral delivery *ABCA4* cDNA)	Phase 1/2	Active, not recruiting	

Trials are subdivided into three categories. Trials are retrieved from http://www.clinicaltrials.gov and https://www.clinicaltrialsregister.eu/.

## References

[R1] Acland GM, Aguirre GD, Ray J, Zhang Q, Aleman TS, Cideciyan AV, Pearce-Kelling SE, Anand V, Zeng Y, Maguire AM, Jacobson SG, Hauswirth WW, Bennett J, 2001. Gene therapy restores vision in a canine model of childhood blindness. Nat. Genet 28, 92–95. 11326284 10.1038/ng0501-92

[R2] Acton JH, Greenstein VC, 2013. Fundus-driven perimetry (microperimetry) compared to conventional static automated perimetry: similarities, differences, and clinical applications. Can. J. Ophthalmol 48, 358–363. 24093180 10.1016/j.jcjo.2013.03.021 PMC3792399

[R3] Agbaga MP, Brush RS, Mandal MN, Henry K, Elliott MH, Anderson RE, 2008. Role of Stargardt-3 macular dystrophy protein (ELOVL4) in the biosynthesis of very long chain fatty acids. Proc. Natl. Acad. Sci. U. S. A 105, 12843–12848. 18728184 10.1073/pnas.0802607105 PMC2525561

[R4] Aguirre-Lamban J, Gonzalez-Aguilera JJ, Riveiro-Alvarez R, Cantalapiedra D, Avila-Fernandez A, Villaverde-Montero C, Corton M, Bianco-Kelly F, Garcia-Sandoval B, Ayuso C, 2011. Further associations between mutations and polymorphisms in the ABCA4 gene: clinical implication of allelic variants and their role as protector/risk factors. Invest. Ophthalmol. Vis. Sci 52, 6206–6212. 21330655 10.1167/iovs.10-5743

[R5] Ahn J, Molday RS, 2000. Purification and characterization of ABCR from bovine rod outer segments. Methods Enzymol. 315, 864–879. 10736746 10.1016/s0076-6879(00)15887-2

[R6] Alabduljalil T, Patel RC, Alqahtani AA, Gao SS, Gale MJ, Zhang M, Jia Y, Huang D, Chiang PW, Chen R, Wang J, Weleber RG, Pennesi ME, Yang P, 2019. Correlation of outer retinal degeneration and choriocapillaris loss in stargardt disease using en face optical coherence tomography and optical coherence tomography angiography. Am. J. Ophthalmol 202, 79–90. 30771335 10.1016/j.ajo.2019.02.007 PMC6548611

[R7] Albert S, Garanto A, Sangermano R, Khan M, Bax NM, Hoyng CB, Zernant J, Lee W, Allikmets R, Collin RWJ, Cremers FPM, 2018. Identification and rescue of splice defects caused by two neighboring deep-intronic ABCA4 mutations underlying stargardt disease. Am. J. Hum. Genet 102, 517–527. 29526278 10.1016/j.ajhg.2018.02.008 PMC5985352

[R8] Aldahmesh MA, Mohamed JY, Alkuraya HS, Verma IC, Puri RD, Alaiya AA, Rizzo WB, Alkuraya FS, 2011. Recessive mutations in ELOVL4 cause ichthyosis, intellectual disability, and spastic quadriplegia. Am. J. Hum. Genet 89, 745–750. 22100072 10.1016/j.ajhg.2011.10.011 PMC3234380

[R9] Allikmets R, 2000. Further evidence for an association of ABCR alleles with age-related macular degeneration. The International ABCR Screening Consortium. Am. J. Hum. Genet 67, 487–491. 10880298 10.1086/303018 PMC1287193

[R10] Allikmets R, 2007. Stargardt disease: from gene discovery to therapy. In: Tombran-Tink J, Barnstable CJ (Eds.), Biology. Diagnostics and Therapeutics Humana Press, Totowa, NJ, pp. 105–118 Retinal Degenerations.

[R11] Allikmets R, Singh N, Sun H, Shroyer NF, Hutchinson A, Chidambaram A, Gerrard B, Baird L, Stauffer D, Peiffer A, Rattner A, Smallwood P, Li Y, Anderson KL, Lewus RA, Nathans J, Leppert M, Dean M, Lupski JR, 1997. A photoreceptor cell-specific ATP-binding transporter gene (ABCR) is mutated in recessive Stargardt macular dystrophy. Nat. Genet 15, 236–246. 9054934 10.1038/ng0397-236

[R12] Allikmets R, Zernant J, Lee W, 2018. Penetrance of the ABCA4 p.Asn1868Ile allele in stargardt disease. Invest. Ophthalmol. Vis. Sci 59, 5564–5565. 30480703 10.1167/iovs.18-25579 PMC6735614

[R13] Anderson KL, Baird L, Lewds RA, Chinault AC, Otterud B, Leppert M, Lupski JR, 1995. A YAC contig encompassing the recessive Stargardt disease gene (STGD) on chromosome 1p. Am. J. Hum. Genet 57, 1351–1363. 8533764 PMC1801408

[R14] Anmarkrud N, 1979. Fundus fluorescein angiography in fundus flavimaculatus and Stargardts disease. Acta Ophthalmol. 57, 172–182. 452881 10.1111/j.1755-3768.1979.tb00482.x

[R15] Arepalli S, Traboulsi EI, Ehlers JP, 2018. Ellipsoid zone mapping and outer retinal assessment in stargardt disease. Retina 38, 1427–1431. 28613213 10.1097/IAE.0000000000001716 PMC5718981

[R16] Bauwens M, De Zaeytijd J, Weisschuh N, Kohl S, Meire F, Dahan K, Depasse F, De Jaegere S, De Ravel T, De Rademaeker M, Loeys B, Coppieters F, Leroy BP, De Baere E, 2015. An augmented ABCA4 screen targeting noncoding regions reveals a deep intronic founder variant in Belgian Stargardt patients. Hum. Mutat 36, 39–42. 25346251 10.1002/humu.22716

[R17] Bauwens M, Garanto A, Sangermano R, Naessens S, Weisschuh N, De Zaeytijd J, Khan M, Sadler F, Balikova I, Van Cauwenbergh C, Rosseel T, Bauwens J, De Leeneer K, De Jaegere S, Van Laethem T, De Vries M, Carss K, Arno G, Fakin A, Webster AR, de Ravel de l’Argentiere TJL, Sznajer Y, Vuylsteke M, Kohl S, Wissinger B, Cherry T, Collin RWJ, Cremers FPM, Leroy BP, De Baere E, 2019. ABCA4-associated disease as a model for missing heritability in autosomal recessive disorders: novel noncoding splice, cis-regulatory, structural, and recurrent hypomorphic variants. Genet. Med 21, 1761–1771. 30670881 10.1038/s41436-018-0420-y PMC6752479

[R18] Bax NM, Sangermano R, Roosing S, Thiadens AA, Hoefsloot LH, van den Born LI, Phan M, Klevering BJ, Westeneng-van Haaften C, Braun TA, Zonneveld-Vrieling MN, de Wijs I, Mutlu M, Stone EM, den Hollander AI, Klaver CC, Hoyng CB, Cremers FP, 2015. Heterozygous deep-intronic variants and deletions in ABCA4 in persons with retinal dystrophies and one exonic ABCA4 variant. Hum. Mutat 36, 43–47. 25363634 10.1002/humu.22717

[R19] Bax NM, Lambertus S, Cremers FPM, Klevering BJ, Hoyng CB, 2019a. The absence of fundus abnormalities in Stargardt disease. Graefes Arch. Clin. Exp. Ophthalmol 257, 1147–1157. 30903310 10.1007/s00417-019-04280-8

[R20] Bax NM, Valkenburg D, Lambertus S, Klevering BJ, Boon CJF, Holz FG, Cremers FPM, Fleckenstein M, Hoyng CB, Lindner M, for the Foveal Sparing Atrophy Study, 2019b. Foveal sparing in central retinal dystrophies. T. Invest. Ophthalmol. Vis. Sci 60, 3456–3467. 10.1167/iovs.18-26533 31398255

[R21] Beharry S, Zhong M, Molday RS, 2004. N-retinylidene-phosphatidylethanolamine is the preferred retinoid substrate for the photoreceptor-specific ABC transporter ABCA4 (ABCR). J. Biol. Chem 279, 53972–53979. 15471866 10.1074/jbc.M405216200

[R22] Beit-Ya’acov A, Mizrahi-Meissonnier L, Obolensky A, Landau C, Blumenfeld A, Rosenmann A, Banin E, Sharon D, 2007. Homozygosity for a novel ABCA4 founder splicing mutation is associated with progressive and severe Stargardt-like disease. Invest. Ophthalmol. Vis. Sci 48, 4308–4314. 17724221 10.1167/iovs.07-0244

[R23] Bernstein A, Sunness JS, Applegate CA, Tegins EO, 2016. Mapping the dense scotoma and its enlargement in stargardt disease. Retina 36, 1741–1750. 26909568 10.1097/IAE.0000000000001003 PMC4993681

[R24] Bertelsen M, Zernant J, Larsen M, Duno M, Allikmets R, Rosenberg T, 2014. Generalized choriocapillaris dystrophy, a distinct phenotype in the spectrum of ABCA4-associated retinopathies. Invest. Ophthalmol. Vis. Sci 55, 2766–2776. 24713488 10.1167/iovs.13-13391 PMC4005615

[R25] Bethlehem RA, Dumoulin SO, Dalmaijer ES, Smit M, Berendschot TT, Nijboer TC, Van der Stigchel S, 2014. Decreased fixation stability of the preferred retinal location in juvenile macular degeneration. PloS One 9, e100171. 24937090 10.1371/journal.pone.0100171 PMC4061130

[R26] Billon P, Bryant EE, Joseph SA, Nambiar TS, Hayward SB, Rothstein R, Ciccia A, 2017. CRISPR-mediated base editing enables efficient disruption of eukaryotic genes through induction of STOP codons. Mol. Cell. 67, 1068–1079 e1064. 28890334 10.1016/j.molcel.2017.08.008 PMC5610906

[R27] Birch DG, Peters AY, Locke KL, Spencer R, Megarity CF, Travis GH, 2001. Visual function in patients with cone-rod dystrophy (CRD) associated with mutations in the ABCA4(ABCR) gene. Exp. Eye Res 73, 877–886. 11846518 10.1006/exer.2001.1093

[R28] Birnbach CD, Jarvelainen M, Possin DE, Milam AH, 1994. Histopathology and immunocytochemistry of the neurosensory retina in fundus flavimaculatus. Ophthalmology 101, 1211–1219. 8035984 10.1016/s0161-6420(13)31725-4

[R29] Birtel J, Eisenberger T, Gliem M, Muller PL, Herrmann P, Betz C, Zahnleiter D, Neuhaus C, Lenzner S, Holz FG, Mangold E, Bolz HJ, Charbel Issa P, 2018. Clinical and genetic characteristics of 251 consecutive patients with macular and cone/cone-rod dystrophy. Sci. Rep 8, 4824. 29555955 10.1038/s41598-018-22096-0 PMC5859282

[R30] Blacharski P, 1988. Retinal Dystrophies and Degenerations. Raven Press, New York.

[R31] Bok D, 2005. Cellular mechanisms of retinal degenerations: RPE65, ABCA4, RDS, and bicarbonate transporter genes as examples. Retina 25, S18–S20. 16374319 10.1097/00006982-200512001-00007

[R32] Braun TA, Mullins RF, Wagner AH, Andorf JL, Johnston RM, Bakall BB, Deluca AP, Fishman GA, Lam BL, Weleber RG, Cideciyan AV, Jacobson SG, Sheffield VC, Tucker BA, Stone EM, 2013. Non-exomic and synonymous variants in ABCA4 are an important cause of Stargardt disease. Hum. Mol. Genet 22, 5136–5145. 23918662 10.1093/hmg/ddt367 PMC3842174

[R33] Burke TR, Fishman GA, Zernant J, Schubert C, Tsang SH, Smith RT, Ayyagari R, Koenekoop RK, Umfress A, Ciccarelli ML, Baldi A, Iannaccone A, Cremers FP, Klaver CC, Allikmets R, 2012. Retinal phenotypes in patients homozygous for the G1961E mutation in the ABCA4 gene. Invest. Ophthalmol. Vis. Sci 53, 4458–4467. 22661473 10.1167/iovs.11-9166 PMC3394687

[R34] Burke TR, Yzer S, Zernant J, Smith RT, Tsang SH, Allikmets R, 2013. Abnormality in the external limiting membrane in early Stargardt disease. Ophthalmic Genet. 34, 75–77. 22871184 10.3109/13816810.2012.707271 PMC4115808

[R35] Burke TR, Duncker T, Woods RL, Greenberg JP, Zernant J, Tsang SH, Smith RT, Allikmets R, Sparrow JR, Delori FC, 2014. Quantitative fundus autofluorescence in recessive Stargardt disease. Invest. Ophthalmol. Vis. Sci 55, 2841–2852. 24677105 10.1167/iovs.13-13624 PMC4008047

[R36] Cai CX, Light JG, Handa JT, 2018. Quantifying the rate of ellipsoid zone loss in stargardt disease. Am. J. Ophthalmol 186, 1–9. 29126757 10.1016/j.ajo.2017.10.032

[R37] Carss KJ, Arno G, Erwood M, Stephens J, Sanchis-Juan A, Hull S, Megy K, Grozeva D, Dewhurst E, Malka S, Plagnol V, Penkett C, Stirrups K, Rizzo R, Wright G, Josifova D, Bitner-Glindzicz M, Scott RH, Clement E, Allen L, Armstrong R, Brady AF, Carmichael J, Chitre M, Henderson RHH, Hurst J, MacLaren RE, Murphy E, Paterson J, Rosser E, Thompson DA, Wakeling E, Ouwehand WH, Michaelides M, Moore AT, Consortium NI-BRD, Webster AR, Raymond FL, 2017. Comprehensive rare variant analysis via whole-genome sequencing to determine the molecular pathology of inherited retinal disease. Am. J. Hum. Genet 100, 75–90. 28041643 10.1016/j.ajhg.2016.12.003 PMC5223092

[R38] Celia W, Greenstein VC, Zernant-Rajang J, Smith TR, Barile G, Allikmets R, Tsang SH, 2009. G1961E mutant allele in the Stargardt disease gene ABCA4 causes bull’s eye maculopathy. Exp. Eye Res 89, 16–24. 19217903 10.1016/j.exer.2009.02.001 PMC2742677

[R39] Chacon-Camacho OF, Granillo-Alvarez M, Ayala-Ramirez R, Zenteno JC, 2013. ABCA4 mutational spectrum in Mexican patients with Stargardt disease: identification of 12 novel mutations and evidence of a founder effect for the common p.A1773V mutation. Exp. Eye Res 109, 77–82. 23419329 10.1016/j.exer.2013.02.006

[R40] Chapi M, Sabbaghi H, Suri F, Alehabib E, Rahimi-Aliabadi S, Jamali F, Jamshidi J, Emamalizadeh B, Darvish H, Mirrahimi M, Ahmadieh H, Daftarian N, 2019. Incomplete penetrance of CRX gene for autosomal dominant form of cone-rod dystrophy. Ophthalmic Genet. 40, 259–266. 31215831 10.1080/13816810.2019.1622023

[R41] Charbel Issa P, Barnard AR, Singh MS, Carter E, Jiang Z, Radu RA, Schraermeyer U, MacLaren RE, 2013. Fundus autofluorescence in the Abca4(-/-) mouse model of Stargardt disease-correlation with accumulation of A2E, retinal function, and histology. Invest. Ophthalmol. Vis. Sci 54, 5602–5612. 23761084 10.1167/iovs.13-11688 PMC3747716

[R42] Charbel Issa P, Barnard AR, Herrmann P, Washington I, MacLaren RE, 2015. Rescue of the Stargardt phenotype in Abca4 knockout mice through inhibition of vitamin A dimerization. Proc. Natl. Acad. Sci. U. S. A 112, 8415–8420. 26106163 10.1073/pnas.1506960112 PMC4500285

[R43] Chen B, Tosha C, Gorin MB, Nusinowitz S, 2010. Analysis of autofluorescent retinal images and measurement of atrophic lesion growth in Stargardt disease. Exp. Eye Res 91, 143–152. 20398653 10.1016/j.exer.2010.03.021

[R44] Chen L, Lee W, de Carvalho JRL Jr., Chang S, Tsang SH, Allikmets R, Sparrow JR, 2019. Multi-platform imaging in ABCA4-associated disease. Sci. Rep 9, 6436. 31015497 10.1038/s41598-019-42772-z PMC6478712

[R45] Cherry TJ, Yang MG, Harmin DA, Tao P, Timms AE, Bauwens M, Allikmets R, Jones EM, Chen R, De Baere E, Greenberg ME, 2020. Mapping the cfs-regulatory architecture of the human retina reveals noncoding genetic variation in disease. Proc. Natl. Acad. Sci. U. S. A 117, 9001–9012. https://www.pnas.org/content/117/16/9001. 32265282 10.1073/pnas.1922501117 PMC7183164

[R46] Cideciyan AV, Aleman TS, Swider M, Schwartz SB, Steinberg JD, Brucker AJ, Maguire AM, Bennett J, Stone EM, Jacobson SG, 2004. Mutations in ABCA4 result in accumulation of lipofuscin before slowing of the retinoid cycle: a reappraisal of the human disease sequence. Hum. Mol. Genet 13, 525–534. 14709597 10.1093/hmg/ddh048

[R47] Cideciyan AV, Swider M, Aleman TS, Sumaroka A, Schwartz SB, Roman MI, Milam AH, Bennett J, Stone EM, Jacobson SG, 2005. ABCA4-associated retinal degenerations spare structure and function of the human parapapillary retina. Invest. Ophthalmol. Vis. Sci 46, 4739–4746. 16303974 10.1167/iovs.05-0805 PMC2579900

[R48] Cideciyan AV, Swider M, Aleman TS, Roman MI, Sumaroka A, Schwartz SB, Stone EM, Jacobson SG, 2007. Reduced-illuminance autofluorescence imaging in ABCA4-associated retinal degenerations. J Opt Soc Am A Opt Image Sci Vis 24, 1457–1467. 17429493 10.1364/josaa.24.001457 PMC2579898

[R49] Cideciyan AV, Swider M, Aleman TS, Feuer WJ, Schwartz SB, Russell RC, Steinberg JD, Stone EM, Jacobson SG, 2012. Macular function in macular degenerations: repeatability of microperimetry as a potential outcome measure for ABCA4-associated retinopathy trials. Invest. Ophthalmol. Vis. Sci 53, 841–852. 22247458 10.1167/iovs.11-8415 PMC3317423

[R50] Cideciyan AV, Swider M, Schwartz SB, Stone EM, Jacobson SG, 2015. Predicting progression of ABCA4-associated retinal degenerations based on longitudinal measurements of the leading disease front. Invest. Ophthalmol. Vis. Sci 56, 5946–5955. 26377081 10.1167/iovs.15-17698 PMC4572941

[R51] Cideciyan AV, Jacobson SG, Drack AV, Ho AC, Charng J, Garafalo AV, Roman AJ, Sumaroka A, Han IC, Hochstedler MD, Pfeifer WL, Sohn EH, Taiel M, Schwartz MR, Biasutto P, Wit W, Cheetham ME, Adamson P, Rodman DM, Platenburg G, Tome MD, Balikova I, Nerinckx F, Zaeytijd J, Van Cauwenbergh B, Leroy BP, Russell SR, 2019. Effect of an intravitreal antisense oligonucleotide on vision in Leber congenital amaurosis due to a photoreceptor cilium defect. Nat. Med 25, 225–228. 30559420 10.1038/s41591-018-0295-0

[R52] Collin RW, den Hollander AI, van der Velde-Visser SD, Bennicelli J, Bennett J, Cremers FP, 2012. Antisense oligonucleotide (AON)-based therapy for leber congenital amaurosis caused by a frequent mutation in CEP290. Mol. Ther. Nucleic Acids 1, el4. 10.1038/mtna.2012.3 PMC3381589 23343883

[R53] Collison FT, Fishman GA, 2018. Visual acuity in patients with stargardt disease after age 40. Retina 38, 2387–2394. 29068916 10.1097/IAE.0000000000001903

[R54] Collison FT, Fishman GA, McAnany JJ, Zernant J, Allikmets R, 2014. Psychophysical measurement of rod and cone thresholds in stargardt disease with full-field stimuli. Retina 34, 1888–1895. 24695063 10.1097/IAE.0000000000000144 PMC4145077

[R55] Collison FT, Lee W, Fishman GA, Park JC, Zernant J, McAnany JJ, Allikmets R, 2019. Clinical characterization of Stargardt disease patients with the p.N1868I ABCA4 mutation. Retina 39, 2311–2325. 30204727 10.1097/IAE.0000000000002316 PMC6548695

[R56] Cornells SS, Bax NM, Zernant J, Allikmets R, Fritsche LG, den Dunnen JT, Ajmal M, Hoyng CB, Cremers FP, 2017. Silico functional meta-analysis of 5,962 ABCA4 variants in 3,928 retinal dystrophy cases. Hum. Mutat 38, 400–408. 28044389 10.1002/humu.23165

[R57] Cremers FP, van de Pol DJ, van Driel M, den Hollander AI, van Haren FJ, Knoers NV, Tijmes N, Bergen AA, Rohrschneider K, Blankenagel A, Pinckers AJ, Deutman AF, Hoyng CB, 1998. Autosomal recessive retinitis pigmentosa and cone-rod dystrophy caused by splice site mutations in the Stargardťs disease gene ABCR. Hum. Mol. Genet 7, 355–362. 9466990 10.1093/hmg/7.3.355

[R58] Cremers FP, Maugeri A, den Hollander AI, Hoyng CB, 2004. The expanding roles of ABCA4 and CRB1 In inherited blindness. Novartis Found Symp 255,68–79; discussion 79–84, 177–178. 14750597 10.1002/0470092645.ch6

[R59] Cremers FPM, Cornells SS, Runhart EH, Astuti GDN, 2018. Author response: penetrance of the ABCA4 p.Asn1868Ile allele in stargardt disease. Invest. Ophthalmol. Vis. Sci 59, 5566–5568. 30480704 10.1167/iovs.18-25944

[R60] Cukras CA, Wong WT, Caruso R, Cunningham D, Zein W, Sieving PA, 2012. Centrifugal expansion of fundus autofluorescence patterns in Stargardt disease over time. Arch. Ophthalmol 130, 171–179. 21987580 10.1001/archophthalmol.2011.332 PMC3768260

[R61] Dahl SG, Sylte I, Ravna AW, 2004. Structures and models of transporter proteins. J. Pharmacol. Exp. Therapeut 309, 853–860. 10.1124/jpet.103.059972 14988415

[R62] Delori FC, Dorey CK, Staurenghi G, Arend O, Goger DG, Welter JJ, 1995. In vivo fluorescence of the ocular fundus exhibits retinal pigment epithelium lipofuscin characteristics. Invest. Ophthalmol. Vis. Sci 36, 718–729. 7890502

[R63] Delori FC, Goger DG, Dorey CK, 2001. Age-related accumulation and spatial distribution of lipofuscin in RPE of normal subjects. Invest. Ophthalmol. Vis. Sci 42, 1855–1866. 11431454

[R64] Delori F, Greenberg JP, Woods RL, Fischer J, Duncker T, Sparrow J, Smith RT, 2011. Quantitative measurements of autofluorescence with the scanning laser ophthalmoscope. Invest. Ophthalmol. Vis. Sci 52, 9379–9390. 22016060 10.1167/iovs.11-8319 PMC3250263

[R65] Deutman AF, 1971. The hereditary dystrophies of the posterior pole of the eye van Gorcum & Comp. N.V.

[R66] Drolet DW, Green LS, Gold L, Janjic N, 2016. Fit for the eye: aptamers in ocular disorders. Nucleic Acid Therapeut 26, 127–146. 10.1089/nat.2015.0573 PMC4900223 26757406

[R67] Ducroq D, Shalev S, Habib A, Munnich A, Kaplan J, Rozet JM, 2006. Three different ABCA4 mutations in the same large family with several consanguineous loops affected with autosomal recessive cone-rod dystrophy. Eur. J. Hum. Genet 14, 1269–1273. 16896346 10.1038/sj.ejhg.5201691

[R68] Dugel PU, Novack RL, Csaky KG, Richmond PP, Birch DG, Kubota R, 2015. Phase ii, randomized, placebo-controlled, 90-day study of emixustat hydrochloride in geographic atrophy associated with dry age-related macular degeneration. Retina 35, 1173–1183. 25932553 10.1097/IAE.0000000000000606 PMC4452434

[R69] Dulla K, Aguila M, Lane A, Jovanovic K, Parfitt DA, Schulkens I, Chan HL, Schmidt I, Beumer W, Vorthoren L, Collin RWJ, Garanto A, Duijkers L, Brugulat-Panes A, Semo M, Vugler AA, Biasutto P, Adamson P, Cheetham ME, 2018. Splice-modulating oligonucleotide QR-110 restores CEP290 mRNA and function in human c.2991 + 1655A>G LCA10 models. Mol. Ther. Nucleic Acids 12, 730–740. 30114557 10.1016/j.omtn.2018.07.010 PMC6092551

[R70] Duncker T, Lee W, Tsang SH, Greenberg JP, Zernant J, Allikmets R, Sparrow JR, 2013. Distinct characteristics of inferonasal fundus autofluorescence patterns in stargardt disease and retinitis pigmentosa. Invest. Ophthalmol. Vis. Sci 54, 6820–6826. 24071957 10.1167/iovs.13-12895 PMC3799564

[R71] Duncker T, Marsiglia M, Lee W, Zernant J, Tsang SH, Allikmets R, Greenstein VC, Sparrow JR, 2014. Correlations among near-infrared and short-wavelength autofluorescence and spectral-domain optical coherence tomography in recessive Stargardt disease. Invest. Ophthalmol. Vis. Sci 55, 8134–8143. 25342616 10.1167/iovs.14-14848 PMC4266077

[R72] Duncker T, Stein GE, Lee W, Tsang SH, Zernant J, Bearelly S, Hood DC, Greenstein VC, Delori FC, Allikmets R, Sparrow JR, 2015a. Quantitative fundus autofluorescence and optical coherence tomography in ABCA4 carriers. Invest. Ophthalmol. Vis. Sci 56, 7274–7285. 26551331 10.1167/iovs.15-17371 PMC4642605

[R73] Duncker T, Tsang SH, Lee W, Zernant J, Allikmets R, Delori FC, Sparrow JR, 2015b. Quantitative fundus autofluorescence distinguishes ABCA4-associated and non-ABCA4-associated bull’s-eye maculopathy. Ophthalmology 122, 345–355. 25283059 10.1016/j.ophtha.2014.08.017 PMC4306619

[R74] Duncker T, Tsang SH, Woods RL, Lee W, Zernant J, Allikmets R, Delori FC, Sparrow JR, 2015c. Quantitative fundus autofluorescence and optical coherence tomography in PRPH2/RDS- and ABCA4-associated disease exhibiting phenotypic overlap. Invest. Ophthalmol. Vis. Sci 56, 3159–3170. 26024099 10.1167/iovs.14-16343 PMC4451616

[R75] Dyka FM, Molday LL, Chiodo VA, Molday RS, Hauswirth WW, 2019. Dual ABCA4-AAV vector treatment reduces pathogenic retinal A2E accumulation in a mouse model of autosomal recessive stargardt disease. Hum. Gene Ther 30, 1361–1370. 31418294 10.1089/hum.2019.132 PMC6854433

[R76] Eagle RC Jr., Lucier AC, Bernardino VB Jr., Yanoff M, 1980. Retinal pigment epithelial abnormalities in fundus flavimaculatus: a light and electron microscopic study. Ophthalmology 87, 1189–1200. 6165950 10.1016/s0161-6420(80)35106-3

[R77] Ergun E, Hermann B, Wirtitsch M, Unterhuber A, Ko TH, Sattmann H, Scholda C, Fujimoto JG, Stur M, Drexler W, 2005. Assessment of central visual function in Stargardťs disease/fundus flavimaculatus with ultrahigh-resolution optical coherence tomography. Invest. Ophthalmol. Vis. Sci 46, 310–316. 15623790 10.1167/iovs.04-0212

[R78] Ernest JT, Krill AE, 1966. Fluorescein studies in fundus flavimaculatus and drusen. Am. J. Ophthalmol 62, 1–6. 4160788 10.1016/0002-9394(66)91668-0

[R79] Fadaie Z, Khan M, Del Pozo-Valero M, Cornells SS, Ayuso C, Cremers FPM, Roosing S, The ABCA4 Study Group, 2019. Identification of splice defects due to noncanonical splice site or deep-intronic variants in ABCA4. Hum. Mutat 40, 2365–2376. 31397521 10.1002/humu.23890 PMC6899986

[R80] Falfoul Y, Habibi I, Turki A, Chebil A, Hassairi A, Schorderet DF, El Matri L, 2018. Phenotypic progression of stargardt disease in a large consanguineous Tunisian family harboring new ABCA4 mutations. J Ophthalmol 2018, 1030184. 29736279 10.1155/2018/1030184 PMC5875050

[R81] Fingert JH, Eliason DA, Phillips NC, Lotery AJ, Sheffield VC, Stone EM, 2006. Case of Stargardt disease caused by uniparental isodisomy. Arch. Ophthalmol 124, 744–745. 16682602 10.1001/archopht.124.5.744

[R82] Fish G, Grey R, Sehmi KS, Bird AC, 1981. The dark choroid in posterior retinal dystrophies. Br. J. Ophthalmol 65, 359–363. 7248243 10.1136/bjo.65.5.359 PMC1039518

[R83] Fishkin NE, Sparrow JR, Allikmets R, Nakanishi K, 2005. Isolation and characterization of a retinal pigment epithelial cell fluorophore: an all-trans-retinal dimer conjugate. Proc. Natl. Acad. Sci. U. S. A 102, 7091–7096. 15870200 10.1073/pnas.0501266102 PMC1129110

[R84] Fishman GA, 1976. Fundus flavimaculatus. A clinical classification. Arch. Ophthalmol 94, 2061–2067. 999551 10.1001/archopht.1976.03910040721003

[R85] Fishman GA, Stone EM, Grover S, Derlacki DJ, Haines HL, Hockey RR, 1999. Variation of clinical expression in patients with Stargardt dystrophy and sequence variations in the ABCR gene. Arch. Ophthalmol 117, 504–510. 10206579 10.1001/archopht.117.4.504

[R86] Fliesler SJ, Anderson RE, 1983. Chemistry and metabolism of lipids in the vertebrate retina. Prog. Lipid Res 22, 79–131. 6348799 10.1016/0163-7827(83)90004-8

[R87] Franceschetti A, 1965. A special form of tapetoretinal degeneration: fundus flavimaculatus. Trans. Am. Acad. Ophthalmol. Otolaryngol 69, 1048–1053. 5861644

[R88] Franceschetti A, François J, 1965. [Fundus flavimaculatus]. Arch. Ophtalmol. Rev Gen. Ophtalmol 25, 505–530. 4221555

[R89] François P, Turut P, Puech B, Hache JC, 1975. [Stargardťs disease and fundus flavimaculatus]. Arch. Ophtalmol. Rev Gen. Ophtalmol 35, 817–846. 131535

[R90] Fujinami K, Lois N, Mukherjee R, McBain VA, Tsunoda K, Tsubota K, Stone EM, Fitzke FW, Bunce C, Moore AT, Webster AR, Michaelides M, 2013a. A longitudinal study of Stargardt disease: quantitative assessment of fundus autofluorescence, progression, and genotype correlations. Invest. Ophthalmol. Vis. Sci 54, 8181–8190. 24265018 10.1167/iovs.13-12104

[R91] Fujinami K, Sergouniotis PI, Davidson AE, Wright G, Chana RK, Tsunoda K, Tsubota K, Egan CA, Robson AG, Moore AT, Holder GE, Michaelides M, Webster AR, 2013b. Clinical and molecular analysis of Stargardt disease with preserved foveal structure and function. Am. J. Ophthalmol 156, 487–501 e481. 23953153 10.1016/j.ajo.2013.05.003

[R92] Fujinami K, Zernant J, Chana RK, Wright GA, Tsunoda K, Ozawa Y, Tsubota K, Robson AG, Holder GE, Allikmets R, Michaelides M, Moore AT, 2015. Clinical and molecular characteristics of childhood-onset Stargardt disease. Ophthalmology 122, 326–334. 25312043 10.1016/j.ophtha.2014.08.012 PMC4459618

[R93] Fujinami K, Strauss RW, Chiang JP, Audo IS, Bernstein PS, Birch DG, Bomotti SM, Cideciyan AV, Ervin AM, Marino MJ, Sahel JA, Mohand-Said S, Sunness JS, Traboulsi EI, West S, Wojciechowski R, Zrenner E, Michaelides M, Scholl HPN, ProgStar Study G, ProgStar Study G, 2019. Detailed genetic characteristics of an international large cohort of patients with Stargardt disease: ProgStar study report 8. Br. J. Ophthalmol 103, 390–397. 29925512 10.1136/bjophthalmol-2018-312064 PMC6579578

[R94] Fukui T, Yamamoto S, Nakano K, Tsujikawa M, Morimura H, Nishida K, Ohguro N, Fujikado T, Irifune M, Kuniyoshi K, Okada AA, Hirakata A, Miyake Y, Tano Y, 2002. ABCA4 gene mutations in Japanese patients with Stargardt disease and retinitis pigmentosa. Invest. Ophthalmol. Vis. Sci 43, 2819–2824. 12202497

[R95] Garanto A, Chung DC, Duijkers L, Corral-Serrano JC, Messchaert M, Xiao R, Bennett J, Vandenberghe LH, Collin RW, 2016. In vitro and in vivo rescue of aberrant splicing in CEP290-associated LCA by antisense oligonucleotide delivery. Hum. Mol. Genet 25, 2552–2563. 27106101 10.1093/hmg/ddw118 PMC6086559

[R96] Garanto A, Duijkers L, Tomkiewicz TZ, Collin RWJ, 2019. Antisense Oligonucleotide Screening to Optimize the Rescue of the Splicing Defect Caused by the Recurrent Deep-intronic ABCA4 Variant C.4539 + 2001 G> A in Stargardt Disease. Genes, Basel, pp. 10. 10.3390/genes10060452 PMC6628380 31197102

[R97] Garg A, Lee W, Sengillo JD, Allikmets R, Garg K, Tsang SH, 2017. Peripapillary sparing in RDH12-associated Leber congenital amaurosis. Ophthalmic Genet 38, 575–579. 28513254 10.1080/13816810.2017.1323339 PMC6314194

[R98] Gerard X, Perrault I, Hanein S, Silva E, Bigot K, Defoort-Delhemmes S, Rio M, Munnich A, Scherman D, Kaplan J, Kichler A, Rozet JM, 2012. AON-mediated exon skipping restores ciliation in fibroblasts harboring the common leber congenital amaurosis CEP290 mutation. Mol. Ther. Nucleic Acids 1, e29. 23344081 10.1038/mtna.2012.21 PMC3390222

[R99] Gerber S, Rozet JM, Bonneau D, Souied E, Camuzat A, Dufier JL, Amalric P, Weissenbach J, Munnich A, Kaplan J, 1995. A gene for late-onset fundus flavimaculatus with macular dystrophy maps to chromosome 1p13. Am. J. Hum. Genet 56, 396–399. 7847373 PMC1801138

[R100] Giroux JM, Barbeau A, 1972. Erythrokeratodermia with ataxia. Arch. Dermatol 106, 183–188. 5048218

[R101] Greenstein VC, Santos RA, Tsang SH, Smith RT, Barile GR, Seiple W, 2008. Preferred retinal locus in macular disease: characteristics and clinical implications. Retina 28, 1234–1240. 18628727 10.1097/IAE.0b013e31817c1b47 PMC2749558

[R102] Greenstein VC, Nunez J, Lee W, Schuerch K, Fortune B, Tsang SH, Allikmets R, Sparrow JR, Hood DC, 2017. A comparison of en face optical coherence tomography and fundus autofluorescence in stargardt disease. Invest. Ophthalmol. Vis. Sci 58, 5227–5236. 29049723 10.1167/iovs.17-22532 PMC5642378

[R103] Guymer RH, Heon E, Lotery AJ, Munier FL, Schorderet DF, Baird PN, McNeil RJ, Haines H, Sheffield VC, Stone EM, 2001. Variation of codons 1961 and 2177 of the Stargardt disease gene is not associated with age-related macular degeneration. Arch. Ophthalmol 119, 745–751. 11346402 10.1001/archopht.119.5.745

[R104] Westeneng-van Haaften SC, Boon CJ, Cremers FP, Hoefsloot LH, den Hollander AI, Hoyng CB, 2012. Clinical and genetic characteristics of late-onset Stargardťs disease. Ophthalmology 119, 1199–1210. 22449572 10.1016/j.ophtha.2012.01.005

[R105] Han Z, Conley SM, Makkia RS, Cooper MJ, Naash MI, 2012. DNA nanoparticle-mediated ABCA4 delivery rescues Stargardt dystrophy in mice. J. Clin. Invest 122, 3221–3226. 22886305 10.1172/JCI64833 PMC3428101

[R106] Hsu PD, Lander ES, Zhang F, 2014. Development and applications of CRISPR-Cas9 for genome engineering. Cell 157, 1262–1278. 24906146 10.1016/j.cell.2014.05.010 PMC4343198

[R107] Hu FY, Li JK, Gao FJ, Qi YH, Xu P, Zhang YJ, Wang DD, Wang LS, Li W, Wang M, Chen F, Shen SM, Xu GZ, Zhang SH, Chang Q, Wu JH, 2019. ABCA4 gene screening in a Chinese cohort with stargardt disease: identification of 37 novel variants. Front. Genet 10, 773. 31543898 10.3389/fgene.2019.00773 PMC6739639

[R108] Huckfeldt RM, East JS, Stone EM, Sohn EH, 2016. Phenotypic variation in a family with pseudodominant stargardt disease. JAMA Ophthalmol 134, 580–583. 27030965 10.1001/jamaophthalmol.2015.5471

[R109] Jaakson K, Zernant J, Kulm M, Hutchinson A, Tonisson N, Glavac D, Ravnik-Glavac M, Hawlina M, Meltzer MR, Caruso RC, Testa F, Maugeri A, Hoyng CB, Gouras P, Simonelli F, Lewis RA, Lupski JR, Cremers FP, Allikmets R, 2003. Genotyping microarray (gene chip) for the ABCR (ABCA4) gene. Hum. Mutat 22, 395–403. 14517951 10.1002/humu.10263

[R110] Jacobson SG, Aleman TS, Cideciyan AV, Sumaroka A, Schwartz SB, Windsor EA, Traboulsi EI, Heon E, Pittler SJ, Milam AH, Maguire AM, Palczewski K, Stone EM, Bennett J, 2005. Identifying photoreceptors in blind eyes caused by RPE65 mutations: prerequisite for human gene therapy success. Proc. Natl. Acad. Sci. U. S. A 102, 6177–6182. 15837919 10.1073/pnas.0500646102 PMC1087926

[R111] Jacobson SG, Aleman TS, Cideciyan AV, Roman AJ, Sumaroka A, Windsor EA, Schwartz SB, Heon E, Stone EM, 2009. Defining the residual vision in leber congenital amaurosis caused by RPE65 mutations. Invest. Ophthalmol. Vis. Sci 50, 2368–2375. 19117922 10.1167/iovs.08-2696 PMC2731629

[R112] Jespersgaard C, Fang M, Bertelsen M, Dang X, Jensen H, Chen Y, Bech N, Dai L, Rosenberg T, Zhang J, Moller LB, Tumer Z, Brondum-Nielsen K, Gronskov K, 2019. Molecular genetic analysis using targeted NGS analysis of 677 individuals with retinal dystrophy. Sci. Rep 9, 1219. 30718709 10.1038/s41598-018-38007-2 PMC6362094

[R113] Jiang F, Pan Z, Xu K, Tian L, Xie Y, Zhang X, Chen J, Dong B, Li Y, 2016. Screening of ABCA4 gene in a Chinese cohort with stargardt disease or cone-rod dystrophy with a report on 85 novel mutations. Invest. Ophthalmol. Vis. Sci 57, 145–152. 26780318 10.1167/iovs.15-18190

[R114] Julien S, Schraermeyer U, 2012. Lipofuscin can be eliminated from the retinal pigment epithelium of monkeys. Neurobiol. Aging 33, 2390–2397. 22244091 10.1016/j.neurobiolaging.2011.12.009

[R115] Jurgensmeier C, Bhosale P, Bernstein PS, 2007. Evaluation of 4-methylpyrazole as a potential therapeutic dark adaptation inhibitor. Curr. Eye Res 32, 911–915. 17963111 10.1080/02713680701616156

[R116] Kang Derwent JJ, Derlacki DJ, Hetling JR, Fishman GA, Birch DG, Grover S, Stone EM, Pepperberg DR, 2004. Dark adaptation of rod photoreceptors in normal subjects, and in patients with Stargardt disease and an ABCA4 mutation. Invest. Ophthalmol. Vis. Sci 45, 2447–2456. 15223829 10.1167/iovs.03-1178

[R117] Kaplan J, Gerber S, Larget-Piet D, Rozet JM, Dollfus H, Dufier JL, Odent S, Postel-Vinay A, Janin N, Briard ML, , 1993. A gene for Stargardťs disease (fundus flavimaculatus) maps to the short arm of chromosome 1. Nat. Genet 5, 308–311. 8275096 10.1038/ng1193-308

[R118] Keilhauer CN, Delori FC, 2006. Near-infrared autofluorescence imaging of the fundus: visualization of ocular melanin. Invest. Ophthalmol. Vis. Sci 47, 3556–3564. 16877429 10.1167/iovs.06-0122

[R119] Kellner S, Kellner U, Weber BH, Fiebig B, Weinitz S, Ruether K, 2009. Lipofuscin- and melanin-related fundus autofluorescence in patients with ABCA4-associated retinal dystrophies. Am. J. Ophthalmol 147, 895–902 902 e891. 19243736 10.1016/j.ajo.2008.12.023

[R120] Khan KN, Kasilian M, Mahroo OAR, Tanna P, Kalitzeos A, Robson AG, Tsunoda K, Iwata T, Moore AT, Fujinami K, Michaelides M, 2018. Early patterns of macular degeneration in ABCA4-associated retinopathy. Ophthalmology 125, 735–746. 29310964 10.1016/j.ophtha.2017.11.020 PMC5917070

[R121] Khan M, Cornells SS, Khan MI, Elmelik D, Manders E, Bakker S, Derks R, Neveling K, van de Vorst M, Gilissen C, Meunier I, Defoort S, Puech B, Devos A, Schulz HL, Stohr H, Grassmann F, Weber BHF, Dhaenens CM, Cremers FPM, 2019. Cost-effective molecular inversion probe-based ABCA4 sequencing reveals deep-intronic variants in Stargardt disease. Hum. Mutat 40, 1749–1759. 31212395 10.1002/humu.23787

[R122] Khan M, Cornells SS, del Pozo-Valero M, Whelan L, Runhart EH, Mishra K, Bults F, AlSwaiti Y, AlTabishi A, De Baere E, Banff S, Banin E, Bauwens M, Ben-Yosef T, Boon CJF, van den Born LI, Defoort S, Devos A, Dockery A, Dudakova L, Fakin A, Farrar GJ, Ferraz Sallum JM, Fujinami K, Gilissen C, Glavac D, Gorin MB, Greenberg J, Hayashi T, Hettinga Y, Hoischen A, Hoyng CB, Hufendiek K, Jägle H, Kamakari S, Karali M, Kellner U, Klaver CCW, Kousal B, Lamey T, MacDonald IM, Matynia A, McLaren T, Mena MD, Meunier I, Miller R, Newman H, Ntozini B, Oldak M, Pieterse M, Podhajcer OL, Puech B, Ramesar R, Rüther K, Salameh M, Vallim Salles M, Sharon D, Simonelli F, Spital G, Steehouwer M, Szaflik JP, Thompson JA, Thuillier C, Tracewska AM, van Zweeden M, Vincent AL, Zanlonghi X, Liskova P, Stöhr H, De Roach J, Ayuso C, Roberts L, Weber BHF, Dhaenens C-M, Cremers FPM, 2020. Resolving the dark matter of ABCA4 for 1,054 Stargardt disease probands through integrated genomics and transcriptomics. Genet. Med https://www.nature.com/articles/s41436-020-0787-4#article-info. 10.1038/s41436-020-0787-4 32307445

[R123] Kim HJ, Sparrow JR, 2018. Novel bisretinoids of human retina are lyso alkyl ether glycerophosphoethanolamine-bearing A2PE species. J. Lipid Res 59, 1620–1629. 29986955 10.1194/jlr.M084459 PMC6121928

[R124] Kjellstrom U, 2015. Reduced macular function in ABCA4 carriers. Mol. Vis 21, 767–782. 26261413 PMC4506055

[R125] Klein ML, Ferris FL 3rd, Francis PJ, Lindblad AS, Chew EY, Hamon SC, Ott J, 2010. Progression of geographic atrophy and genotype in age-related macular degeneration. Ophthalmology 117, 1554–1559. 20381870 10.1016/j.ophtha.2009.12.012 PMC2904435

[R126] Klevering BJ, Blankenagel A, Maugeri A, Cremers FP, Hoyng CB, Rohrschneider K, 2002. Phenotypic spectrum of autosomal recessive cone-rod dystrophies caused by mutations in the ABCA4 (ABCR) gene. Invest. Ophthalmol. Vis. Sci 43, 1980–1985. 12037008

[R127] Klien BA, Krill AE, 1967. Fundus flavimaculatus. Clinical, functional and histopathologic observations. Am. J. Ophthalmol 64, 3–23. 6028632

[R128] Kniazeva M, Chiang MF, Morgan B, Anduze AL, Zack DJ, Han M, Zhang K, 1999. A new locus for autosomal dominant stargardt-like disease maps to chromosome 4. Am. J. Hum. Genet 64, 1394–1399. 10205271 10.1086/302377 PMC1377876

[R129] Knudtson MD, Klein R, Klein BE, Lee KE, Meuer SM, Tomany SC, 2004. Location of lesions associated with age-related maculopathy over a 10-year period: the Beaver Dam Eye Study. Invest. Ophthalmol. Vis. Sci 45, 2135–2142. 15223787 10.1167/iovs.03-1085

[R130] Kong J, Kim SR, Binley K, Pata I, Doi K, Mannik J, Zernant-Rajang J, Kan O, Iqball S, Naylor S, Sparrow JR, Gouras P, Allikmets R, 2008. Correction of the disease phenotype in the mouse model of Stargardt disease by lentiviral gene therapy. Gene Ther 15, 1311–1320. 18463687 10.1038/gt.2008.78 PMC3110063

[R131] Kong X, Strauss RW, Michaelides M, Cideciyan AV, Sahel JA, Munoz B, West S, Scholl HP, ProgStar Study G, 2016. Visual acuity loss and associated risk factors in the retrospective progression of stargardt disease study (ProgStar report No. 2). Ophthalmology 123, 1887–1897. 27378015 10.1016/j.ophtha.2016.05.027

[R132] Kong X, Ho A, Munoz B, West S, Strauss RW, Jha A, Ervin A, Buzas J, Singh M, Hu Z, Cheetham J, Ip M, Scholl HPN, 2019. Reproducibility of measurements of retinal structural parameters using optical coherence tomography in stargardt disease. Transl Vis Sci Technol 8, 46. 10.1167/tvst.8.3.46 PMC6590092 31259091

[R133] Krishnan AK, Bedell HE, 2018. Functional changes at the preferred retinal locus in subjects with bilateral central vision loss. Graefes Arch. Clin. Exp. Ophthalmol 256, 29–37. 28971293 10.1007/s00417-017-3818-3

[R134] Kubota R, Al-Fayoumi S, Mallikaarjun S, Patil S, Bavik C, Chandler JW, 2014. Phase 1, dose-ranging study of emixustat hydrochloride (ACU-4429), a novel visual cycle modulator, in healthy volunteers. Retina 34, 603–609. 24056528 10.1097/01.iae.0000434565.80060.f8

[R135] Kuniyoshi K, Terasaki H, Arai M, Hirose T, 2014. Multifocal electroretinograms in Stargardťs disease/fundus flavimaculatus. Ophthalmologica 232, 118–125. 24970593 10.1159/000361056

[R136] Lambertus S, van Huet RA, Bax NM, Hoefsloot LH, Cremers FP, Boon CJ, Klevering BJ, Hoyng CB, 2015. Early-onset stargardt disease: phenotypic and genotypic characteristics. Ophthalmology 122, 335–344. 25444351 10.1016/j.ophtha.2014.08.032

[R137] Lambertus S, Lindner M, Bax NM, Mauschitz MM, Nadal J, Schmid M, Schmitz-Valckenberg S, den Hollander AI, Weber BH, Holz FG, van der Wilt GJ, Fleckenstein M, Hoyng CB, Foveal sparing Atrophy Study, 2016. Progression of late-onset stargardt disease. T.. Invest. Ophthalmol. Vis. Sci 57, 5186–5191. 10.1167/iovs.16-19833 27699414

[R138] Lang W, 1885. Central choroiditis with disseminated patches in remainder of fundus. Trans. Ophthalmol. Soc. U. K 5, 140–141.

[R139] Lee W, Noupuu K, Oll M, Duncker T, Burke T, Zernant J, Bearelly S, Tsang SH, Sparrow JR, Allikmets R, 2014. The external limiting membrane in early-onset Stargardt disease. Invest. Ophthalmol. Vis. Sci 55, 6139–6149. 25139735 10.1167/iovs.14-15126 PMC4184384

[R140] Lee W, Xie Y, Zernant J, Yuan B, Bearelly S, Tsang SH, Lupski JR, Allikmets R, 2016. Complex inheritance of ABCA4 disease: four mutations in a family with multiple macular phenotypes. Hum. Genet 135, 9–19. 26527198 10.1007/s00439-015-1605-y PMC4699863

[R141] Lee W, Schuerch K, Zernant J, Collison FT, Bearelly S, Fishman GA, Tsang SH, Sparrow JR, Allikmets R, 2017. Genotypic spectrum and phenotype correlations of ABCA4-associated disease in patients of south Asian descent. Eur. J. Hum. Genet 25, 735–743. 28327576 10.1038/ejhg.2017.13 PMC5477356

[R142] Lee W, Zernant J, Nagasaki T, Tsang SH, Allikmets R, 2018. Deep scleral exposure: a degenerative outcome of end-stage stargardt disease. Am. J. Ophthalmol 195, 16–25. 30055151 10.1016/j.ajo.2018.07.018 PMC6547128

[R143] Lee W, Paavo M, Zernant J, Stong N, Laurente Z, Bearelly S, Nagasaki T, Tsang SH, Goldstein DB, Allikmets R, 2019. Modification of the PROM1 disease phenotype by a mutation in ABCA4. Ophthalmic Genet 40, 369–375. 31576780 10.1080/13816810.2019.1660382 PMC6777736

[R144] Lenis TL, Hu J, Ng SY, Jiang Z, Sarfare S, Lloyd MB, Esposito NJ, Samuel W, Jaworski C, Bok D, Finnemann SC, Radeke MJ, Redmond TM, Travis GH, Radu RA, 2018. Expression of ABCA4 in the retinal pigment epithelium and its implications for Stargardt macular degeneration. Proc. Natl. Acad. Sci. U. S. A 115, El1120–El1127. 10.1073/pnas.1802519115 PMC6255167 30397118

[R145] Lewis RA, Shroyer NF, Singh N, Allikmets R, Hutchinson A, Li Y, Lupski JR, Leppert M, Dean M, 1999. Genotype/Phenotype analysis of a photoreceptor-specific ATP-binding cassette transporter gene, ABCR, in Stargardt disease. Am. J. Hum. Genet 64, 422–434. 9973280 10.1086/302251 PMC1377752

[R146] Lindblad AS, Lloyd PC, Clemons TE, Gensler GR, Ferris FL 3rd, Klein ML, Armstrong JR, Age-Related Eye Disease Study Research, 2009. Change in area of geographic atrophy in the Age-Related Eye Disease Study: AREDS report number 26. G. Arch. Ophthalmol 127, 1168–1174. 10.1001/archophthalmol.2009.198 PMC6500457 19752426

[R147] Lindner M, Lambertus S, Mauschitz MM, Bax NM, Kersten E, Luning A, Nadal J, Schmitz-Valckenberg S, Schmid M, Holz FG, Hoyng CB, Fleckenstein M, Foveal sparing Atrophy Study, 2017. Differential disease progression in atrophic age-related macular degeneration and late-onset stargardt disease. T.. Invest. Ophthalmol. Vis. Sci 58, 1001–1007. 10.1167/iovs.16-20980 28288486

[R148] Liu Q, Sabirzhanova I, Bergbower EAS, Yanda M, Guggino WG, Cebotaru L, 2019. The CFTR corrector, VX-809 (lumacaftor), rescues ABCA4 trafficking mutants: a potential treatment for stargardt disease. Cell. Physiol. Biochem 53, 400–412. 31403270 10.33594/000000146 PMC7027368

[R149] Lois N, Holder GE, Bunce C, Fitzke FW, Bird AC, 2001. Phenotypic subtypes of Stargardt macular dystrophy-fundus flavimaculatus. Arch. Ophthalmol 119, 359–369. 11231769 10.1001/archopht.119.3.359

[R150] Lois N, Halfyard AS, Bird AC, Holder GE, Fitzke FW, 2004. Fundus autofluorescence in Stargardt macular dystrophy-fundus flavimaculatus. Am. J. Ophthalmol 138, 55–63. 15234282 10.1016/j.ajo.2004.02.056

[R151] Lopez PF, Maumenee IH, de la Cruz Z, Green WR, 1990. Autosomal-dominant fundus flavimaculatus. Clinicopathologic correlation. Ophthalmology 97, 798–809. 2374685 10.1016/s0161-6420(90)32508-3

[R152] Lopez-Rubio S, Chacon-Camacho OF, Matsui R, Guadarrama-Vallejo D, Astiazaran MC, Zenteno JC, 2018. Retinal phenotypic characterization of patients with ABCA4 retinopathydue to the homozygous p.Alal773Val mutation. Mol. Vis 24, 105–114. 29422768 PMC5800431

[R153] Ma CJ, Lee W, Stong N, Zernant J, Chang S, Goldstein D, Nagasaki T, Allikmets R, 2019. Late-onset pattern macular dystrophy mimicking ABGA4 and PRPH2 disease is caused by a homozygous frameshift mutation in ROM1. Cold Spring Harb Mol Case Stud 5. 10.1101/mcs.a003624 PMC6549556 30630813

[R154] MacDonald IM, Sieving PA, 2018. Investigation of the effect of dietary docosahexaenoic acid (DHA) supplementation on macular function in subjects with autosomal recessive Stargardt macular dystrophy. Ophthalmic Genet 39, 477–486. 29912604 10.1080/13816810.2018.1484931

[R155] Maeda A, Maeda T, Golczak M, Palczewski K, 2008. Retinopathy in mice induced by disrupted all-trans-retinal clearance. J. Biol. Chem 283, 26684–26693. 18658157 10.1074/jbc.M804505200 PMC2546559

[R156] Maguire AM, Russell S, Wellman JA, Chung DC, Yu ZF, Tillman A, Wittes J, Pappas J, Elci O, Marshall KA, McCague S, Reichert H, Davis M, Simonelli F, Leroy BP, Wright JF, High KA, Bennett J, 2019. Efficacy, safety, and durability of voretigene neparvovec-rzyl in RPE65 mutation-associated inherited retinal dystrophy: results of phase 1 and 3 trials. Ophthalmology 126, 1273–1285. 31443789 10.1016/j.ophtha.2019.06.017

[R157] Maia-Lopes S, Silva ED, Silva MF, Reis A, Faria P, Castelo-Branco M, 2008. Evidence of widespread retinal dysfunction in patients with stargardt disease and morphologically unaffected carrier relatives. Invest. Ophthalmol. Vis. Sci 49, 1191–1199. 18326749 10.1167/iovs.07-1051

[R158] Makelainen S, Godia M, Hellsand M, Viluma A, Hahn D, Makdoumi K, Zeiss CJ, Mellersh C, Ricketts SL, Narfstrom K, Hallbook F, Ekesten B, Andersson G, Bergstrom TF, 2019. An ABCA4 loss-of-function mutation causes a canine form of Stargardt disease. PLoS Genet 15, e1007873. 30889179 10.1371/journal.pgen.1007873 PMC6424408

[R159] Martinez-Mir A, Paloma E, Allikmets R, Ayuso C, del Rio T, Dean M, Vilageliu L, Gonzalez-Duarte R, Balcells S, 1998. Retinitis pigmentosa caused by a homozygous mutation in the Stargardt disease gene ABCR. Nat. Genet 18, 11–12. 9425888 10.1038/ng0198-11

[R160] Mata NL, Tzekov RT, Liu X, Weng J, Birch DG, Travis GH, 2001. Delayed dark-adaptation and lipofuscin accumulation in abcr + /− mice: implications for involvement of ABCR In age-related macular degeneration. Invest. Ophthalmol. Vis. Sci 42, 1685–1690. 11431429

[R161] Maugeri A, van Driel MA, van de Pol DJ, Klevering BJ, van Haren FJ, Tijmes N, Bergen AA, Rohrschneider K, Blankenagel A, Pinckers AJ, Dahl N, Brunner HG, Deutman AF, Hoyng CB, Cremers FP, 1999. The 2588G->C mutation in the ABCR gene is a mild frequent founder mutation in the Western European population and allows the classification of ABCR mutations in patients with Stargardt disease. Am. J. Hum. Genet 64, 1024–1035. 10090887 10.1086/302323 PMC1377826

[R162] Maugeri A, Klevering BJ, Rohrschneider K, Blankenagel A, Brunner HG, Deutman AF, Hoyng CB, Cremers FP, 2000. Mutations in the ABCA4 (ABCR) gene are the major cause of autosomal recessive cone-rod dystrophy. Am. J. Hum. Genet 67, 960–966. 10958761 10.1086/303079 PMC1287897

[R163] Maugeri A, Flothmann K, Hemmrich N, Ingvast S, Jorge P, Paloma E, Patel R, Rozet JM, Tammur J, Testa F, Balcells S, Bird AC, Brunner HG, Hoyng CB, Metspalu A, Simonelli F, Allikmets R, Bhattacharya SS, D’Urso M, Gonzalez-Duarte R, Kaplan J, te Meerman GJ, Santos R, Schwartz M, Van Camp G, Wadelius C, Weber BH, Cremers FP, 2002. The ABCA4 2588G>C Stargardt mutation: single origin and increasing frequency from South-West to North-East Europe. Eur. J. Hum. Genet 10, 197–203. 11973624 10.1038/sj.ejhg.5200784

[R164] Maw MA, Corbeil D, Koch J, Hellwig A, Wilson-Wheeler JC, Bridges RJ, Kumaramanickavel G, John S, Nancarrow D, Roper K, Weigmann A, Huttner WB, Denton MJ, 2000. A frameshift mutation in prominin (mouse)-like 1 causes human retinal degeneration. Hum. Mol. Genet 9, 27–34. 10587575 10.1093/hmg/9.1.27

[R165] McBain VA, Townend J, Lois N, 2012. Progression of retinal pigment epithelial atrophy in stargardt disease. Am. J. Ophthalmol 154, 146–154. 22464366 10.1016/j.ajo.2012.01.019

[R166] McClements ME, Barnard AR, Singh MS, Charbel Issa P, Jiang Z, Radu RA, MacLaren RE, 2019. An AAV dual vector strategy ameliorates the stargardt phenotype in adult Abca4(-/-) mice. Hum. Gene Ther 30, 590–600. 30381971 10.1089/hum.2018.156 PMC6909730

[R167] Mehat MS, Sundaram V, Ripamonti C, Robson AG, Smith AJ, Borooah S, Robinson M, Rosenthal AN, Innes W, Weleber RG, Lee RWJ, Crossland M, Rubin GS, Dhillon B, Steel DHW, Anglade E, Lanza RP, Ali RR, Michaelides M, Bainbridge JWB, 2018. Transplantation of human embryonic stem cell-derived retinal pigment epithelial cells in macular degeneration. Ophthalmology 125, 1765–1775. 29884405 10.1016/j.ophtha.2018.04.037 PMC6195794

[R168] Melillo P, Testa F, Rossi S, Di Iorio V, Orrico A, Auricchio A, Simonelli F, 2016. En face spectral-domain optical coherence tomography for the monitoring of lesion area progression in stargardt disease. Invest. Ophthalmol. Vis. Sci 57, OCT247–252. 27409479 10.1167/iovs.15-18751 PMC4968920

[R169] Michaelides M, Holder GE, Bradshaw K, Hunt DM, Moore AT, 2005. Cone-rod dystrophy, intrafamilial variability, and incomplete penetrance associated with the R172W mutation in the peripherin/RDS gene. Ophthalmology 112, 1592–1598. 16019073 10.1016/j.ophtha.2005.04.004

[R170] Molday RS, 2015. Insights into the molecular properties of ABCA4 and its role in the visual cycle and stargardt disease. Prog Mol Biol Transl Sci 134, 415–431. 26310168 10.1016/bs.pmbts.2015.06.008

[R171] Molday LL, Molday Rs AR, 2016. Localization and Functional Analysis of ABCA4 Variants Associated with Stargardt Disease, Association for Research in Vision and Ophthalmology. Seattle, WA.

[R172] Molday LL, Wahl D, Sarunic MV, Molday RS, 2018. Localization and functional characterization of the p.Asn965Ser (N965S) ABCA4 variant in mice reveal pathogenic mechanisms underlying Stargardt macular degeneration. Hum. Mol. Genet 27, 295–306. 29145636 10.1093/hmg/ddx400 PMC5886264

[R173] Muller PL, Gliem M, Mangold E, Bolz HJ, Finger RP, McGuinness M, Betz C, Jiang Z, Weber BH, MacLaren RE, Holz FG, Radu RA, Charbel Issa P, 2015. Monoallelic ABGA4 mutations appear insufficient to cause retinopathy: a quantitative auto fluorescence study. Invest. Ophthalmol. Vis. Sci 56, 8179–8186. 26720470 10.1167/iovs.15-17629 PMC5110240

[R174] Muller PL, Fimmers R, Gliem M, Holz FG, Charbel Issa P, 2017. Choroidal alterations in abca4-related retinopathy. Retina 37, 359–367. 27414126 10.1097/IAE.0000000000001169

[R175] Nakao T, Tsujikawa M, Sawa M, Gomi F, Nishida K, 2012. Foveal sparing in patients with Japanese Stargard’s disease and good visual acuity. Jpn. J. Ophthalmol 56, 584–588. 22956038 10.1007/s10384-012-0172-1

[R176] Nassisi M, Mohand-Said S, Andrieu C, Antonio A, Condroyer C, Mejecase C, Varin J, Wohlschlegel J, Dhaenens CM, Sahel JA, Zeitz C, Audo I, 2019. Prevalence of ABCA4 deep-intronic variants and related phenotype in an unsolved “One-Hit” cohort with stargardt disease. Int. J. Mol. Sci 20, E5053. 31614660 10.3390/ijms20205053 PMC6829239

[R177] Noupuu K, Lee W, Zernant J, Tsang SH, Allikmets R, 2014. Structural and genetic assessment of the ABCA4-associated optical gap phenotype. Invest. Ophthalmol. Vis. Sci 55, 7217–7226. 25301883 10.1167/iovs.14-14674 PMC4228863

[R178] Noupuu K, Lee W, Zernant J, Greenstein VC, Tsang S, Allikmets R, 2016. Recessive Stargardt disease phenocopying hydroxychloroquine retinopathy. Graefes Arch. Clin. Exp. Ophthalmol 254, 865–872. 26311262 10.1007/s00417-015-3142-8 PMC4769982

[R179] Oner A, Gonen ZB, Sevim DG, Smim Kahraman N, Unlu M, 2018. Suprachoroidal adipose tissue-derived mesenchymal stem cell implantation in patients with dry-type Age-related macular degeneration and stargardťs macular dystrophy: 6-month follow-up results of a phase 2 study. Cell. Reprogr 20, 329–336. 10.1089/cell.2018.0045 31251672

[R180] Paavo M, Zhao J, Kim HJ, Lee W, Zernant J, Cai C, Allikmets R, Tsang SH, Sparrow JR, 2018. Mutations in gpr143/OA1 and ABCA4 inform interpretations of short-wavelength and near-infrared fundus auto fluorescence. Invest. Ophthalmol. Vis. Sci 59, 2459–2469. 29847651 10.1167/iovs.18-24213 PMC5959512

[R181] Paavo M, Lee W, Allikmets R, Tsang S, Sparrow JR, 2019. Photoreceptor cells as a source of fundus autofluorescence in recessive Stargardt disease. J. Neurosci. Res 97, 98–106. 29701254 10.1002/jnr.24252 PMC6532423

[R182] Pang CE, Suqin Y, Sherman J, Freund KB, 2015. New insights into Stargardt disease with multimodal imaging. Ophthalmic Surg Lasers Imaging Retina 46, 257–261. 25707054 10.3928/23258160-20150213-09

[R183] Papermaster DS, Converse CA, Zorn M, 1976. Biosynthetic and immunochemical characterization of large protein in frog and cattle rod outer segment membranes. Exp. Eye Res 23, 105–115. 976361 10.1016/0014-4835(76)90194-9

[R184] Parfitt DA, Lane A, Ramsden CM, Carr AJ, Munro PM, Jovanovic K, Schwarz N, Kanuga N, Muthiah MN, Hull S, Gallo JM, da Cruz L, Moore AT, Hardcastle AJ, Coffey PJ, Cheetham ME, 2016. Identification and correction of mechanisms underlying inherited blindness in human iPSC-derived optic cups. Cell Stem Cell 18, 769–781. 27151457 10.1016/j.stem.2016.03.021 PMC4899423

[R185] Parish CA, Hashimoto M, Nakanishi K, Dillon J, Sparrow J, 1998. Isolation and one-step preparation of A2E and iso-A2E, fluorophores from human retinal pigment epithelium. Proc. Natl. Acad. Sci. U. S. A 95, 14609–14613. 9843937 10.1073/pnas.95.25.14609 PMC24497

[R186] Park JC, Collison FT, Fishman GA, Allikmets R, Zernant J, Liu M, McAnany JJ, 2015. Objective analysis of hyperreflective outer retinal bands imaged by optical coherence tomography in patients with stargardt disease. Invest. Ophthalmol. Vis. Sci 56, 4662–4667. 26207301 10.1167/iovs.15-16955 PMC4516018

[R187] Parker MA, Choi D, Erker LR, Pennesi ME, Yang P, Chegarnov EN, Steinkamp PN, Schlechter CL, Dhaenens CM, Mohand-Said S, Audo I, Sahel J, Weleber RG, Wilson DJ, 2016. Test-retest variability of functional and structural parameters in patients with stargardt disease participating in the SAR422459 gene therapy trial. Transl Vis Sci Technol 5, 10. 10.1167/tvst.5.5.10 PMC5054761 27730010

[R188] Parodi MB, Iacono P, Triolo G, La Spina C, Zucchiatti I, Cicinelli MV, Borrelli E, Manitto MP, Martina E, Bandello F, 2015. Morpho-functional correlation of fundus autofluorescence in Stargardt disease. Br. J. Ophthalmol 99, 1354–1359. 25837607 10.1136/bjophthalmol-2014-306237

[R189] Passerini I, Sodi A, Giambene B, Mariottini A, Menchini U, Torricelli F, 2010. Novel mutations in of the ABCR gene in Italian patients with Stargardt disease. Eye 24, 158–164. 19265867 10.1038/eye.2009.35

[R190] Pellegrini M, Acquistapace A, Oldani M, Cereda MG, Giani A, Cozzi M, Staurenghi G, 2016. Dark atrophy: an optical coherence tomography angiography study. Ophthalmology 123, 1879–1886. 27448830 10.1016/j.ophtha.2016.05.041

[R191] Piccardi M, Fadda A, Martelli F, Marangoni D, Magli A, Minnella AM, Bertelli M, Di Marco S, Bisti S, Falsini B, 2019. Antioxidant saffron and central retinal function in ABCA4-related stargardt macular dystrophy. Nutrients 11. 10.3390/nu11102461 PMC6835540 31618812

[R192] Quazi F, Molday RS, 2013. Differential phospholipid substrates and directional transport by ATP-binding cassette proteins ABCA1, ABCA7, and ABCA4 and disease-causing mutants. J. Biol. Chem 288, 34414–34426. 24097981 10.1074/jbc.M113.508812 PMC3843056

[R193] Quazi F, Molday RS, 2014. ATP-binding cassette transporter ABCA4 and chemical isomerization protect photoreceptor cells from the toxic accumulation of excess 11-cis-retinal. Proc. Natl. Acad. Sci. U. S. A Ill, 5024–5029. 10.1073/pnas.1400780111 PMC3977269 24707049

[R194] Quazi F, Lenevich S, Molday RS, 2012. ABCA4 is an N-retinylidene-phosphatidy-lethanolamine and phosphatidylethanolamine importer. Nat. Commun 3, 925. 22735453 10.1038/ncomms1927 PMC3871175

[R195] Querques G, Leveziel N, Benhamou N, Voigt M, Soubrane G, Souied EH, 2006. Analysis of retinal flecks in fundus flavimaculatus using optical coherence tomography. Br. J. Ophthalmol 90, 1157–1162. 16754647 10.1136/bjo.2006.094136 PMC1857370

[R196] Querques G, Prato R, Iaculli C, Voigt M, Delle Noci N, Coscas G, Soubrane G, Souied EH, 2008. Correlation of visual function impairment and OCT findings in patients with Stargardt disease and fundus flavimaculatus. Eur. J. Ophthalmol 18, 239–247. 18320517 10.1177/112067210801800212

[R197] Racz B, Varadi A, Kong J, Allikmets R, Pearson PG, Johnson G, Cioffi CL, Petrukhin K, 2018. A non-retinoid antagonist of retinol-binding protein 4 rescues phenotype in a model of Stargardt disease without inhibiting the visual cycle. J. Biol. Chem 293, 11574–11588. 29871924 10.1074/jbc.RA118.002062 PMC6065170

[R198] Rees HA, Liu DR, 2018. Base editing: precision chemistry on the genome and transcriptome of living cells. Nat. Rev. Genet 19, 770–788. 30323312 10.1038/s41576-018-0059-1 PMC6535181

[R199] Riveiro-Alvarez R, Valverde D, Lorda-Sanchez I, Trujillo-Tiebas MJ, Cantalapiedra D, Vallespin E, Aguirre-Lamban J, Ramos C, Ayuso C, 2007. Partial paternal uniparental disomy (UPD) of chromosome 1 in a patient with Stargardt disease. Mol. Vis 13, 96–101. 17277736 PMC2553007

[R200] Riveiro-Alvarez R, Lopez-Martinez MA, Zernant J, Aguirre-Lamban J, Cantalapiedra D, Avila-Fernandez A, Gimenez A, Lopez-Molina MI, Garcia-Sandoval B, Bianco-Kelly F, Corton M, Tatu S, Fernandez-San Jose P, Trujillo-Tiebas MJ, Ramos C, Allikmets R, Ayuso C, 2013. Outcome of ABCA4 disease-associated alleles in autosomal recessive retinal dystrophies: retrospective analysis in 420 Spanish families. Ophthalmology 120, 2332–2337. 23755871 10.1016/j.ophtha.2013.04.002 PMC3808491

[R201] Rivera A, White K, Stohr H, Steiner K, Hemmrich N, Grimm T, Jurklies B, Lorenz B, Scholl HP, Apfelstedt-Sylla E, Weber BH, 2000. A comprehensive survey of sequence variation in the ABCA4 (ABCR) gene in Stargardt disease and age-related macular degeneration. Am. J. Hum. Genet 67, 800–813. 10958763 10.1086/303090 PMC1287885

[R202] Rosenberg T, Klie F, Garred P, Schwartz M, 2007. N965S is a common ABCA4 variant in Stargardt-related retinopathies in the Danish population. Mol. Vis 13, 1962–1969. 17982420

[R203] Rotenstreich Y, Fishman GA, Anderson RJ, 2003. Visual acuity loss and clinical observations in a large series of patients with Stargardt disease. Ophthalmology 110, 1151–1158. 12799240 10.1016/S0161-6420(03)00333-6

[R204] Rozet JM, Gerber S, Ghazi I, Perrault I, Ducroq D, Souied E, Cabot A, Dufier JL, Munnich A, Kaplan J, 1999. Mutations of the retinal specific ATP binding transporter gene (ABCR) in a single family segregating both autosomal recessive retinitis pigmentosa RP19 and Stargardt disease: evidence of clinical heterogeneity at this locus. J. Med. Genet 36, 447–451. 10874631 PMC1734380

[R205] Runhart EH, Sangermano R, Cornells SS, Verheij J, Plomp AS, Boon CJF, Lugtenberg D, Roosing S, Bax NM, Blokland EAW, Jacobs-Camps MHM, van der Velde-Visser SD, Pott JR, Rohrschneider K, Thiadens A, Klaver CCW, van den Born LI, Hoyng CB, Cremers FPM, 2018. The common ABCA4 variant p.Asnl868Ile shows nonpenetrance and variable expression of stargardt disease when present in trans with severe variants. Invest. Ophthalmol. Vis. Sci 59, 3220–3231. 29971439 10.1167/iovs.18-23881

[R206] Runhart EH, Valkenburg D, Cornells SS, Khan M, Sangermano R, Albert S, Bax NM, Astuti GDN, Gilissen C, Pott JR, Verheij J, Blokland EAW, Cremers FPM, van den Born LI, Hoyng CB, 2019. Late-onset stargardt disease due to mild, deep-intronic ABCA4 alleles. Invest. Ophthalmol. Vis. Sci 60, 4249–4256. 31618761 10.1167/iovs.19-27524

[R207] Sabirzhanova I, Lopes Pacheco M, Rapino D, Grover R, Handa JT, Guggino WB, Cebotaru L, 2015. Rescuing trafficking mutants of the ATP-binding cassette protein, ABCA4, with small molecule correctors as a treatment for stargardt eye disease. J. Biol. Chem 290, 19743–19755. 26092729 10.1074/jbc.M115.647685 PMC4528136

[R208] Sachdeva MM, Eliott D, 2016. Stem cell-based therapy for diseases of the retinal pigment epithelium: from bench to bedside. Semin. Ophthalmol 31, 25–29. 26959126 10.3109/08820538.2015.1115253

[R209] Salvatore S, Fishman GA, McAnany JJ, Genead MA, 2014. Association of dark-adapted visual function with retinal structural changes in patients with Stargardt disease. Retina 34, 989–995. 24280667 10.1097/IAE.0000000000000022 PMC4181837

[R210] Sangermano R, Bax NM, Bauwens M, van den Born LI, De Baere E, Garanto A, Collin RW, Goercharn-Ramlal AS, den Engelsman-van Dijk AH, Rohrschneider K, Hoyng CB, Cremers FP, Albert S, 2016. Photoreceptor progenitor mRNA analysis reveals exon skipping resulting from the ABCA4 c.5461–10T> C mutation in Stargardt disease. Ophthalmology 123, 1375–1385. 26976702 10.1016/j.ophtha.2016.01.053

[R211] Sangermano R, Khan M, Cornells SS, Richelle V, Albert S, Garanto A, Elmelik D, Qamar R, Lugtenberg D, van den Born LI, Collin RWJ, Cremers FPM, 2018. ABCA4 midigenes reveal the full splice spectrum of all reported noncanonical splice site variants in Stargardt disease. Genome Res 28, 100–110. 29162642 10.1101/gr.226621.117 PMC5749174

[R212] Sangermano R, Garanto A, Khan M, Runhart EH, Bauwens M, Bax NM, van den Born LI, Khan MI, Cornells SS, Verheij J, Pott JR, Thiadens A, Klaver CCW, Puech B, Meunier I, Naessens S, Arno G, Fakin A, Carss KJ, Raymond FL, Webster AR, Dhaenens CM, Stohr H, Grassmann F, Weber BHF, Hoyng CB, De Baere E, Albert S, Collin RWJ, Cremers FPM, 2019. Deep-intronic ABCA4 variants explain missing heritability in Stargardt disease and allow correction of splice defects by antisense oligonucleotides. Genet. Med 21, 1751–1760. 30643219 10.1038/s41436-018-0414-9 PMC6752325

[R213] Scholl HP, Besch D, Vonthein R, Weber BH, Apfelstedt-Sylla E, 2002. Alterations of slow and fast rod ERG signals in patients with molecularly confirmed Stargardt disease type 1. Invest. Ophthalmol. Vis. Sci 43, 1248–1256. 11923272

[R214] Schonbach EM, Ibrahim MA, Strauss RW, Birch DG, Cideciyan AV, Hahn GA, Ho A, Kong X, Nasser F, Sunness JS, Zrenner E, Sadda SR, West SK, Scholl HPN, Progression of Stargardt Disease Study, 2017a. Fixation location and stability using the MP-1 microperimeter in stargardt disease: ProgStar report No. 3. G. Ophthalmol Retina 1, 68–76. 10.1016/j.oret.2016.08.009 31047397

[R215] Schonbach EM, Wolfson Y, Strauss RW, Ibrahim MA, Kong X, Munoz B, Birch DG, Cideciyan AV, Hahn GA, Nittala M, Sunness JS, Sadda SR, West SK, Scholl HPN, ProgStar Study G, 2017b. Macular Sensitivity Measured with Microperimetry in Stargardt Disease in the Progression of Atrophy Secondary to Stargardt Disease (ProgStar) Study: Report No. 7. JAMA Ophthalmol 135. pp. 696–703. 28542693 10.1001/jamaophthalmol.2017.1162 PMC6584711

[R216] Schonbach EM, Strauss RW, Kong X, Munoz B, Ibrahim MA, Sunness JS, Birch DG, Hahn GA, Nasser F, Zrenner E, Sadda SR, West SK, Scholl HPN, ProgStar Study G, 2018. Longitudinal changes of fixation location and stability within 12 Months in stargardt disease: ProgStar report No. 12. Am. J. Ophthalmol 193, 54–61. 29890160 10.1016/j.ajo.2018.06.003 PMC7083180

[R217] Schorderet DF, Iouranova A, Favez T, Tiab L, Escher P, 2013. IROme, a new high-throughput molecular tool for the diagnosis of inherited retinal dystrophies. BioMed Res. Int 2013, 198089. 23484092 10.1155/2013/198089 PMC3591198

[R218] Schulz HL, Grassmann F, Kellner U, Spital G, Ruther K, Jagle H, Hufendiek K, Rating P, Huchzermeyer C, Baier MJ, Weber BH, Stohr H, 2017. Mutation spectrum of the ABCA4 gene in 335 stargardt disease patients from a multicenter German cohort-impact of selected deep intronic variants and common SNPs. Invest. Ophthalmol. Vis. Sci 58, 394–403. 28118664 10.1167/iovs.16-19936 PMC5270621

[R219] Schwartz SD, Hubschman JP, Heilwell G, Franco-Cardenas V, Pan CK, Ostrick RM, Mickunas E, Gay R, Klimanskaya I, Lanza R, 2012. Embryonic stem cell trials for macular degeneration: a preliminary report. Lancet 379, 713–720. 22281388 10.1016/S0140-6736(12)60028-2

[R220] Schwartz SD, Regillo CD, Lam BL, Eliott D, Rosenfeld PJ, Gregori NZ, Hubschman JP, Davis JL, Heilwell G, Spirn M, Maguire J, Gay R, Bateman J, Ostrick RM, Morris D, Vincent M, Anglade E, Del Priore LV, Lanza R, 2015. Human embryonic stem cell-derived retinal pigment epithelium in patients with age-related macular degeneration and Stargardťs macular dystrophy: follow-up of two open-label phase 1/2 studies. Lancet 385, 509–516. 25458728 10.1016/S0140-6736(14)61376-3

[R221] Sciezynska A, Ozieblo D, Ambroziak AM, Korwin M, Szulborski K, Krawczynski M, Stawinski P, Szaflik J, Szaflik JP, Ploski R, Oldak M, 2016. Next-generation sequencing of ABCA4: high frequency of complex alleles and novel mutations in patients with retinal dystrophies from Central Europe. Exp. Eye Res 145, 93–99. 26593885 10.1016/j.exer.2015.11.011

[R222] Shankar SP, Hughbanks-Wheaton DK, Birch DG, Sullivan LS, Conneely KN, Bowne SJ, Stone EM, Daiger SP, 2016. Autosomal dominant retinal dystrophies caused by a founder splice site mutation, C.828 + 3A>T, in PRPH2 and protein haplotypes in trans as modifiers. Invest. Ophthalmol. Vis. Sci 57, 349–359. 26842753 10.1167/iovs.15-16965 PMC4736744

[R223] Sharon D, Ben-Yosef T, Goldenberg-Cohen N, Pras E, Gradstein L, Soudry S, Mezer E, Zur D, Abbasi AH, Zeitz C, Cremers FPM, Khan MI, Levy J, Rotenstreich Y, Birk OS, Ehrenberg M, Leibu R, Newman H, Shomron N, Banin E, Perlman I, 2020. A nationwide genetic analysis of inherited retinal diseases in Israel as assessed by the Israeli inherited retinal disease consortium (IIRDC). Hum. Mutat 41, 140–149. 31456290 10.1002/humu.23903

[R224] Shroyer NF, Lewis RA, Lupski JR, 2000. Complex inheritance of ABCR mutations in Stargardt disease: linkage disequilibrium, complex alleles, and pseudodominance. Hum. Genet 106, 244–248. 10746567 10.1007/s004390051034

[R225] Shroyer NF, Lewis RA, Lupski JR, 2001a. Analysis of the ABCR (ABCA4) gene in 4-aminoquinoline retinopathy: is retinal toxicity by chloroquine and hydroxychloroquine related to Stargardt disease? Am. J. Ophthalmol 131, 761–766. 11384574 10.1016/s0002-9394(01)00838-8

[R226] Shroyer NF, Lewis RA, Yatsenko AN, Lupski JR, 2001b. Null missense ABCR (ABCA4) mutations in a family with stargardt disease and retinitis pigmentosa. Invest. Ophthalmol. Vis. Sci 42, 2757–2761. 11687513

[R227] Simon WA, Herrmann M, Klein T, Shin JM, Huber R, Senn-Bilfinger J, Postius S, 2007. Soraprazan: setting new standards in inhibition of gastric acid secretion. J. Pharmacol. Exp. Therapeut 321, 866–874. 10.1124/jpet.107.120428 17369284

[R228] Sisk RA, Leng T, 2014. Multimodal imaging and multifocal electroretinography demonstrate autosomal recessive Stargardt disease may present like occult macular dystrophy. Retina 34, 1567–1575. 24743636 10.1097/IAE.0000000000000136

[R229] Sodi A, Mucciolo DP, Cipollini F, Murro V, Caporossi O, Virgili G, Rizzo S, 2016. En face OCT in Stargardt disease. Graefes Arch. Clin. Exp. Ophthalmol 254, 1669–1679. 26743751 10.1007/s00417-015-3254-1

[R230] Sohocki MM, Sullivan LS, Mintz-Hittner HA, Birch D, Heckenlively JR, Freund CL, McInnes RR, Daiger SP, 1998. A range of clinical phenotypes associated with mutations in CRX, a photoreceptor transcription-factor gene. Am. J. Hum. Genet 63, 1307–1315. 9792858 10.1086/302101 PMC1377541

[R231] Song H, Rossi EA, Latchney L, Bessette A, Stone E, Hunter JJ, Williams DR, Chung M, 2015a. Cone and rod loss in Stargardt disease revealed by adaptive optics scanning light ophthalmoscopy. JAMA Ophthalmol 133, 1198–1203. 26247787 10.1001/jamaophthalmol.2015.2443 PMC4600048

[R232] Song WK, Park KM, Kim HJ, Lee JH, Choi J, Chong SY, Shim SH, Del Priore LV, Lanza R, 2015b. Treatment of macular degeneration using embryonic stem cell-derived retinal pigment epithelium: preliminary results in Asian patients. Stem Cell Reports 4, 860–872. 25937371 10.1016/j.stemcr.2015.04.005 PMC4437471

[R233] Souied EH, Ducroq D, Gerber S, Ghazi I, Rozet JM, Perrault I, Munnich A, Dufier JL, Coscas G, Soubrane G, Kaplan J, 1999. Age-related macular degeneration in grandparents of patients with Stargardt disease: genetic study. Am. J. Ophthalmol 128, 173–178. 10458172 10.1016/s0002-9394(99)00145-2

[R234] Sparrow JR, Boulton M, 2005. RPE lipofuscin and its role in retinal pathobiology. Exp. Eye Res 80, 595–606. 15862166 10.1016/j.exer.2005.01.007

[R235] Sparrow JR, Yamamoto K, 2012. The bisretinoids of RPE lipofuscin: a complex mixture. Adv. Exp. Med. Biol 723, 761–767. 22183404 10.1007/978-1-4614-0631-0_97 PMC11829280

[R236] Sparrow JR, Hicks D, Hamel CP, 2010. The retinal pigment epithelium in health and disease. Curr. Mol. Med 10, 802–823. 21091424 10.2174/156652410793937813 PMC4120883

[R237] Sparrow JR, Gregory-Roberts E, Yamamoto K, Blonska A, Ghosh SK, Ueda K, Zhou J, 2012. The bisretinoids of retinal pigment epithelium. Prog. Retin. Eye Res 31, 121–135. 22209824 10.1016/j.preteyeres.2011.12.001 PMC3288746

[R238] Sparrow JR, Blonska A, Flynn E, Duncker T, Greenberg JP, Secondi R, Ueda K, Delori FC, 2013. Quantitative fundus autofluorescence in mice: correlation with HPLC quantitation of RPE lipofuscin and measurement of retina outer nuclear layer thickness. Invest. Ophthalmol. Vis. Sci 54, 2812–2820. 23548623 10.1167/iovs.12-11490 PMC3632269

[R239] Sparrow JR, Marsiglia M, Allikmets R, Tsang S, Lee W, Duncker T, Zernant J, 2015. Flecks in recessive stargardt disease: short-wavelength Autofluorescence, near-infrared autofluorescence, and optical coherence tomography. Invest. Ophthalmol. Vis. Sci 56, 5029–5039. 26230768 10.1167/iovs.15-16763 PMC4525681

[R240] Sparrow JR, Duncker T, Schuerch K, Paavo M, de Carvalho JRL Jr., 2020. Lessons learned from quantitative fundus autofluorescence. Prog. Retin. Eye Res 74,100774. 31472235 10.1016/j.preteyeres.2019.100774 PMC7561015

[R241] Stargardt K, 1909. Über familiäre, progressive Degeneration in der Maculagegend des Auges. Albrecht von Graefes Arch Klin Ophthalmology 71, 534–550.

[R242] Steinberg RH, Wood I, Hogan MJ, 1977. Pigment epithelial ensheathment and phagocytosis of extrafoveal cones in human retina. Philos. Trans. R. Soc. Lond. B Biol. Sci 277, 459–474. 16301 10.1098/rstb.1977.0028

[R243] Stenirri S, Battistella S, Fermo I, Manitto MP, Martina E, Brancato R, Ferrari M, Cremonesi L, 2006. De novo deletion removes a conserved motif in the C-terminus of ABCA4 and results in cone-rod dystrophy. Clin. Chem. Lab. Med 44, 533–537. 16681420 10.1515/CCLM.2006.116

[R244] Stone EM, Nichols BE, Kimura AE, Weingeist TA, Drack A, Sheffield VC, 1994. Clinical features of a Stargardt-like dominant progressive macular dystrophy with genetic linkage to chromosome 6q. Arch. Ophthalmol 112, 765–772. 8002834 10.1001/archopht.1994.01090180063036

[R245] Strauss RW, Ho A, Munoz B, Cideciyan AV, Sahel JA, Sunness JS, Birch DG, Bernstein PS, Michaelides M, Traboulsi EI, Zrenner E, Sadda S, Ervin AM, West S, Scholl HP, Progression of Stargardt Disease Study, G., 2016. The natural history of the progression of atrophy secondary to stargardt disease (ProgStar) studies: design and baseline characteristics: ProgStar report No. 1. Ophthalmology 123, 817–828. 26786511 10.1016/j.ophtha.2015.12.009

[R246] Strauss RW, Munoz B, Ho A, Jha A, Michaelides M, Cideciyan AV, Audo I, Birch DG, Hariri AH, Nittala MG, Sadda S, West S, Scholl HPN, ProgStar Study G, 2017a. Progression of stargardt disease as determined by fundus autofluorescence in the retrospective progression of stargardt disease study (ProgStar report No. 9). JAMA Ophthalmol 135, 1232–1241. 29049437 10.1001/jamaophthalmol.2017.4152 PMC5710470

[R247] Strauss RW, Munoz B, Ho A, Jha A, Michaelides M, Mohand-Said S, Cideciyan AV, Birch D, Hariri AH, Nittala MG, Sadda S, Scholl HPN, ProgStar Study G, 2017b. Incidence of Atrophic Lesions in Stargardt Disease in the Progression of Atrophy Secondary to Stargardt Disease (ProgStar) Study: Report No. 5. JAMA Ophthalmol 135, 687–695. 28542697 10.1001/jamaophthalmol.2017.1121 PMC5710205

[R248] Sun H, Molday RS, Nathans J, 1999. Retinal stimulates ATP hydrolysis by purified and reconstituted ABCR, the photoreceptor-specific ATP-binding cassette transporter responsible for Stargardt disease. J. Biol. Chem 274, 8269–8281. 10075733 10.1074/jbc.274.12.8269

[R249] Sunness JS, Steiner JN, 2008. Retinal function and loss of autofluorescence in stargardt disease. Retina 28, 794–800. 18536594 10.1097/IAE.0b013e31816690bd

[R250] Sunness JS, Ziegler MD, Applegate CA, 2006. Issues in quantifying atrophic macular disease using retinal autofluorescence. Retina 26, 666–672. 16829810 10.1097/01.iae.0000236472.56195.e9

[R251] Tanaka K, Lee W, Zernant J, Schuerch K, Ciccone L, Tsang SH, Sparrow JR, Allikmets R, 2018. The rapid-onset chorioretinopathy phenotype of ABCA4 disease. Ophthalmology 125, 89–99. 28947085 10.1016/j.ophtha.2017.07.019 PMC5846118

[R252] Tanna P, Georgiou M, Strauss RW, Ali N, Kumaran N, Kalitzeos A, Fujinami K, Michaelides M, 2019. Cross-sectional and longitudinal assessment of the ellipsoid zone in childhood-onset stargardt disease. Transl Vis Sci Technol 8, 1. 10.1167/tvst.8.2.1 PMC6397016 30834176

[R253] Testa F, Rossi S, Sodi A, Passerini I, Di Iorio V, Della Corte M, Banff S, Surace EM, Menchini U, Auricchio A, Simonelli F, 2012. Correlation between photoreceptor layer integrity and visual function in patients with Stargardt disease: implications for gene therapy. Invest. Ophthalmol. Vis. Sci 53, 4409–4415. 22661472 10.1167/iovs.11-8201 PMC4625823

[R254] Testa F, Melillo P, Di Iorio V, Orrico A, Attanasio M, Rossi S, Simonelli F, 2014. Macular function and morphologic features in juvenile stargardt disease: longitudinal study. Ophthalmology 121, 2399–2405. 25097154 10.1016/j.ophtha.2014.06.032 PMC4252720

[R255] Teussink MM, Lee MD, Smith RT, van Huet RA, Klaver CC, Klevering BJ, Theelen T, Hoyng CB, 2015. The effect of light deprivation in patients with Stargardt disease. Am. J. Ophthalmol 159, 964–972 e962. 25681002 10.1016/j.ajo.2015.02.004

[R256] Tosha C, Gorin MB, Nusinowitz S, 2010. Test-retest reliability and inter-ocular symmetry of multi-focal electroretinography in Stargardt disease. Curr. Eye Res 35, 63–72. 20021256 10.3109/02713680903374224

[R257] Tracewska AM, Kocyla-Karczmarewicz B, Rafalska A, Murawska J, Jakubaszko-Jablonska J, Rydzanicz M, Stawinski P, Ciara E, Khan MI, Henkes A, Hoischen A, Gilissen C, van de Vorst M, Cremers FPM, Ploski R, Chrzanowska KH, 2019. Genetic spectrum of ABCA4-associated retinal degeneration in Poland. Genes (Basel) 10, E959. 31766579 10.3390/genes10120959 PMC6947411

[R258] Trapani I, 2019. Adeno-associated viral vectors as a tool for large gene delivery to the retina. Genes (Basel) 10, E287. 30970639 10.3390/genes10040287 PMC6523333

[R259] Trapani I, Colella P, Sommella A, Iodice C, Cesi G, de Simone S, Marrocco E, Rossi S, Giunti M, Palfi A, Farrar GJ, Polishchuk R, Auricchio A, 2014. Effective delivery of large genes to the retina by dual AAV vectors. EMBO Mol. Med 6, 194–211. 24150896 10.1002/emmm.201302948 PMC3927955

[R260] Trapani I, Toriello E, de Simone S, Colella P, Iodice C, Polishchuk EV, Sommella A, Colecchi L, Rossi S, Simonelli F, Giunti M, Bacci ML, Polishchuk RS, Auricchio A, 2015. Improved dual AAV vectors with reduced expression of truncated proteins are safe and effective in the retina of a mouse model of Stargardt disease. Hum. Mol. Genet 24, 6811–6825. 26420842 10.1093/hmg/ddv386 PMC4634381

[R261] Tsybovsky Y, Palczewski K, 2014. Expression, purification and structural properties of ABC transporter ABCA4 and its individual domains. Protein Expr. Purif 97, 50–60. 24583180 10.1016/j.pep.2014.02.010 PMC4033903

[R262] Tsybovsky Y, Molday RS, Palczewski K, 2010. The ATP-binding cassette transporter ABCA4: structural and functional properties and role in retinal disease. Adv. Exp. Med. Biol 703, 105–125. 20711710 10.1007/978-1-4419-5635-4_8 PMC2930353

[R263] Tsybovsky Y, Orban T, Molday RS, Taylor D, Palczewski K, 2013. Molecular organization and ATP-induced conformational changes of ABCA4, the photoreceptor-specific ABC transporter. Structure 21, 854–860. 23562398 10.1016/j.str.2013.03.001 PMC3654078

[R264] Turcotte Gauthier MT, 2010. Etude clinique et genetique d’une nouvelle forme d’ataxie spinocerebelleuse pure associee a l’erythrokeratodermie, Faculte de Medecine. Univ. Montreal

[R265] Valverde D, Riveiro-Alvarez R, Bernal S, Jaakson K, Baiget M, Navarro R, Ayuso C, 2006. Microarray-based mutation analysis of the ABCA4 gene in Spanish patients with Stargardt disease: evidence of a prevalent mutated allele. Mol. Vis 12, 902–908. 16917483

[R266] van Driel MA, Maugeri A, Klevering BJ, Hoyng CB, Cremers FP, 1998. ABCR unites what ophthalmologists divide(s). Ophthalmic Genet 19, 117–122. 9810566 10.1076/opge.19.3.117.2187

[R267] van Huet RA, Bax NM, Westeneng-Van Haaften SC, Muhamad M, Zonneveld-Vrieling MN, Hoefsloot LH, Cremers FP, Boon CJ, Klevering BJ, Hoyng CB, 2014. Foveal sparing in Stargardt disease. Invest. Ophthalmol. Vis. Sci 55, 7467–7478. 25324290 10.1167/iovs.13-13825

[R268] Vandenbroucke T, Buyl R, De Zaeytijd J, Bauwens M, Uvijls A, De Baere E, Leroy BP, 2015. Colour vision in stargardt disease. Ophthalmic Res 54, 181–194. 26492201 10.1159/000438906

[R269] Vazquez-Dominguez I, Garanto A, Collin RWJ, 2019. Molecular Therapies for Inherited Retinal Diseases-Current Standing, Opportunities and Challenges. Genes, Basel 10. 10.3390/genes10090654 PMC6770110 31466352

[R270] Verbakel SK, van Huet RAC, Boon CJF, den Hollander AI, Collin RWJ, Klaver CCW, Hoyng CB, Roepman R, Klevering BJ, 2018. Non-syndromic retinitis pigmentosa. Prog. Retin. Eye Res 66, 157–186. 29597005 10.1016/j.preteyeres.2018.03.005

[R271] Verdina T, Tsang SH, Greenstein VC, Zernant J, Sodi A, Lima LH, Chang S, Allikmets R, Menchini U, 2012. Functional analysis of retinal flecks in stargardt disease. J. Clin. Exp. Ophthalmol 3. 10.4172/2155-9570.1000233 PMC3882688 24409374

[R272] von Ruckmann A, Fitzke FW, Bird AC, 1995. Distribution of fundus autofluorescence with a scanning laser ophthalmoscope. Br. J. Ophthalmol 79, 407–412. 7612549 10.1136/bjo.79.5.407 PMC505125

[R273] von Ruckmann A, Fitzke FW, Bird AC, 1997. In vivo fundus autofluorescence in macular dystrophies. Arch. Ophthalmol 115, 609–615. 9152128 10.1001/archopht.1997.01100150611006

[R274] Webster AR, Heon E, Lotery AJ, Vandenburgh K, Casavant TL, Oh KT, Beck G, Fishman GA, Lam BL, Levin A, Heckenlively JR, Jacobson SG, Weleber RG, Sheffield VC, Stone EM, 2001. An analysis of allelic variation in the ABCA4 gene. Invest. Ophthalmol. Vis. Sci 42, 1179–1189. 11328725

[R275] Weng J, Mata NL, Azarian SM, Tzekov RT, Birch DG, Travis GH, 1999. Insights into the function of Rim protein in photoreceptors and etiology of Stargardťs disease from the phenotype in abcr knockout mice. Cell 98, 13–23. 10412977 10.1016/S0092-8674(00)80602-9

[R276] Wolock CJ, Stong N, Ma CJ, Nagasaki T, Lee W, Tsang SH, Kamalakaran S, Goldstein DB, Allikmets R, 2019. A case-control collapsing analysis identifies retinal dystrophy genes associated with ophthalmic disease in patients with no pathogenic ABCA4 variants. Genet. Med 21, 2336–2344. 30926958 10.1038/s41436-019-0495-0 PMC6768764

[R277] Wu Y, Fishkin NE, Pande A, Pande J, Sparrow JR, 2009. Novel lipofuscin bisretinoids prominent in human retina and in a model of recessive Stargardt disease. J. Biol. Chem 284, 20155–20166. 19478335 10.1074/jbc.M109.021345 PMC2740442

[R278] Yamamoto K, Yoon KD, Ueda K, Hashimoto M, Sparrow JR, 2011. A novel bisretinoid of retina is an adduct on glycerophosphoethanolamine. Invest. Ophthalmol. Vis. Sci 52, 9084–9090. 22039245 10.1167/iovs.11-8632 PMC3231846

[R279] Yanik M, Muller B, Song F, Gall J, Wagner F, Wende W, Lorenz B, Stieger K, 2017. In vivo genome editing as a potential treatment strategy for inherited retinal dystrophies. Prog. Retin. Eye Res 56, 1–18. 27623223 10.1016/j.preteyeres.2016.09.001

[R280] Yatsenko AN, Shroyer NF, Lewis RA, Lupski JR, 2001. Late-onset Stargardt disease is associated with missense mutations that map outside known functional regions of ABCR (ABCA4). Hum. Genet 108, 346–355. 11379881 10.1007/s004390100493

[R281] Yatsenko AN, Shroyer NF, Lewis RA, Lupski JR, 2003. An ABCA4 genomic deletion in patients with Stargardt disease. Hum. Mutat 21, 636–644. 12754711 10.1002/humu.10219

[R282] Young RW, 1967. The renewal of photoreceptor cell outer segments. J. Cell Biol 33, 61–72. 6033942 10.1083/jcb.33.1.61 PMC2107286

[R283] Young RW, Bok D, 1969. Participation of the retinal pigment epithelium in the rod outer segment renewal process. J. Cell Biol 42, 392–403. 5792328 10.1083/jcb.42.2.392 PMC2107669

[R284] Zernant J, Collison FT, Lee W, Fishman GA, Noupuu K, Yuan B, Cai C, Lupski JR, Yannuzzi LA, Tsang SH, Allikmets R, 2014a. Genetic and clinical analysis of ABCA4-associated disease in African American patients. Hum. Mutat 35,1187–1194. 25066811 10.1002/humu.22626 PMC4283973

[R285] Zernant J, Xie YA, Ayuso C, Riveiro-Alvarez R, Lopez-Martinez MA, Simonelli F, Testa F, Gorin MB, Strom SP, Bertelsen M, Rosenberg T, Boone PM, Yuan B, Ayyagari R, Nagy PL, Tsang SH, Gouras P, Collison FT, Lupski JR, Fishman GA, Allikmets R, 2014b. Analysis of the ABCA4 genomic locus in Stargardt disease. Hum. Mol. Genet 23, 6797–6806. 25082829 10.1093/hmg/ddu396 PMC4245042

[R286] Zernant J, Lee W, Collison FT, Fishman GA, Sergeev YV, Schuerch K, Sparrow JR, Tsang SH, Allikmets R, 2017. Frequent hypomorphic alleles account for a significant fraction of ABCA4 disease and distinguish it from age-related macular degeneration. J. Med. Genet 54, 404–412. 28446513 10.1136/jmedgenet-2017-104540 PMC5786429

[R287] Zernant J, Lee W, Nagasaki T, Collison FT, Fishman GA, Bertelsen M, Rosenberg T, Gouras P, Tsang SH, Allikmets R, 2018. Extremely hypomorphic and severe deep intronic variants in the ABCA4 locus result in varying Stargardt disease phenotypes. Cold Spring Harb Mol Case Stud 4. 10.1101/mcs.a002733 PMC6071568 29848554

[R288] Zhang Q, Zulfiqar F, Xiao X, Riazuddin SA, Ahmad Z, Caruso R, MacDonald I, Sieving P, Riazuddin S, Hejtmancik JF, 2007. Severe retinitis pigmentosa mapped to 4pl5 and associated with a novel mutation in the PROM1 gene. Hum. Genet 122, 293–299. 17605048 10.1007/s00439-007-0395-2

[R289] Zhang N, Tsybovsky Y, Kolesnikov AV, Rozanowska M, Swider M, Schwartz SB, Stone EM, Palczewska G, Maeda A, Kefalov VJ, Jacobson SG, Cideciyan AV, Palczewski K, 2015. Protein misfolding and the pathogenesis of ABCA4-associated retinal degenerations. Hum. Mol. Genet 24, 3220–3237. 25712131 10.1093/hmg/ddv073 PMC4424957

[R290] Zolnikova IV, Strelnikov VV, Skvortsova NA, Tanas AS, Barh D, Rogatina EV, Egorova IV, Levina DV, Demenkova ON, Prikaziuk EG, Ivanova ME, 2017. Stargardt disease-associated mutation spectrum of a Russian Federation cohort. Eur. J. Med. Genet 60, 140–147. 10.1016/j.ejmg.2016.12.002 27939946

